# Magnesium Nutrient Application Induces Metabolomics and Physiological Responses in Mulberry (*Morus alba*) Plants

**DOI:** 10.3390/ijms24119650

**Published:** 2023-06-02

**Authors:** Xin Jin, Michael Ackah, Lei Wang, Frank Kwarteng Amoako, Yisu Shi, Lionnelle Gyllye Essoh, Jianbin Li, Qiaonan Zhang, Haonan Li, Weiguo Zhao

**Affiliations:** 1 Jiangsu Key Laboratory of Sericultural Biology and Biotechnology, School of Biotechnology, Jiangsu University of Science and Technology, Zhenjiang 212100, China; 202310033@stu.just.edu.cn (X.J.); 201310006@stu.just.edu.cn (L.W.); shiyisu1297@just.edu.cn (Y.S.); 211611800006@stu.just.edu.cn (L.G.E.); 211111801108@stu.just.edu.cn (J.L.); 202310011@stu.just.edu.cn (Q.Z.); 221111802126@stu.just.edu.cn (H.L.); 2 School of Food and Biological Engineering, Jiangsu University, Zhenjiang 212013, China; 3 Institute of Plant Nutrition and Soil Science, Kiel University, Hermann-Rodewald-Straße 2, 24118 Kiel, Germany; stu225468@mail.uni-kiel.de

**Keywords:** *Morus alba*, magnesium nutrition, physiology, LC-MS, metabolomics, untargeted approach

## Abstract

Mulberry (*Morus alba*) is a significant plant with numerous economic benefits; however, its growth and development are affected by nutrient levels. A high level of magnesium (Mg) or magnesium nutrient starvation are two of the significant Mg factors affecting plant growth and development. Nevertheless, *M. alba’s* metabolic response to different Mg concentrations is unclear. In this study, different Mg concentrations, optimal (3 mmol/L), high (6 mmol/L and 9 mmol/L), or low (1 and 2 mmol/L) and deficient (0 mmol/L), were applied to *M. alba* for three weeks to evaluate their effects via physiological and metabolomics (untargeted; liquid chromatography–mass spectrometry (LC-MS)) studies. Several measured physiological traits revealed that Mg deficiency and excess Mg altered net photosynthesis, chlorophyll content, leaf Mg content and fresh weight, leading to remarkable reductions in the photosynthetic efficiency and biomass of mulberry plants. Our study reveals that an adequate supply of the nutrient Mg promoted the mulberry’s physiological response parameters (net photosynthesis, chlorophyll content, leaf and root Mg content and biomass). The metabolomics data show that different Mg concentrations affect several differential metabolite expressions (DEMs), particularly fatty acyls, flavonoids, amino acids, organic acid, organooxygen compounds, prenol lipids, coumarins, steroids and steroid derivatives, cinnamic acids and derivatives. An excessive supply of Mg produced more DEMs, but negatively affected biomass production compared to low and optimum supplies of Mg. The significant DEMs correlated positively with mulberry’s net photosynthesis, chlorophyll content, leaf Mg content and fresh weight. The mulberry plant’s response to the application of Mg used metabolites, mainly amino acids, organic acids, fatty acyls, flavonoids and prenol lipids, in the KEGG (Kyoto Encyclopedia of Genes and Genomes) pathways. These classes of compounds were mainly involved in lipid metabolism, amino acid metabolism, energy metabolism, the biosynthesis of other secondary metabolites, the biosynthesis of other amino acids, the metabolism of cofactors and vitamin pathways, indicating that mulberry plants respond to Mg concentrations by producing a divergent metabolism. The supply of Mg nutrition was an important factor influencing the induction of DEMs, and these metabolites were critical in several metabolic pathways related to magnesium nutrition. This study provides a fundamental understanding of DEMs in *M. alba’s* response to Mg nutrition and the metabolic mechanisms involved, which may be critical to the mulberry genetic breeding program.

## 1. Introduction

In most Chinese provinces, mulberry (*Morus* spp.) is cultivated primarily for its leaves and fruits. Moreover, mulberry leaves are the only food source for domestic silkworms (*Bombyx mori* L.), making them an indispensable tree species for the sericulture sector [1]. The genus *Morus* contains 68 species that flourish in various climates, from temperate to tropical, predominantly in Asia, with one-third (24) of the species growing naturally in China [2]. In addition to its historic use in silkworm rearing, mulberry remains a promising pioneer tree species in marginal environments [3]. *M. alba* leaves are thought to exhibit antioxidant, anti-inflammatory and anti-allergic qualities due to the number of bioactive phytochemicals, such as polyphenolic compounds, triterpenoids and anthocyanins, among others [1,4]. Plants rely on most of these phytochemicals to survive a wide range of biotic and abiotic stressors in their microenvironments. These include but are not limited to a limited availability of nutrition, harsh temperatures, high light intensity, and microbial attack [5,6]. Globally, considerable crop output losses can be attributed to abiotic variables, which are major environmental limiting factors in today’s agricultural methods [5]. Crop intensification, in addition to the widespread use of chemical fertilizers, frequently results in salt accumulation in the soil, resulting in nutrient imbalances [7]. A high level of salinity can change the physiological and biochemical systems that defend plants from secondary stress situations such as oxidative stress, osmotic imbalance, and water and nutrient deficiencies, negatively affecting plant growth. Numerous minerals related to salt accumulation, including magnesium (Mg) and sodium (Na), have been described recently [8].

Magnesium (Mg) is a macronutrient that is essential for plant growth and development. Mg is the most abundant free divalent cation in the cytosol, and as a cofactor of various enzymes involved in carbon metabolism, it is essential for chlorophyll formation and carbon fixation in plants [6]. Mg has a significantly smaller ionic radius and a relatively greater hydrated radius compared to other soil cations [9]. Consequently, Mg is readily leached in tropical and subtropical regions with acidic soils. In addition, soil aluminum (Al) solubilization resulting from acidity reduces plant Mg availability [9]. This is because Al and Mg have similar hydrated radii of 0.480 nm and 0.428 nm, respectively, allowing competition for Mg binding sites on plant roots. Such situations indicate that plants often suffer from Mg deficiency in tropical and subtropical regions where soils are more acidic [10]. Indeed, Mg deficiency is typically correlated with visible interveinal chlorosis, growth reduction and crop yield. In addition, Mg deficiency affects photosynthesis. For instance, it was reported that Mg deficiency downregulates the photosynthetic rate in the leaves of rice, cabbage and maize [9].

Both Mg deficit and excess stress conditions impact plant morphogenesis, development and yield via distinct pathways [11]. Furthermore, how Mg-induced stress circumstances play a crucial role in activating the plant stress response, which includes the phytohormones, downstream metabolism (C-fixation), ion absorption and antioxidant processes, ref. [12] is largely unclear. For this reason, it is imperative to investigate the metabolome under the conditions of various applications of Mg (deficiency, undersupply, optimal supply and oversupply) in plants.

Plant scientists are increasingly turning to metabolomics to better understand how plants react physiologically to environmental stresses such as drought, salt and heat [13,14]. Metabolomics comprises a vast array of analytical techniques for identifying organism molecular metabolomic materials [1]. The metabolome is the ultimate final product of the genome and is made up of many tiny molecules (≤2000 Da) that play a role in modulating practically every biomolecular mechanism, such as stress tolerance and the response to environmental signals [15]. As a result, metabolomics is an essential method for studying the mechanisms through which plants respond to stress in certain environments [16]. Although it is imperative to investigate the metabolome under various Mg applications in plants because of the essential role Mg plays in plant growth and development, no work has been carried out thus far on the metabolomics profile of the mulberry response to Mg supply and the associated stress coupled with the physiological responses [17].

The aims of this study were to investigate Mg application (in low, excess and sufficient amounts) and its deficiency in mulberry plants, using physiological parameters and an untargeted LC-MS (on the global level) analysis to elucidate the physiological responses and biochemical compounds and their various pathways that may be crucial to the mulberry plant’s response to the application of Mg. We hypothesized that the levels of Mg supply would alter the metabolic pathways of mulberry differently. This study will be fundamental for understanding the mulberry metabolome profile under the conditions of nutrient application, which may be crucial to mulberry genetic breeding programs.

## 2. Results

### 2.1. Plant Biomass Production and Mg Content in Tissues

**Figure 1:**
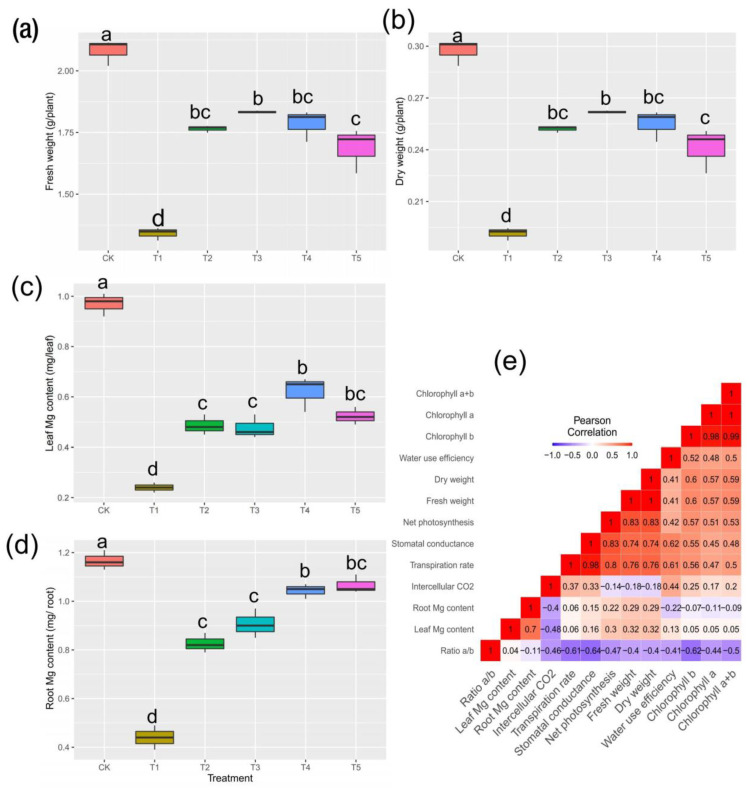
Determination of physiological parameters in mulberry response to magnesium (MgSO_4_) supply. Plants were grown in pot (35 cm) experiment for 20 days with different levels of magnesium (MgSO_4_) treatment. (**a**) Fresh weight, (**b**) dry weight, (**c**) leaf Mg content and (**d**) root Mg content, (**e**) Correlation heat map. Data are the means of three biological replicates. Data with different letters are significantly different, as measured by Tukey’s test (*p* ≤ 0.05). CK; sufficiency; T1; 0 mmol/L; T2; 1 mmol/L; T3; 2 mmol/L; T4. 6 mmol/L; T5; 9 mmol/L of Mg.

Studies on the mulberry plant’s response to Mg imbalances are limited [17]. To account for how mulberry responded to Mg stresses, we observed the morpho-physiological parameters of *M. alba*. The present results indicate highly significant differences at *p* < 0.05 (Figure 1a,b) in Mg treatments supplied at different levels. The results show that the growth of the mulberry plant is highly prone to Mg deprivation and low and excessive supplies of Mg; however, the sufficient Mg (CK) treatment provided outstanding Mg levels in both fresh and dry biomass (Figure 1a,b). Relative to T1 (no Mg) and T5 (excess Mg), the CK-treated mulberry plants experienced increases of 55% and 33% in fresh weight and 58% and 25% in dry weight, respectively. The low supplies of Mg (T2 and T3) increased the mulberry biomass (fresh and dry weight) compared to the higher supplies of Mg (T4 and T5) (Figure 1a,b). Moreover, T2 and T4 did not induce any significant variation in the accumulation of fresh and dry matter. Our current study failed to measure the impact of Mg supply and deficiency on mulberry root growth and biomass, which will be considered in a follow-up study. The mulberry plants exhibited remarkable variations in leaf and root Mg contents, depicting a normal distribution pattern in the root (Figure 1d). However, the Mg content distribution in the leaves showed a contrasting pattern (Figure 1c). Although T4 and T5 showed higher Mg contents than T2, T3 and T1, which was normal as T4 and T5 had higher Mg concentrations in the treatment compared to T2 and T3, the Mg content in CK was higher than in the low, deficient and toxic groups. The same trend was observed in the root tissue. Surprisingly, no significant variations were found in T2 and T3 in either the leaf or root tissues. However, the CK group showed an increase of 168% relative to T1 and 9.37% relative to T5 in the roots and 304% relative to T1 and 85% relative to T5 in the leaves, with the root Mg contents being higher than the leaf Mg contents. Nevertheless, positive correlations were observed between the biomass and Mg accumulation in both the leaves and roots (r = 0.32 and 0.29, respectively), although they were nonsignificant (Figure 1e).

### 2.2. Physiological Responses to Mg and Leaf Pigments

**Figure 2:**
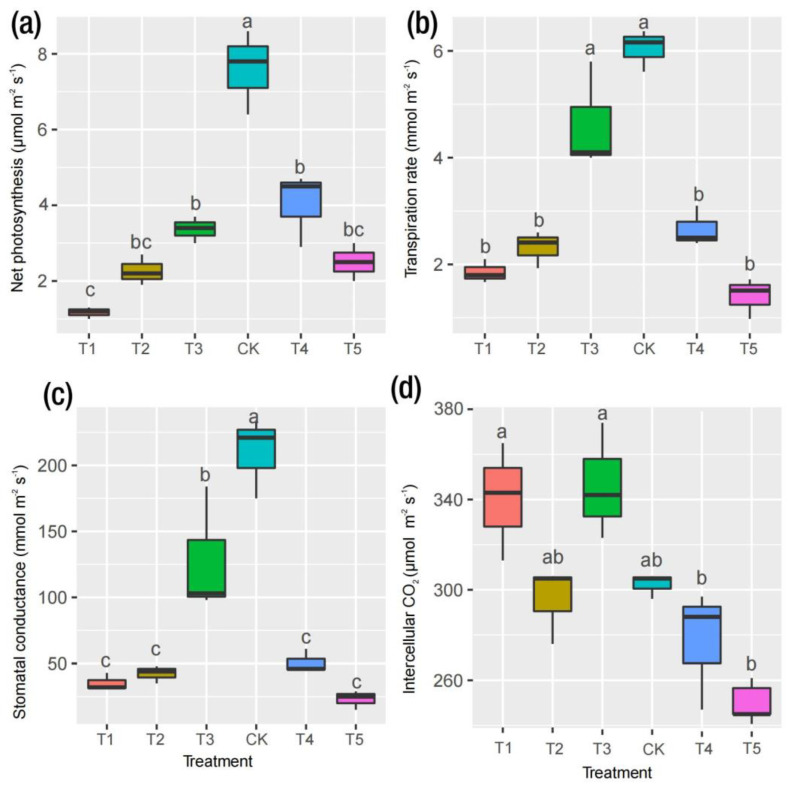
Photosynthetic apparatus determination in *M. alba’s* response to magnesium (MgSO_4_) applications. Plants were grown in pot (35 cm) experiment for 20 days with different levels of magnesium (MgSO_4_) treatment. (**a**) Net photosynthetic rate (Pn), (**b**) transpiration rate (Tr), (**c**) stomatal conductance (Gs) and (**d**) intercellular CO_2_ concentration (Ci). Data are the means of three biological replicates. Data with different letters are significantly different, as measured by Tukey’s test (*p* ≤ 0.05).

As shown in Figure 2a, in the Mg deficiency group, a low or high supply of Mg significantly reduced the P_n_ of mulberry plants, but an increase an in P_n_ was observed for the CK treatment group. Similar trends were observed for the Tr and Gs in which CK outperformed the other treatments (Figure 2b,c). The present results suggest that Mg treatment did not severely affect physiological efficiency among the T1, T2, T4 and T5 treatments in mulberry plants. However, CK was lower compared to T1 and T3 in terms of their Ci, with the T5 treatment having the most reduced Ci value. The availability of Mg promotes photosynthetic efficiency, which largely impacts plant biomass production [18]. The positive and highly significant correlation between the gas exchange parameters (P_n_, Gs and Tr) and plant biomass (measured in fresh and dry weights) were not coincidental but were rather indicative of a strong relationship between these variables, as demonstrated by their robust correlation coefficients (r = 0.83, 0.74 and 0.76, respectively) (Figure 1e).

**Figure 3:**
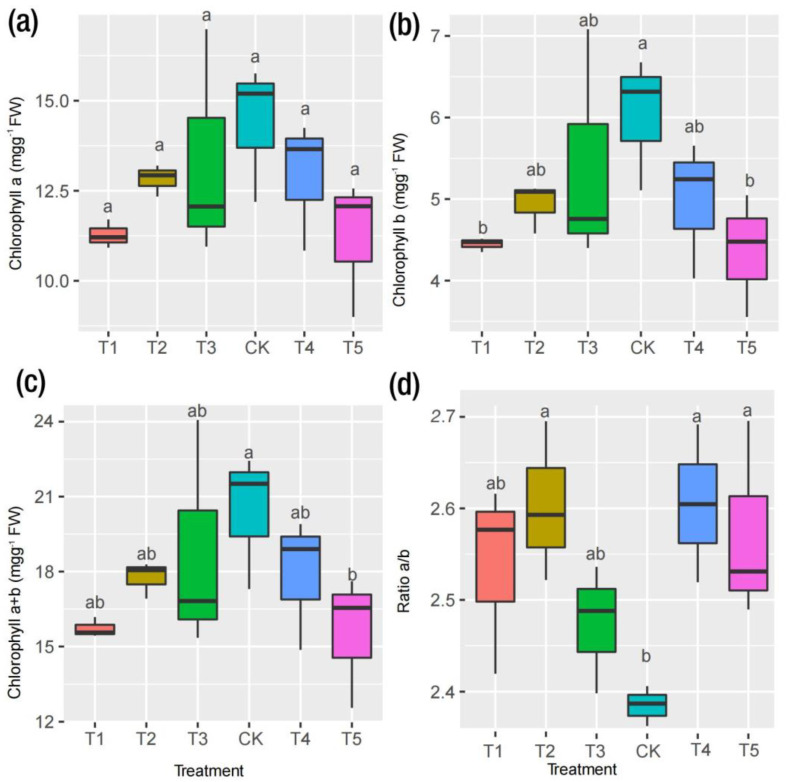
Determination of chlorophyll content in mulberry response to magnesium (MgSO_4_) supplied to *M. alba* grown in a pot (35 cm) experiment for 20 days with different levels of magnesium (MgSO_4_) treatment. (**a**) Chlorophyll *a*, (**b**) Chlorophyll *b*, (**c**) Chlorophyll *a*+*b* and (**d**) ratio of chlorophyll *a* and *b* (*a*/*b*). Data are the means of three biological replicates. Data with different letters are significantly different, as measured by Tukey’s test (*p* ≤ 0.05).

**Figure 4:**
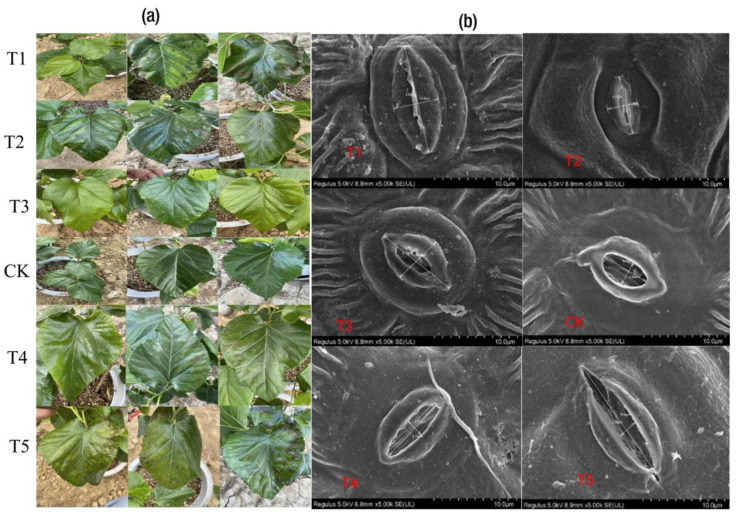
Physio-morphological parameters of mulberry plant with Mg supply and deficiency. (**a**) Mg stress in *M. alba*. T1–T5 indicating detailed responses of leaves showing Mg symptoms (**b**) Field emission scanning electron micrographs showing stomatal opening under different levels.

As depicted in Figure 3a, irrespective of the Mg treatment, the Mg supply did not affect the Chl *a* concentration significantly. However, the reverse was observed for Chl *b* and Chl *a*+*b* (Figure 3b,c). Compared with CK, T1 and T5 decreased remarkably in terms of Chl *b*, resulting in the leaves showing symptoms of chlorosis and necrotic spots, as observed in T1 and T5 (Figure 4a; T1–T5). When comparing the leaves’ symptoms, however, the plants receiving low supplies of Mg (T2 and T3) showed some signs of leaf chlorosis, but the symptoms were not prevalent, as observed in cases of Mg deficiency and toxicity. In the plants receiving an optimum Mg supply (CK,) the mulberry leaves showed normal growth without any symptoms on the leaves. The summation of Chl *a* and *b* (Chl *a*+*b*) only demonstrated significant variation when CK was compared with T5, indicating that Mg supplied in excess (toxicity) affected the chlorophyll or leaf pigment when compared to Mg deficiency (T1) (Figure 3c). However, Chl *a*/*b* demonstrated a significant increase for all levels of Mg (T1, T2, T4 and T5) except CK, which exhibited the lowest level in this regard (Figure 3d). Interestingly, remarkable correlations were observed between chlorophyll contents, biomass accumulation and gas exchange traits (Figure 1e). For instance, Chl *a*, *b* and *a*+*b* correlated positively with Ci (r = 0.17, 0.25, 0.20), Tr (r = 0.47, 0.56, 0.50), Gs (r = 0.45, 0.55, 0.48), P_n_ (r = 0.51, 0.57, 0.53) and biomass (i.e., fresh and dry weights) (r = 0.57, 0.60, 0.59), respectively. However, the ratio of Chl *a*/*b* correlated negatively with all physiological parameters (Figure 1e). A field emission scanning electron microscopy analysis was carried out on the mulberry plants leaves to observe their stomatal apertures (Figure 4b). In the CK samples, the mulberry plants’ stomatal apertures opened widely (Figure 4b). We observed that in the Mg deficiency group, the stomatal apertures completely closed as seen in T1 (Figure 4b). However, when the Mg concentration increased gradually from deficiency, the stomatal apertures opened, although the sizes were decreased (T2 and T3). At high Mg concentrations, we observed that the stomatal aperture opened, and the stomatal size was increased in T5 compared to T4 (Figure 4b).

### 2.3. Metabolic Profiles in M. alba Leaves: Responses to Various Applications of Mg

**Figure 5:**
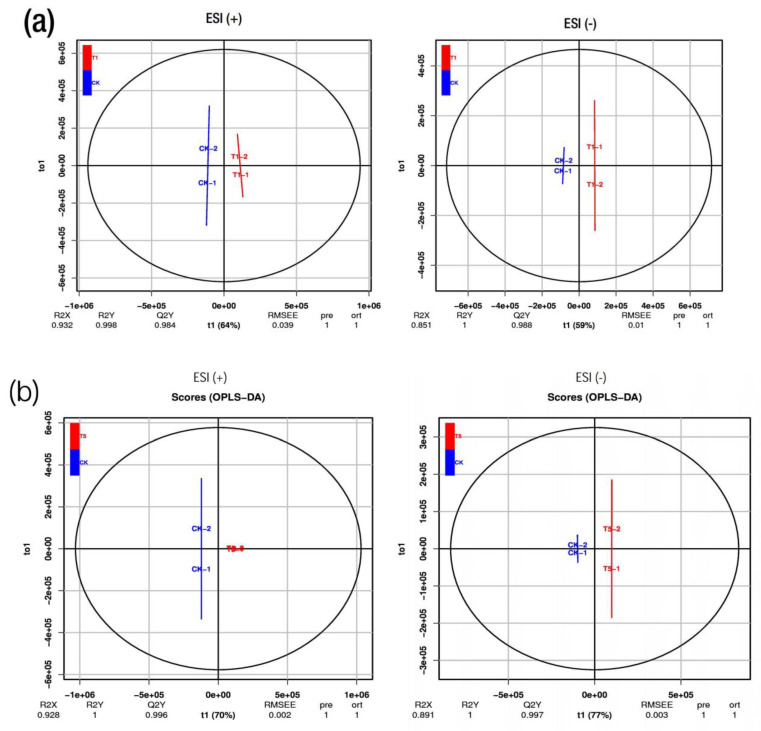
Orthogonal partial least squares discriminant analysis (OPLS-DA) scores of the metabolites (**a**) CK-T1 (**b**) CK-T5. The OPLS-DA models are R2X, R2Y and Q2, where R2X and R2Y represent the interpretation rate of the built model to the X and Y matrices, respectively, and Q2 represents the prediction ability of the model. The closer these three indicators are to 1, the more stable and reliable the model is. Q2 > 0.5 indicates that the model has good prediction ability, and Q2 > 0.9 indicates that the model is excellent. ESI (+), positive ion mode; ESI (−), negative ion mode.

To investigate the metabolite profiles concerning the phenotypic parameters of the mulberry under the conditions of various Mg applications, six mulberry leaf samples with two replicates each were grouped into five categories between the samples. These groupings included CK_T1, CK_T2, CK_T3, CK_T4 and CK_T5, and the metabolites in these groupings were analyzed via an untargeted LC-MS to identify the overall metabolomic changes in the mulberry leaves. After the data processing, the results indicate that 1022 and 731 metabolites were obtained at the positive ion mode (ESI +) and negative ion mode (ESI −), respectively, in the samples after a quality control check (Table S1). The obtained data from the two ion modes were then normalized and subjected to multivariate statistical analyses using PCA, PLS-DA and OPLS-DA via the R package gmodels [19]. As indicated in Figure S1, the PCA score plot analysis shows that the *M. alba* leaf extracts exhibited considerable metabolite variations following the different Mg treatments. For example, Figure S1a shows that variations of 61.1% and 60.9% occurred in ESI (+) and ESI (−), respectively, between CK and T1. In addition, variations of 71% and 70.2% occurred between CK and T2 (Figure S1b). Furthermore, a similar trend of such variations occurred in all the PCA score plots in the comparison of CK with the other Mg treatments (Figure S1c–e). PLS-DA and OPLS-DA analyses in the ESI (+) or ESI (−) models were performed on the differences between CK and the other groups (T1-T5), which yielded similar categorization results (Figure S2). Moreover, the OPLS-DA score analysis showed a clearer distinct separation between the optimum-Mg-treated samples (CK) in comparison with T1, the low-Mg-treated samples (T2 and T3), and the excess-Mg-treated samples (T4 and T5). The OPLS-DA score plot found 64% and 59% of the total variance in the ESI (+) and ESI (−) between CK and T1, with satisfactory prediction model parameters of Q2 = 0.984 and 0.988 and *p*-values of 0.039 and 0.01 in ESI (+) and ESI (−), respectively (Figure 5a). Additionally, between CK and T2, total variances of 78% and 79% were found, with Q2 = 0.999 and 0.997 and *p*-values of 0.008 and 0.009 in ESI (+) and ESI (−), respectively (Figure S3a). A similar trend was recorded for CK-T3 (Figure S3b). The OPLS-DA score plot displays a distinct clustering pattern similar to the PCA, indicating the disparity in metabolomics between CK and T4 and T5 (Figure 5b and Figure S3c). The differences between the treatments suggest a reliable and satisfactory discrimination model, indicating biochemical changes between CK and T1–T5. The permutation tests of the OPLS-DA score plots are shown in Figure S4.

### 2.4. Analysis of the Differential Metabolites in M. alba Leaves: Responses to Various Mg Applications and Deficiency

**Figure 6:**
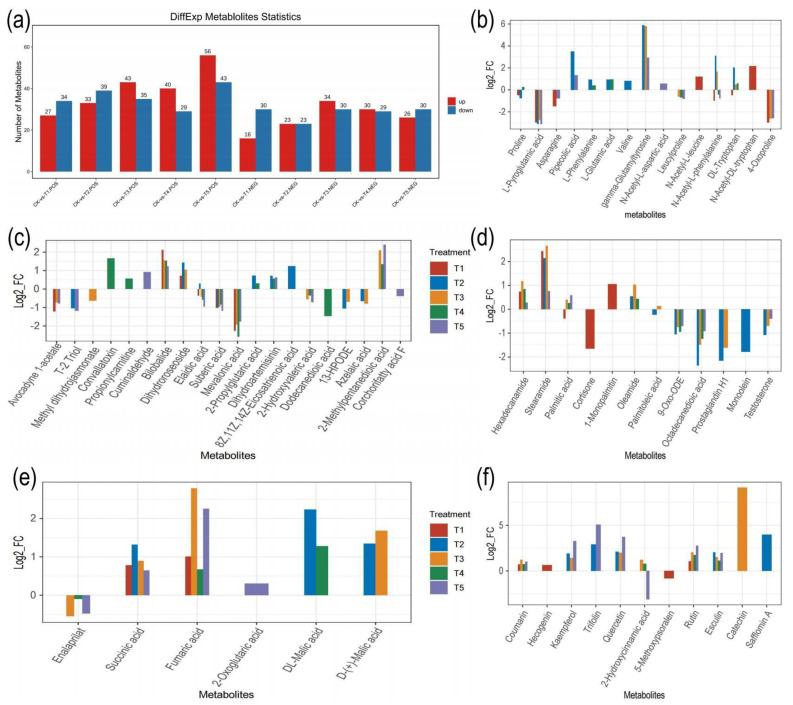
Bar plot of the differential metabolites from each group comparison. (**a**) Number of differential metabolites from the positive and negative ion modes; (**b**) number of differential amino acid metabolites; (**c**,**d**) number of differential lipid metabolites; (**e**) number of differential organic acid metabolites; (**f**) number of differential secondary metabolites. POS, positive; NEG, negative; Up, upregulated; Down, downregulated.

The differential expressed metabolites (DEMs) in CK and the T1–T5 samples from the LC-MS ion modes were screened to analyze the most significant biochemical changes. The DEMs were screened using the VIP (variable importance in projection) values with the criteria VIP ≥ 1 and *t*-test *p* < 0.05 in the OPLS-DA model. The results reveal that 379 (37.08%) of the 1022 ESI (+) metabolites, containing 199 up-regulated and 180 down-regulated, were DEMs between CK and T1-T5. In addition, 271 (37.07%) of the 731 metabolites in the ESI (−), including 129 up-regulated and 142 down-regulated, were DEMs in between CK and T1-T5 (Table S2). Furthermore, the DEMs between CK and each group of treatments were analyzed in the ESI (+) and ESI (−). Among them, CK-T1 and CK-T2 revealed a similar trend of DEM expression (Figure 6). In the CK-T1, 61 DEMs (27 up-regulated and 34 down-regulated) and 46 DEMs (16 up-regulated and 30 down-regulated) were in the ESI (+) and ESI (−), respectively (Figure 6a). CK-T2 revealed 72 DEMs (33 up-regulated and 39 down-regulated) in the ESI (+), whereas there were 46 DEMs, 23 up-regulated and 23 down-regulated, in the ESI (−) (Figure 6a). On the other hand, in a comparison of CK-T3, CK-T4, and CK-T5, the DEMs followed a similar expression pattern, with more DEMs expressed in the up-regulated pattern compared to the down-regulated patterns in both the ESI (+) and ESI (−) modes except for CK-T5 in the ESI (−) mode, for which the down-regulated pattern was slightly higher than the up-regulated pattern (Figure 6a). In a comparison of the treatment groups, the DEMs mainly included amino acids and derivatives (15), organic acids (6), secondary metabolites (11), lipids (32), organooxygen compounds, benzene and substituted derivatives; however, the majority of the DEMs were unclassified (Figure 6b–f, Table S2). Volcano plots were employed to show the relationships between the −log10 (*p*-value) and the fold change (log2(FC)) of all the identified DEMs within the comparison group in order to further understand the up- and downward regulations of the DEMs according to the VIP value and *p*-value < 0.05 (Figure S5). A cluster heat map analysis of the DEM clearly showed that samples in the same treatment clustered and were clearly distinguished between CK and the other treated groups (T1–T5), as shown in Figure S6. In addition, Figure S7 shows the relative contents of the metabolites in the individual comparisons.

**Figure 7:**
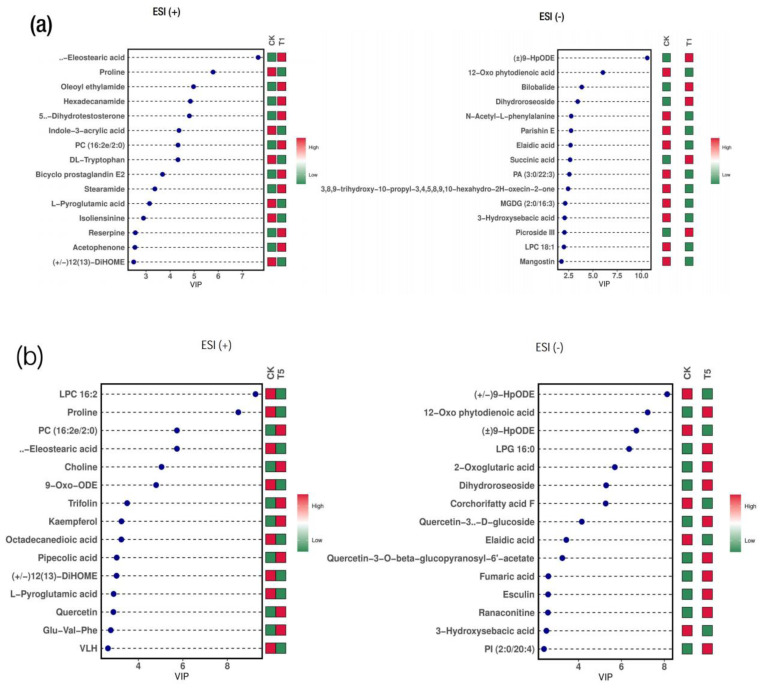
VIP plot of differential metabolites from Mg treatment in mulberry. (**a**) CK-T1; (**b**) CK-T5. The abscissa in the figure is the VIP value, the ordinate is the top 15 different metabolites for each group, the color on the right indicates the abundance of this metabolite in different groups, the red color is up-regulated, and the green color is down-regulated. The higher the VIP value, the greater the contribution to the differentiation of the sample, and the metabolites with a default VIP greater than 1 have a significant difference.

The influence of the DEMs was analyzed using the VIP values to determine the metabolites and their contributions to the sample differentiation. The results show that several DEMs had VIP values greater than 1 in the OPLS-DA. Figure 7 shows the top 15 most important metabolites (VIP ≥ 1; *p* < 0.05) that contributed to the sample differentiation within the group comparisons. These include amino acids (proline, L-pyroglutamic acid and N-acetyl-L-phenylalanine), organic acids (fumaric acid and succinic acid); lipids (dihydroroseoside, elaidic acid, hexadecanamide and stearamide); and those unclassified compounds (eleostearic acid, oleoyl ethylamide, hexadecanamide, (±)9-HpODE, parishin E and 12-Oxo phytodienoic acid) in the CK-T1 comparison (Figure 7a). For a comparison of low supplies of Mg (T2 and T3), the DEMs class within the top 15 VIP values included lipids (oleamide, palmitoleic acid, 9-Oxo-ODE, octadecanedioic acid, 2-propylglutaric acid, elaidic acid and dihydroroseoside,) amino acids, acids and derivatives (pipecolic acid, L-phenylalanine, proline and N-acetyl-L-phenylalanine), organic acids (DL-malic acid and succinic acid) and the unclassified (Indole-3-acrylic acid, eleostearic acid, linolenic acid, 12-oxo phytodienoic acid and (±)9-HpODE) (Figure S8a,b). In a comparison of the Mg oversupply group with the CK group, (T4 and T5), fatty acyls (oleamide, 9-Oxo-ODE, hexadecanamide, octadecanedioic acid, dihydroroseoside and elaidic acid), amino acids (proline, L-phenylalanine, L-glutamic acid and N-acetyl-L-phenylalanine) and the unclassified (±) 9-HpODE, LPS 18:2, LPC 18:1, parishin E, MGDG (2:0/16:3) and quercetin-3-O-beta-glucopyranosyl-6′-acetate), etc. (Figure 7b, Figure S8c), were among the top metabolites involved in the treatment responses.

### 2.5. Correlation and Interaction Networks Analysis between the Differential Metabolites’ Profiles

We analyzed the correlation map between the significant individual metabolites by calculating the Pearson correlation coefficient between all differential metabolites (MS2 levels). It was evident from the correlation heat map (Figure S9) that the obtained DEMs in all the treatment comparisons exhibited positive and negative correlation patterns (1 = r = −1). Furthermore, a metabolite network analysis reveals that several DEMs interacted strongly with other groups of compounds (Figure S10). For instance, in the CK-T1 group comparison, compounds such as fumaric acid, succinic acid, indole-3-acrylic acid, DL-tryptophan and others were strongly connected with other compounds (Figure S10a). In other instances, the comparison between CK and low-Mg treatments (T2, T3) also yielded some strong interactions amongst metabolites. For example, vindoline, oleamide, proline, T-2 triol, 9-oxo-ODE, DL-malic acid and azelaic acid were some compounds that interacted strongly with others (Figure S10b). A similar trend was observed in the comparison of CK with T4 and T5 (Figure S10c–e).

### 2.6. Metabolic KEGG Pathway and Enrichment Analysis

**Figure 8:**
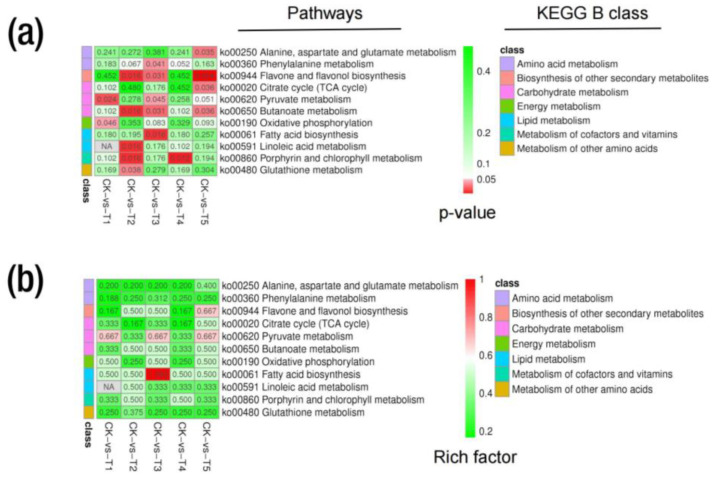
Differential metabolite KEGG analysis from various Mg treatments in mulberry. (**a**) Differential metabolite KEGG pathways based on *p*-values; (**b**) differential KEGG enrichment pathways based on enrichment factor. CK, control; T1, 0 mmol/L; T2, 1 mmol/L; T3, 2 mmol/L; T4, 6 mmol/L; T5, 9 mmol/L of Mg.

The metabolic pathway analysis indicated that all the 1753 metabolites from the ESI (+) and ESI (−) modes were annotated to 94 KEGG pathways. The annotation was grouped into a KEGG A class, B class, and pathways. The KEGG A class comprises metabolism, genetic information processing and environmental information processing (Figure S11a). In addition, the DEMs in all the treatment group comparisons were annotated to the same KEGG pattern (Figure S11b–f). In the CK-T1 group, the DEMs were annotated to 38 KEGG pathways. However, two pathways related to pyruvate metabolism in the carbohydrate metabolism and oxidative phosphorylation in the energy metabolism (KEGG B class) were significant based on their *p*-values (<0.05) and biological significance (Table S3). The relative abundances of metabolites such as fumaric acid and succinic acid were up-regulated in these pathways (Figure 8a, Table S3). For CK-T2, the DEMs were annotated to 49 KEGG pathways. Interestingly, five pathways related to linoleic acid metabolism, butanoate metabolism, porphyrin and chlorophyll metabolism, flavone and flavonol biosynthesis and glutathione metabolism were significant (Table S3). In the linoleic acid metabolism, 9-Oxo-ODE and 13-HPODE were down-regulated but 8Z,11Z,14Z-eicosatrienoic acid was up-regulated (Figure 8a). In the butanoate metabolism, L-glutamic acid, succinic acid and D-(+)-malic acid were up-regulated in this pathway (Figure 8a, Table S3). In the porphyrin and chlorophyll metabolism, L-glutamic acid and stercobilin were up- and down-regulated, respectively (Table S3). Kaempferol, quercetin and trifolin in the flavone and flavonol biosynthesis were up-regulated (Figure 8a, Table S3). L-glutamic acid and L-pyroglutamic acid were up- and down-regulated in the glutathione metabolism (Figure 8a, Table S3).

**Figure 9:**
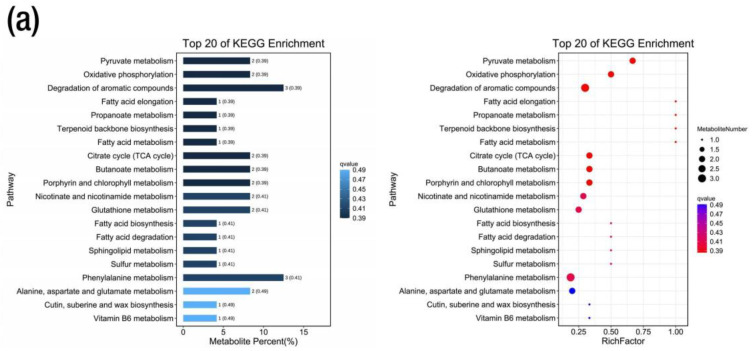

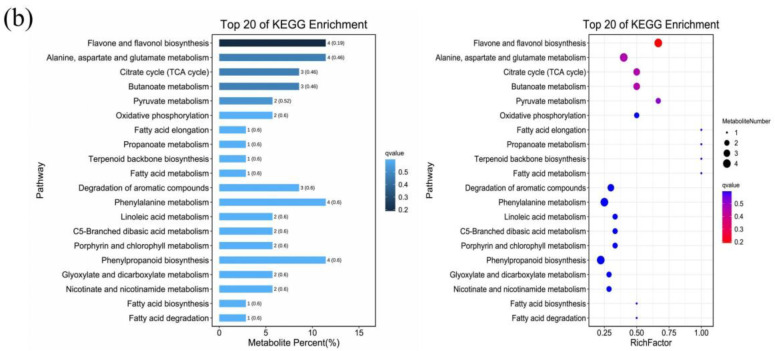
Differential metabolite KEGG enrichment analysis of the top 20 pathways from Mg treatments in mulberry. (**a**) Mg deficiency (CK-T1); (**b**) Mg toxicity (CK-T5). The bar graph represents the metabolites’ percentages in the pathways, and the bubble plot on the right side represents the enrichment factors of the metabolites in the pathways using the smallest q-values.

Furthermore, in the CK-T3 group, the DEMs were annotated to 43 KEGG pathways, with the most significant pathways related to fatty acid biosynthesis, butanoate metabolism, flavone and flavonol biosynthesis, phenylalanine metabolism and pyruvate metabolism (Figure 8a). Palmitic acid, palmitoleic acid, D-(+)-malic acid, fumaric acid, succinic acid, N-acetyl-L-phenylalanine, kaempferol, quercetin, rutin and 2-hydroxycinnamic acid were all involved in these pathways (Table S3). Remarkably, all the compounds involved in the pathways were all up-regulated. In the Mg toxicity groups, the DEMs were annotated to 100 pathways (49 in CK-T4 and 51 in CK-T5). However, only one pathway related to porphyrin and chlorophyll metabolism with L-glutamic acid and stercobilin metabolites was significant. Remarkably, five pathways were significant to CK-T5. These pathways included flavone and flavonol biosynthesis, butanoate metabolism, pyruvate metabolism, citrate cycle (TCA cycle), and alanine, aspartate, and glutamate metabolism. Metabolites such as 2-oxoglutaric acid and N-acetyl-L-aspartic acid were involved in these pathways (Figure 8a, Table S3). Other compounds in these pathways were the same as in the CK-T3. As shown in Figure 8b, the analysis based on the enrichment factor indicated that the DEMs were involved in amino acid metabolism, lipid metabolism, the biosynthesis of other secondary metabolites, carbohydrate metabolism, energy metabolism, etc. We further carried out a KEGG enrichment analysis on the DEMs using the top 20 metabolic pathways with the smallest q-values. The percentage(s) of metabolites and the top 20 enrichments, based on the rich factors in all the treatment groups, are shown in Figure 9 and Figure S12.

### 2.7. Correlation Analysis between the Differential Metabolites’ Profiles and Mulberry Physiological Parameters Measured

**Figure 10:**
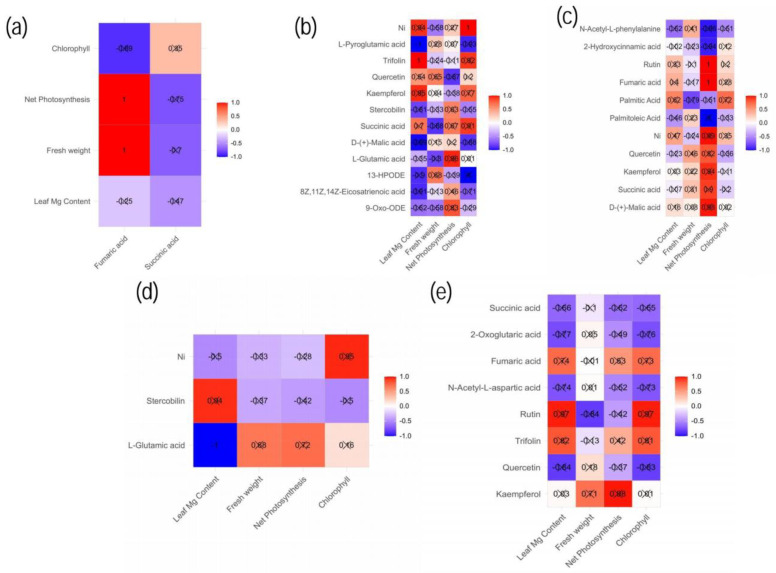
A correlation map of the significantly differential metabolites and physio-morphological parameters (chlorophyll content, leaf Mg content, P_n_ and Fresh weight) of *Morus alba* under conditions of magnesium deficiency (**a**), low Mg (**b**,**c**) and excess Mg (**d**,**e**). Each square indicates Pearson’s correlation coefficient values (r) for a pair of metabolites and the physio-morphological parameters. The red color indicates a positive (0 < r = 1) correlation, and the blue color indicates a negative (−1 = r < 0) correlation.

A correlation map analysis was conducted to visualize the correlations between the DEMs in the significant KEGG pathways and the physiological data in each group comparison. The correlation map (Figure 10) indicated a strong correlation between physiological parameters and some of the DEMs in the KEGG pathways. Organic acids such as fumaric acid correlated positively and strongly (r = 1) with fresh weight and P_n,_ while succinic acid correlated positively with chlorophyll in the Mg deficiency group (Figure 10a). Moreover, there was a negative correlation between chlorophyll and leaf Mg content, particularly with a strong negative correlation between chlorophyll and fumaric acid (r = −0.89), and same was seen with succinic acid.

For low Mg (T2), succinic acid, kaempferol, quercetin, trifolin and an unidentified compound named Ni showed a significant positive correlation with the leaf Mg content (Figure 10b). Moreover, there existed a strong negative correlation between the leaf Mg content and 8Z,11Z,14Z-eicosatrienoic acid, 13-HPODE, D-(+)-malic acid, 9-oxo-ODE and L-pyroglutamic acid (Figure 10b). Additionally, the total fresh weight showed a positive correlation with 13-HPODE, kaempferol, quercetin, L-pyroglutamic acid and D- (+)-malic acid, although the correlations of L-pyroglutamic acid, D-(+)-malic acid and kaempferol were not statistically significant. Positive correlations between P_n_ and several compounds were identified, including 9-oxo-ODE, 8Z,11Z,14Z-eicosatrienoic acid, L-glutamic acid, succinic acid, stercobilin, D- (+)-malic acid and L-pyroglutamic acid, but net photosynthesis and quercetin were negatively correlated. Furthermore, chlorophyll was found to be strongly and positively correlated with succinic acid, kaempferol, trifolin and Ni. On the contrary, L-pyroglutamic acid, D-(+)-malic acid and 13-HPODE were negatively correlated with chlorophyll (Figure 10b). In the Mg supply (T3), the leaf Mg content was positively correlated with palmitic acid, fumaric acid, rutin and Ni; however, palmitoleic acid and N-acetyl-L-phenylalanine were among the negatively correlated compounds (Figure 10c). However, palmitic acid exhibited a strong negative correlation with the fresh weight. Among the compounds that displayed strong positive correlations with P_n_ were rutin, fumaric acid and succinic acid, with correlation coefficients of 1 and 0.9, respectively. Palmitoleic acid, N-acetyl-L-phenylalanine and 2-hydroxycinnamic acid exhibited strong negative correlations with P_n_. For an excess Mg supply (T4), stercobilin correlated strongly and positively with leaf Mg content but correlated negatively with fresh weight, P_n_ and chlorophyll. In addition, L-glutamic acid exhibited a strong negative correlation with leaf Mg content. However, a positive correlation was observed between the fresh weight, P_n_ and chlorophyll content. With excessive Mg application (T5), the content of Mg in the leaves exhibited strong positive correlations with rutin, trifolin and fumaric acid (Figure 10e). Interestingly, succinic acid, 2-oxoglutaric acid and N-acetyl-L-aspartic acid showed negative correlations. Conversely, the fresh weight showed strong positive correlations with kaempferol, with rutin displaying the opposite trend. Additionally, P_n_ exhibited strong positive correlations with kaempferol (r = 0.98), trifolin and fumaric acid. Chlorophyll exhibited strong positive correlations with trifolin, rutin and fumaric acid, but quercetin, succinic acid and 2-oxoglutaric acid showed negative correlations.

## 3. Discussion

Mg plays multiple roles in biological systems [20]. Therefore, understanding plant responses to Mg deficiency and excess is crucial for the better management of plant nutrients in plant production. Information regarding Mg applications in mulberry in relation to metabolic and phenotypic characteristics is rare. Thus far, to the best of our knowledge, a single article assessing Mg deficiency in mulberry (*M. alba*) [17] has been produced. Based on LC-MS untargeted metabolomics, the present study provides a metabolomics profile of mulberry leaves in response to Mg deficiency and low or excess levels of Mg and the corresponding physiological parameters. The present study indicates that the growth of *M. alba* seedlings was affected when exposed to Mg deficiency and Mg supply (low and excess) compared to the control of sufficient Mg.

### 3.1. Physiological Responses to Mg Supply and Deficiency in M. alba

Owing to the measured physiological parameters, this research revealed that deficient, low and excess Mg supplies negatively interfered with biomass accumulation (Figure 1), photosynthetic pigments (Figure 2) and gas exchange (Figure 3) and were intrinsically related to a low Mg content and stomatal closure. However, the control (CK) showed improved stomatal (density) opening (Figure 4), photochemical efficiency (Figure 2) and gas exchange (Figure 3). Additionally, plants exposed to CK samples showed markedly increased Mg contents in both roots and leaves, suggesting an improvement in the absorption, transportation and accumulation of Mg in the tissues evaluated in mulberry. The current research results agree with those reported [21], which reported similar trends in soybean plants supplied with Mg and sprayed with 24-epibrasinolide. In this present study, Mg deficiency and excess affected CO_2_ assimilation, causing reductions in the biomass of mulberry plants by 55% (T1) and 58% (T5) relative to the CK group (Figure 1). Our results agree with a recent study including a systemic review and meta-analysis which suggested that an optimum Mg application improves net CO_2_ assimilation by 140%, translating into an increase of 61% in biomass compared to Mg-deficient plants [22]. Recent advances in Mg research have reported that Mg deficiency influences the growth of aboveground parts more than roots [23]. This implies that the reduced Mg content of leaves relative to roots observed in this study indicates that leaf biomass was negatively affected (Figure 1), suggesting that Mg deficiency not only interfered with the movement, localization and distribution of photoassimilates into the phloem but also impeded the downward movement of photosynthates from the leaves to the roots [22,23].

In chlorophyll molecules, Mg occupies the central position of the porphyrin ring and activates or regulates several kinases, such as ATPases and ribulose-1,5-bisphosphate carboxylase/oxygenase (RuBisCO) [21,24,25]. Consequently, Mg deficiency is detrimental to numerous physiological processes such as chlorophyll biosynthesis, plant growth and plant development under Mg stress [25]. Mulberry plants experiencing Mg deficiency and excess Mg suffered marked reductions in photosynthetic pigments (Chl *a*, Chl *b* and Chl *a*+*b*) (Figure 2). Relative to the control (CK), there were significant reductions in chlorophyll *b* and *a*+*b* in deficient and excess Mg treatments, which could be observed in T1 (27 and 28%) and T5 (23 and 24%), respectively; however, the effect was not pronounced in Chl *a* (22%). This phenomenon explains the adverse ramifications of Mg deficiency and excess on the chlorophyll pigmentation in mulberry plants. The decrease in photosynthetic pigments could be attributed to the lower levels of Mg in the leaves of Mg-deficient and toxic plants, which could have inhibited the biosynthesis of chlorophyll in mulberry. This development was confirmed by Li et al. (2017), who observed no significant variations in Chl *a* in the upper leaves of *Citrus sinensis* supplied with sufficient and deficient Mg conditions compared with the lower leaves [26]. Meanwhile, the responses of Chl *a* and *b* to Mg have been found to exhibit differential, non-uniform, cultivar-specific responses and sometimes do not correlate with Mg-induced deficiency [27].

Plants reduce water loss by adjusting the degree of opening of their stomatal pores according to atmospheric conditions, a process which is directly linked to the short-term regulation of Gs [28]. Changes in Gs caused by anatomical adaptations can alter the range of Gs due to genetics and/or environmental factors [28]. Compared to other treatments, the increase in stomatal density in the CK group in this present study (Figure 1e) suggests that an optimum level of Mg improved the stomatal opening. Thus, a higher concentration of CO_2_ may be captured and made available for photosynthetic processes. This was reflected in a higher biomass, gas exchange and pigments under control. The result is in accordance with what was opined in soybean plants grown with a Mg supply and supplemented with 24-epibrassinolide [21,29]. Therefore, it is quite plausible that the regulation and density of stomatal openings via Mg supply ensures the capture of CO_2_ to maximize photosynthetic activity and reduce transpiration.

### 3.2. Differential Metabolites in M. alba Leaves: Response to Various Mg Treatments and Deficiency

Metabolomics programming in plants’ responses to Mg supply was investigated. We visualized the metabolites’ profiles from all the treated samples to investigate the potential events occurring during the Mg deficiency, sufficiency and low or excess Mg conditions in this study. Based on the OPLS-DA plots, all the treatment groups observed a clear separation of metabolites (Figure 5 and Figure S3). The abundances of the altered DEMs indicate the most affected class of metabolites in response to Mg deficiency or low or excess Mg, including amino acids and organic acids, fatty acyl, prenol lipids, flavonoids and the unclassified metabolite [6,9] (Table S2).

### 3.3. Mulberry Plants’ Response to Mg Supply and Deficiency Induces Amino Acids and Organic Acids

Amino acids are the fundamental constituents of proteins and are crucial for the synthesis of various biological pathways, intracellular communication mechanisms and the adaptive response to external stressors [30]. In the present study, several amino acids and their derivatives were accumulated under conditions of Mg deficiency (T1), treatment with low levels of Mg (T2 and T3) or Mg toxicity (T4 and T5) (Figure 6b; Table S2). Among these amino acids were proline, L-pyroglutamic acid, asparagine, pipecolic acid, valine, L-phenylalanine, L-glutamic acid and derivatives such as gamma-glutamyltyrosine, N-acetyl-L-leucine, N-acetyl-L-phenylalanine, etc. The accumulation of amino acids in response to Mg deficiency has been reported extensively [9,31]. According to Yang et al. (2017), a greater build-up of amino acids was seen in Mg-deficient soybean leaves [31]. Our study reveals that even though amino acids accumulated, the relative abundance decreased in mostly the Mg-deficient and Mg toxicity groups, which agrees with other studies reported [32,33]. For instance, the relative abundances of proline, asparagine and L-pyroglutamic acid were significantly decreased in Mg-deficient plants (T1), plants with low Mg treatment (T2 and T3) or plants with Mg toxicity (T5) (Table S2). Mg deficiency has been reported to inhibit nitrogen metabolism in spinach leaves, leading to amino acid build-up [33]. The build-up of amino acids may be connected to the suppression of protein synthesis, resulting in a decreased amino acid intake in Mg-deficient leaves. Proline (Pro) is a crucial amino acid, functioning as a compatible solute and contributing significantly to plant stress tolerance [34].

Mg deficiency can disrupt nitrogen assimilation and decrease the availability of nitrogen for Pro synthesis, leading to decreased Pro accumulation. This is because Pro and nitrogen metabolism share similar metabolic pathways [35]. Therefore, a decreased level of Pro in the Mg-deficient plants could suggest the disruption of nitrogen assimilation. Th content of asparagine (Asn) has been reported to increase in Mg-deficient leaves of soybean [31]. Mulberry leaves exhibited the opposite in this study, as the content of Asn decreased in both the Mg-deficient and Mg toxicity groups. Nitrogen metabolism is significantly dependent on Asn, an amino acid that functions as a nitrogen storage and transport molecule in plants [36]. Mg deficiency and toxicity both have detrimental effects on the synthesis of asparagine in plants. Mg deficiency restricts protein synthesis due to the decrease in enzyme activity, which relies on Mg for protein synthesis, thus inhibiting the activity of asparagine synthetase. In contrast, Mg toxicity impedes nitrogen metabolism, thereby diminishing the access to nitrogen for asparagine synthesis [31,36,37]. In a recent study, it was reported that Mg oversupply resulted in the down-regulation of the content of free amino acids [20], a result which agrees with our study. The content of L-pyroglutamic acid in Mg-deficient and Mg toxicity mulberry plants decreased. The relationship between Mg treatment and L-pyroglutamic acid is not well documented, and further research is needed.

Metabolites such as pipecolic acid, valine, DL-tryptophan, L-phenylalanine, L-glutamic acid and proline were increased in T2 and T4. Glutamic acid is an essential amino acid for plant metabolism as it is needed for the synthesis of proteins and is a precursor for the manufacture of other amino acids that require Mg supply and is involved in the transport of nitrogen within the plant [38] The increases in L-glutamic acid in T2 and T4 could suggest the synthesis of other amino acids. A previous study reported that phenylalanine and glutamic acid decreased in response to an oversupply of Mg [20]. Increasing valine, L-phenylalanine and L-glutamic acid amino acids in response to low and high Mg supplies suggest how important Mg supply is, as it corresponds to the synthesis of essential amino acids. The relatively higher concentrations of L-phenylalanine and N-acetyl-L-phenylalanine could suggest the upregulation of the shikimate pathway in the mulberry leaves following Mg supply [6,9]. Variations in the amounts of primary metabolites indicate changing regulatory mechanisms in plant cells [39].

Organic acids are typically recognized as intermediates of photosynthesis and substrates of respiration in higher plants [40]. They are predominantly generated in plants due to the incomplete oxidation of photosynthetic products, and they reflect the pools of fixed carbon build-up due to the variable conversion periods of carbon molecules in metabolic pathways [40]. Mg is a cofactor in activating organic acid synthases, including citrate synthase, malate synthase and pyruvate kinase [41]. In response to Mg deficiency, plants initiate a range of metabolic processes to manage the deficiency, including the accumulation of organic acids such as citric acid, malic acid and oxalic acid, which are essential for Mg uptake and transport within plants. An abundance of Mg can cause a surge in organic acid concentrations within plants due to an ion imbalance. Therefore, the roots of the plants excrete organic acids into the rhizosphere in order to expel the excessive Mg ions and restore the nutrient ratios in the cells to an optimal level. In the present study, the abundances of organic acids such as fumaric acid, succinic acid, 2-oxoglutaric acid, malic acid and trans-aconitic acid increased in Mg-deficient mulberry leaves and mulberry leaves with low or high supplies of Mg compared to the control. Mg shortage has been shown to affect the metabolism of organic acids by decreasing some organic acids, such as malate and citrate [42]. 

A recent study observed downregulated abundances of organic acids in Mg-deficient *Citrus. sinensis* leaves [9] and Mg oversupply in tomato plants [20]. In contrast, we observed upregulated contents of most organic acids in Mg-deficient mulberry leaves or mulberry leaves with low or high supplies of Mg, whereas enalaprilat decreased in abundance in the leaves with Mg toxicity and low supply and was ever expressed in Mg-deficient leaves. The increases in the contents of fumaric and succinic acids could suggest their roles in the fixation of CO_2_ and the redox balance in the different cell compartments, thus mitigating the effect of Mg deficiency or a low supply or oversupply of Mg. Our study agrees with the results of Yang et al. (2017), who reported an increase in organic acid abundance in soybean leaves [31]. Additionally, 2-oxoglutaric acid (2-OG) and succinic acid are regarded as alternative downstream products of amino acids from protein degradation to enter respiration [43,44]. Under the conditions of Mg toxicity in plants, 2-oxoglutaric acid concentration increased [31], indicating why we may have seen the expression and up-regulation of 2-OG and upregulated only in the Mg toxicity group in this study. The reason for the up-regulation of 2-OG in the Mg toxicity plants could be attributed to Krebs Cycle interference; 2-OG is an intermediate compound in the Krebs cycle (the citric acid cycle or tricarboxylic acid cycle), which is a crucial metabolic pathway for energy production in plants [44]. Mg is an essential cofactor for numerous enzymes that are involved in the Krebs cycle; however, an excess of Mg can lead to the disruption of enzyme activities, thus resulting in an increase in 2-OG due to the altered metabolic flux. Plants activate defense mechanisms, including the production of 2-OG, which is involved in the regulation of antioxidant enzymes such as glutathione peroxidase and catalase, which help to reduce the effects of oxidative stress. Therefore, the increase in 2-OG under the conditions of Mg toxicity can be attributed to the plant’s attempt to mitigate oxidative stress [31,43,44].

### 3.4. Lipid Metabolites’ Reponses to Mg Supply and Deficiency in Mulberry Plants

Lipids are energy storage molecules that act as membrane components and in cellular structure formation and signaling [45], and a decrease in plant lipid molecules could result in the impairment of plant growth. Interestingly, a high accumulation of lipid levels in mulberry under stress has been reported [1]. Remarkably, lipid accumulation was prevalent in this study (Figure 6c,d). An increase in lipid abundance in relation to Mg deficiency has been reported [9]. Interestingly, we found that Mg deficiency did not influence the expression of many lipids (10); however, the content of lipids including avocadeyne 1-acetate, mevalonic acid, suberic acid, elaidic acid and octadecanedioic acid decreased under conditions of mulberry Mg deficiency. Nonetheless, lipids such as bilobalide, hexadecanamide, stearamide, 1-monopalmitin and dihydroroseoside had their abundances increased. When experiencing Mg deprivation, plants are likely to accumulate lipids due to a decrease in the activity of the enzymes involved in lipid degradation and a change in carbon metabolism. Mg is a vital cofactor for many enzymes that are involved in lipid metabolism, for instance, phospholipase D, which breaks down phospholipids to create free fatty acids. Therefore, the lack of Mg can reduce the activity of this enzyme, causing an increase in the number of phospholipids and other lipids in the plant’s cells.

An excessive supply of Mg has been argued to cause plant stress as it turns out to be toxic to plant cellular components, thus affecting plant productivity [46]. For instance, in serpentine soil, Mg toxicity usually occurs when plants are exposed to low-calcium (Ca) and high-Mg conditions, leading to poor plant productivity [46]. In this study, several of the lipid accumulations occurred under conditions of a low or excessive Mg supply (Figure 6c,d). Most of these lipids (palmitic acid, stearamide, 2-propyglutaric acid, oleamide, palmitoleic acid, etc.) were increased in abundance in either low-Mg conditions or during Mg toxicity [20]. On the other hand, several which were low in abundance responded to Mg toxicity in mulberry plants (Figure 6c,d, Table S2). An overabundance of Mg can interfere with the balance of essential cations such as Ca and K, thus influencing the synthesis and accumulation of lipids. Additionally, Mg toxicity can also induce oxidative stress, which generates ROS in plants, leading to membrane damage and modifications in lipid composition. According to [6], Mg oversupply decreases triterpenoids contents. However, we found an upstream regulation of prenol lipids (cuminaldehyde, bilobalide and dihydroartemisinin). A significant accumulation of lipid metabolites suggests that there was memory disruption due to imbalances om the Mg supply, which might have affected the activities of many enzymes.

### 3.5. Mulberry Plants Induce Secondary Metabolites’ Response to Mg Deficiency and Supply

Secondary metabolites are important compounds needed by plants in critical condition and therefore are not directly involved in the primary activities of plants. Studies reveal that MgSO_4_ application in snap beans increases their total flavonoid content [47]. Flavonoids are an important group of secondary metabolites. The present study reveals that secondary metabolites, mainly flavonoids, cinnamic acids and coumarins, and their derivatives responded to Mg deficiency and low or high supplies of Mg (Figure 6f). Flavonoids are secondary metabolites that function as significant antioxidants, and their accumulation in plants lowers the direct or indirect oxidative damage induced by abiotic circumstances [48]. According to Ahmad et al. (2021), Mg deficiency enhanced the accumulation of secondary metabolites, including total phenolic contents and total flavonoid contents, in the plant biomass of *Stevia rebaudiana* [49]. It has been opined that an oversupply of Mg negatively affects flavonoids and cinnamic acid content [6]. In our results, we observed, however, that the contents of flavonoids (catechin, rutin, quercetin, kaempferol and trifolin) were highly increased in groups with Mg deficiency or low- or high-Mg-treated groups [9]. A similar trend was found in the cinnamic acids and coumarins and their derivatives (2-hydroxycinnamic acid, safflomin A, esculin and coumarin); however, 2-hydroxycinnamic acid content decreased under Mg oversupply conditions. The higher levels of flavonoids under the conditions of Mg imbalance identified in this study could be attributed to their antioxidant capacities to act as defense moieties to overcome stress challenges. The cinnamic molecule, another essential phenylpropanoid compound, was greatly changed, which may suggest its significance in controlling the flux into the phenylpropanoid pathway [50], and their build-up under the supply of Mg suggests their antioxidant action against ROS. Our study also agrees with a study on tomatoes under conditions of Mg oversupply [20], which reported a marginal increase in flavonoids’ responses to excessive Mg application. Stilbenes are important secondary metabolites, and they have been reported to accumulate in mulberry under abiotic stress [1]. Interestingly, in this study, we found only isorhapontigenin (a stilbene), and it was up-regulated only in low- and high-Mg-supply plants, suggesting that the mulberry’s response to an Mg imbalance did not induce stilbene metabolites.

### 3.6. Correlations between the Differential Metabolite Profiles and Mulberry Physiological Parameters

Metabolite profiles are related to plant physiology, and their expression levels may have direct impacts on the phenotypic characteristics of plants [6]. Therefore, we performed a correlation analysis of the important metabolites involved in the significant KEGG pathway metabolism. We observed that several compounds were positively correlated with some important physiological parameters associated with Mg supply and deficiency (Figure 10). Most importantly, we observed that amino acids, organic acids, lipids and secondary metabolites were the most crucial metabolites involved in the significant pathways that correlated with the physiological parameters, suggesting that these classes of compounds may play critical roles in mulberry growth and development under Mg imbalances.

Organic acids in higher plants are known to act as intermediates of photosynthesis and as substrates of respiration [51]. Fumaric acid was found to correlate positively (r = 1) with P_n_, and plant biomass and succinic acid positively correlated with chlorophyll but negatively correlated with P_n_ and plant biomass in the Mg-deficient plants (Figure 10a). A recent study reported that succinic acid (SA) treatment in *Microcystis aeruginosa* resulted in damage to the oxygen-evolving complex (OEC) and reaction center of PSII, thus, blocking photosynthetic electron transport and thereby lowering the rate of photosynthesis and inhibiting the growth of *Microcystis* [52]. Moreover, malic acid, succinic and fumaric acids were observed to correlate positively (strongly) with P_n_ and chlorophyll and, to some extent, leaf Mg content and biomass when Mg was applied in either low or excess doses (Figure 10b–d). During the night, CO_2_ is taken in and incorporated into the carboxyl group of organic acids, mainly malic acid, which is stored in the cell vacuole where it is formed. During the day, the stored organic acid is decarboxylated to produce CO_2_, which is then used in the photosynthetic carbon reduction cycle [51]. The role of malic acid as an intermediary in CO_2_ fixation has been extensively studied in terms of stomatal opening and water use efficiency [51]. Therefore, is not a coincidence that many of the organic acids correlated with photosynthesis machinery under Mg supply since Mg acts as a cofactor to activate most of the enzymes involving these compounds. Surprisingly 2-oxaloglutaric acid, an important organic acid which functions as a key metabolite in the TCA cycle, correlated negatively with the physiological data measured.

Flavonoids are known to have multiple roles in plants, such as providing protection from oxidative stress, UV radiation, and herbivory [53]. Additionally, research has indicated that flavonoids may have an effect on the photosynthetic process in plants [54]. In the Mg-treated samples, whether with low or excess Mg supply, most of the secondary metabolites involved in the significant pathways were flavonoids. Most of the flavonoids (quercetin, kaempferol, rutin and trifolin) correlated positively with measures of the physiological data (P_n,_ chlorophyll and, to some extent, leaf Mg content and biomass) in either low or high Mg supplies [54]. Flavonoids’ role in photoprotection, which is afforded by the high quercetin/kaempferol ratio, has been reported in *Petunia* leaves [55]. As antioxidants, flavonoids can protect chloroplasts and other photosynthetic organelles from oxidative stress due to high light intensities or other environmental stressors, consequently augmenting the efficacy of photosynthesis and increasing yield. This phenomenon becomes effective under Mg supply since Mg acts as a cofactor in activating important enzymatic pathways [27]. Amino acids such as glutamic acid correlated well with the photosynthesis machinery, including P_n,_ chlorophyll and biomass, under low and excess Mg supplies.

## 4. Materials and Methods

### 4.1. Source of Mulberry Plants and Growth Conditions

*M. alba* (Yu-711) was retrieved from the National Mulberry GenBank at Jiangsu University of Science and Technology in Zhenjiang, Jiangsu, China. Below is a description of the plant growth, conformed to [56]. Briefly, mulberry grafted seedlings were selected and planted in 35 cm diameter pots containing loamy soil and vermiculite. The greenhouse conditions for the plants’ growth were as follows: 14 h of light, 10 h of dark, 25 °C during the day and 20 °C at night and from 70 to 80% humidity. In total, there were 18 pots, with each pot containing three plant seedlings as replicates. The seedlings were watered daily and supplemented with an MS culture medium solution containing 4.37 g MS media dissolved in 1000 mL (pH = 7.0) every three days for 7 days. The plants were treated with deionized water for 7 days after the leaves were fully grown. Mg (MgSO_4_) treatment followed, according to work by [17,57] with some modifications. A total of six concentration gradients, 0 mmol/L (T1) as Mg deficiency, 1 mmol/L and 2 mmol/L (T2 and T3) as low Mg, 3 mmol/L as sufficiency (CK), and 6 mmol/L and 9 mmol/L (T4 and T5) as Mg excess, were applied to the mulberry plants for 20 days. Leaf samples were then collected on the 20th day and stored at −80 °C for further analysis. Leaves showing Mg deficiency were also observed and collected for further studies.

### 4.2. Mineral Analysis

Mg contents in the mulberry leaves and roots were determined using an ICP-AES/OES/MS (PerkinElmer Waltham, MA, USA) instrument, following the National food safety standard determination of multiple elements in food (GB 5009.268-2016). Briefly, 0.2–1.0 g of a dry sample or 1.0–2.0 g of a fresh sample (accurate to 0.0001 g) was weighed in the inner tank of PTFE digestion. Then, 5 mL of nitric acid was added and soaked overnight. Next, the inner was covered, and the stainless-steel jacket was tightened and placed in a constant-temperature drying oven, kept at 80 °C for 1–2 h and 120 °C for 1–2 h, and then raised to 160 °C for 4 h. After cooling to room temperature, the acid was heated to nearly dry after opening, and the digestion solution was washed and placed in a 25 mL volumetric flask. Next, the inner tank and inner lid were washed three times with small amounts of nitric acid solution (1%); the washing solution was combined and placed into the volumetric flask, and 1% nitric acid was fixed to the scale, mixed well and set aside. At the same time, a blank reagent test was prepared. The test solution was then used to determine the Mg content.

### 4.3. Physiological Parameters Measurement

Using the PPSYSTEMS CIRAS-3 portable photosynthetic instrument (PP systems, MA, USA) mulberry leaves (3–5) with the same leaf position and growth were selected and used to determine the net photosynthetic rate, transpiration rate, stomatal conductivity and intercellular carbon dioxide concentration between 9:00 and 11:00 a.m. (with sunny and cloudless conditions). The light intensity was 1200 μmoL·m^−2^ s^−1^, the leaf temperature was about 25 °C, the relative humidity was 70~80%, and the atmospheric CO_2_ concentrations were stable at 380~420 μmol·mol^−1^. In addition, a field emission scanning electron microscope (QUANTA 250 FEG, Thermo Fisher Scientific, Sunnyvale, CA, USA) was used to analyze the stomatal opening of the leaf samples in each treatment group. The mulberry biomass was determined by first placing a glass petri dish on an electronic balance and zeroing it; then, samples of leaves from the various treatments were taken and placed on an electronic balance, and the mass (i.e., fresh weight) was weighed three times, using three leaves with the same conditions as the replicates. A total of six concentration gradients and 18 samples were used. Finally, 18 samples were placed in an oven (65 °C), and the leaves were dried to determine the dry weight. The same was repeated three times, as with the fresh weight.

### 4.4. Determination of Chlorophyll Content

The chlorophyll content was determined using a Plant Chlorophyll Content Kit (Trace Method) (Keming Biotechnology Co., Ltd., Suzhou, China), following the manufacturer’s instructions. Briefly, fresh mulberry leaves from the treatment samples were weighed, and the veins were removed. About 0.1 g of the leaf was weighed and used for chlorophyll content determination according to the company’s kit.

### 4.5. Sample Preparation for Metabolite Extraction

All chemicals and solvents used were analytical or HPLC grade. Water, methanol, acetonitrile and formic acid were purchased from CNW Technologies GmbH (Düssel-dorf, Germany). Using liquid nitrogen, mulberry leaf tissues (100 mg) were separately ground, and the homogenate was resuspended with 500 µL prechilled 80% methanol and vortexed to mix. After a brief (5 min) chilling on ice, the samples were centrifuged at 15,000× *g* at 4 °C for 20 min. LC-MS-grade water was used to dilute the supernatants to a final concentration of 53% methanol. The samples were then centrifuged in a new Eppendorf tube at 15,000× *g* at 4 °C for 20 min. The resulting supernatant was then analyzed with an LC-MS/MS equipment [58].

### 4.6. UHPLC-MS/MS Analysis

UHPLC-MS/MS analyses were performed using a Vanquish UHPLC system (ThermoFisher, Düsseldorf, Germany) coupled with an Orbitrap Q ExactiveTM HF-X mass spectrometer (Thermo Fisher, Germany) in Gene Denovo Co., Ltd. (Guangzhou, China). At a flow rate of 0.2 mL/min, samples were injected onto a Hypesil Gold column (100 × 2.1 mm, 1.9 µm) using a 17-minute linear gradient. The positive polarity mode eluents were eluent A (0.1% FA in Water) and eluent B (methanol). The eluents for the negative polarity mode were eluent A (5 mM ammonium acetate, pH 9.0) and eluent B (methanol). The solvent gradient was set as follows: 2% B, 1.5 min; 2–100% B, 12.0 min; 100% B, 14.0 min; 100–2% B, 14.1 min; 2% B, 17 min. A Q ExactiveTMHF-X mass spectrometer was operated in positive/negative polarity mode with a spray voltage of 3.2 kV, a capillary temperature of 320 °C, a sheath gas flow rate of 40 arb and aux gas flow rate of 10 arb.

### 4.7. Data Processing and Metabolite Identification

A Compound Discoverer 3.1 (CD3.1, Thermo Fisher, USA) was used to perform peak alignment, peak selection, and quantification for each metabolite from the raw data files obtained via UHPLC-MS/MS. The main parameters were set as follows: retention time tolerance, 0.2 min; actual mass tolerance, 5 ppm; signal intensity tolerance, 30%; signal/noise ratio, 3; and minimum intensity, 100,000. The peak intensities were then normalized to the overall spectral intensity. The normalized data were utilized to determine the molecular formula based on additive ions, molecular ion peaks and fragment ions. Peaks were then matched with the mzCloud (https://www.mzcloud.org/ (accessed on 10 December 2022)) and mz Vault and Mass Listdatabases to provide precise qualitative and relative quantitative results. R (version R-3.4.3), Python (v 2.7.6) and CentOS were utilized for statistical analysis (CentOS release 6.6).

### 4.8. Data Quality Control

A positive ion mode (POS) and a negative ion mode (NEG) were utilized to identify metabolites, resulting in greater metabolite coverage and enhanced detection effect. The positive and negative ion models were examined individually in the succeeding data analysis. In addition, QC samples were employed for quality control examinations [59].

### 4.9. Hierarchical Cluster Analysis

The data were subjected to a hierarchical cluster analysis (HCA), and the resulting dendrogram was generated using the average linkage method. Heatmap cluster analysis data were standardized with z-score and clustered hierarchically using the R tool pheatmap [60]. Correlation analyses of the replicates’ variability in the metabolite composition and the abundance between samples were quantified using correlation data between samples. A correlation analysis of duplicates assessed the variability in metabolite composition and abundance between samples. R was used for the correlation analyses, and the heatmap was created using the R package pheatmap.

### 4.10. PCA and OPLS-DA Analysis

An unsupervised dimensionality reduction approach principal component analysis (PCA) was applied to all samples using the R package models (http://www.r-project.org/ (accessed on 5 January 2022)) to visualize variations across sample groups [19]. PLS-DA was applied in comparison groups using R package ropls (http://www.rproject.org/ (accessed on 5 January 2022)) [61]. Orthogonal projection to latent structures discriminant analysis (OPLS-DA) [62] was applied in comparison groups using R package models (http://www.r-project.org/). The OPLS-DA model was further validated via cross-validation and a permutation test [63]. For cross-validation, the data were divided into seven subsets, with each subset serving as a validation set. R2 represented the overall variance in the data matrix that the model explained. Predictive ability (Q2) values are the most widely accepted diagnostic statistical criterion for validating the OPLS-DA model in the field of metabolomics. For Q2 values more than 0.4, an acceptable predictive model was deemed acceptable, and for Q2 values greater than 0.9, a good predictive model was deemed acceptable. The permutation test permutes class labels 200 times at random and generates a distribution of R2 and Q2 values.

### 4.11. Differential Metabolite Identification and KEGG Annotations and Enrichment Analysis

A variable importance in the projection (VIP) score of the (O)PLS model was used to rank the metabolites that discriminated between two groups effectively. The VIP threshold was set to 1. The t-test was also performed as a univariate analysis to check for distinct metabolites. Those with *p*-values for the *t*-test < 0.05 and VIP ≥ 1 were considered to differentiate between two groups. In order to comprehend the influence of different metabolites, the fold changes of abundance between two groups were calculated, and a volcano plot was generated. Using the z-score, the abundance of different metabolites within the same group was standardized. The graph was then constructed using the VIP score of OPLS-DA. The Pearson correlation coefficient and *p*-value were obtained with the cor. and cor.test R functions. The greater the closeness between metabolic abundances, the closer the correlation coefficient is to 1. When *p* ≤ 0.05, correlations were deemed significant [64]. Moreover, a correlation heatmap was drawn by the R corrplot package [65]. The abundances of differential metabolites were normalized via the z-score and hierarchically clustered by the R package pheatmap to show the accumulation differences between two groups. The z-score (standard fraction) is based on the relative content conversion of metabolites and is used to measure the relative content of metabolites at the same level. The calculating formula is as follows: z = (x − μ)/σ. Here, x is the abundance of a metabolite, μ is the mean of the abundance of a metabolite in every sample, and σ is the standard deviation [66]. The Kyoto Encyclopedia of Genes and Genomes (KEGG) [67] is the major public pathway-related database that includes both genes and metabolites. Metabolites were mapped to KEGG metabolic pathways for annotation and an enrichment analysis.

## 5. Conclusions

In general, several physiologically measured traits testify to the fact that Mg deficiency and excess and low supplies altered the photosynthetic apparatus, including P_n_, chlorophyll *b*, leaf Mg content and fresh weight, causing significant decreases in the photosynthetic efficiency and biomass of mulberry plants. Our study results demonstrate that the availability of an adequate amount of the Mg nutrient fosters the response of the mulberry plant’s physiological parameters, such as P_n_, chlorophyll and Mg content in both leaves and roots and biomass.

In addition, a scanning electron microscope (SEM) analysis revealed that Mg deficiency and excessive application adversely affected the stomatal opening of mulberry compared to the control. The metabolomics data show that different Mg concentrations affected several differential metabolite expressions (DEMs), particularly lipids, flavonoids, amino acids, organic acids and secondary metabolites (flavonoids, coumarins and cinnamic acids and derivatives). Thus, an excessive Mg supply produced more DEMs but negatively affected biomass production compared to a low supply and the optimum supply. This study reveals that despite the abundance of accumulated DEMs in the response of mulberry to Mg imbalances in comparison with an adequate Mg supply, the majority were unclassified, suggesting that several new compounds were induced in the mulberry plant response to Mg imbalances. Additionally, the classified compounds were mainly primary metabolites. Most of the significant DEMs, particularly flavonoids, organic acids and lipids, were positively correlated with the measured physiological parameters of the mulberry plant (P_n_, chlorophyll, leaf Mg content and fresh weight). These compound classes were significantly implicated in lipid metabolism, amino acid metabolism, energy metabolism, the biosynthesis of other secondary metabolites, the biosynthesis of other amino acids and the metabolism of cofactors and vitamins in the KEGG pathways, indicating that mulberry responds to Mg imbalances by producing a divergent metabolism that regulates physiological responses. Altogether, different levels of Mg supply altered metabolic pathways differently, thus validating the hypothesis that different applications of Mg alter the morpho-physiological traits and metabolic pathways differently.

## Data Availability

The original data sets that support the findings of this study are openly available in the study is included in the article/Supplementary Materials. Further requests can be addressed to the corresponding author (Weiguo Zhao).

## Supplementary Materials

The supporting information can be downloaded at: https://www.mdpi.com/article/10.3390/ijms24119650/s1.
