# Supplementary material for: Magnesium Nutrient Application Induces Metabolomics and Physiological Responses in Mulberry (Morus alba) Plants
Source: Int J Mol Sci. 2023 Jun 2;24(11):9650. doi: 10.3390/ijms24119650 (PMC10253527; doi:10.3390/ijms24119650)
Supplement: Supplementary file 1 [file ijms-24-09650-s001.zip › Supplementary Figures.pdf]

Magnesium Nutrient Application Induces Metabolomics and Physiological Responses in Mulberry (*Morus alba*) Plants

Xin Jin[1], Michael Ackah[1,2*], Lei Wang[1], Frank Kwarteng Amoako[3], Yisu Shi[1], Lionnelle Gyllye Essoh[1], Jianbin Li[1], Qiaonan Zhang[1], Haonan Li[1], Weiguo Zhao[1*]

Supplementary figures

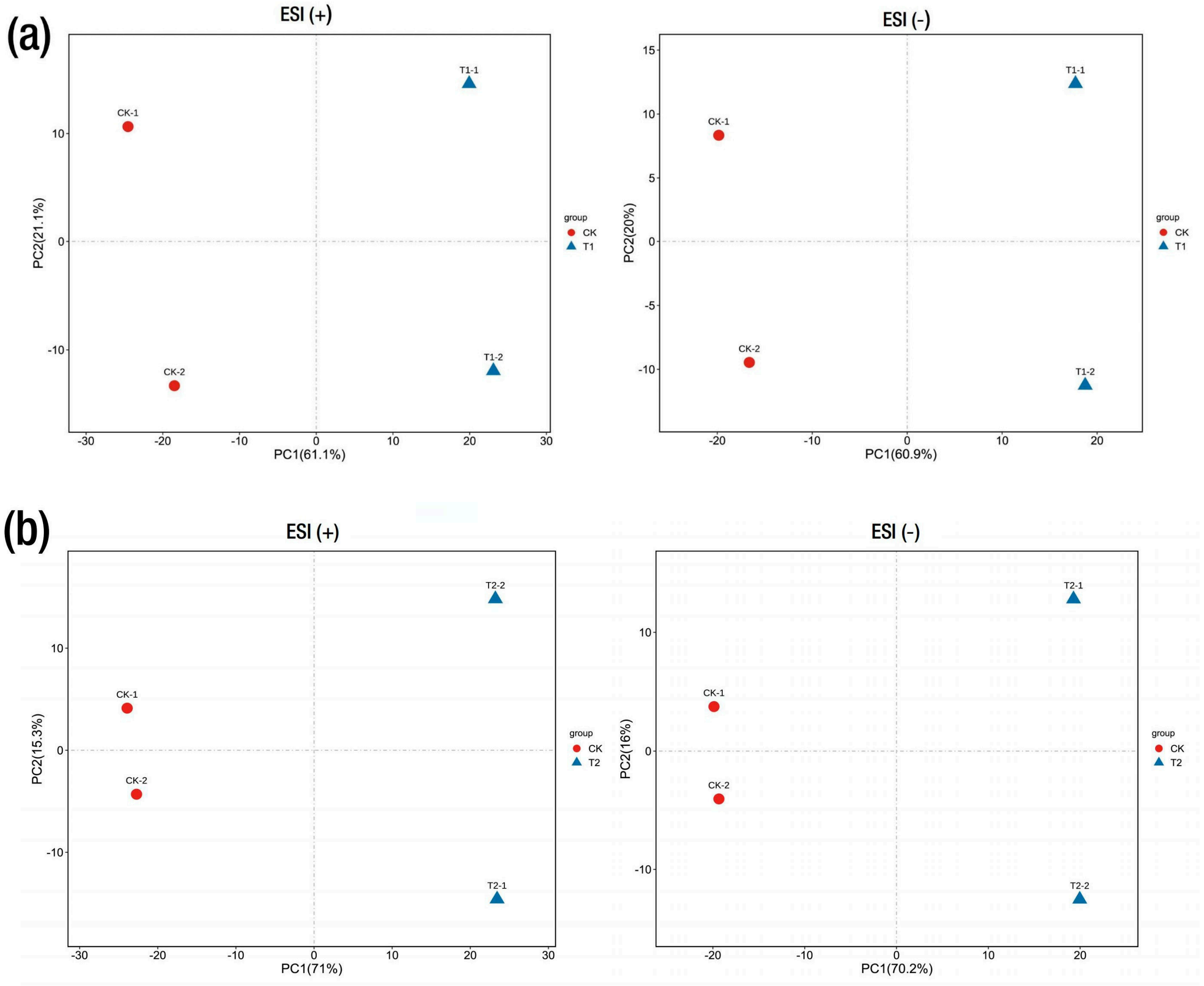

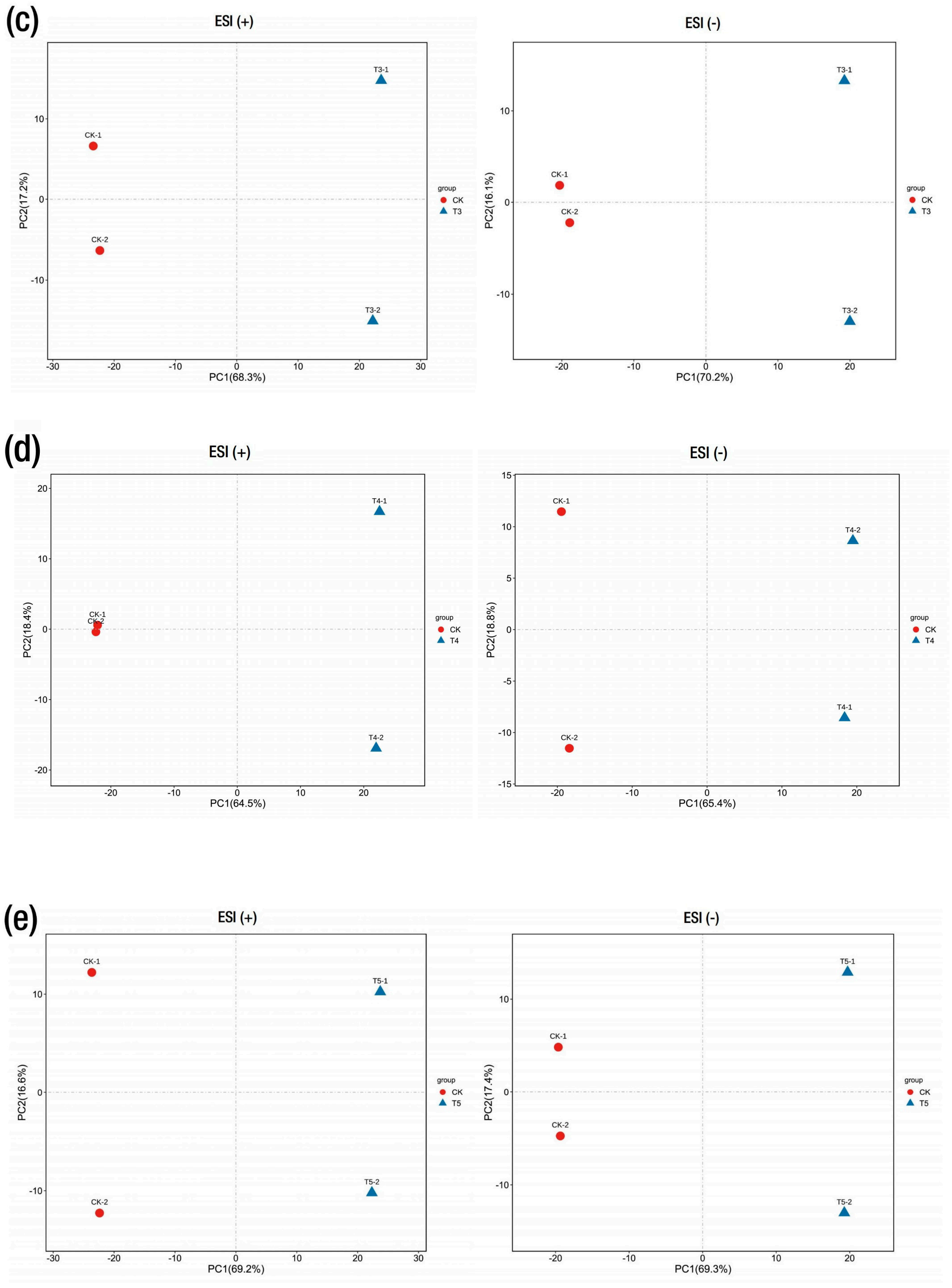
(c)
ESI (+)
PC1(68.3%)
PC2(17.2%)
CK-1
CK-2
T3-1
T3-2
group
CK
T3
ESI (-)
PC1(70.2%)
PC2(16.1%)
CK-1
CK-2
T3-1
T3-2
group
CK
T3
(d)
ESI (+)
PC1(64.5%)
PC2(18.4%)
CK-1
CK-2
T4-1
T4-2
group
CK
T4
ESI (-)
PC1(65.4%)
PC2(18.8%)
CK-1
CK-2
T4-1
T4-2
group
CK
T4
(e)
ESI (+)
PC1(69.2%)
PC2(16.6%)
CK-1
CK-2
T5-1
T5-2
group
CK
T5
ESI (-)
PC1(69.3%)
PC2(17.4%)
CK-1
CK-2
T5-1
T5-2
group
CK
T5

Figure S1. Sample PCA analysis chart plot. The PC1 coordinate represents the first principal component, and the percentage in parentheses indicates the contribution value of the first principal component to the sample difference; The PC2 coordinate represents the second principal component, and the percentage in parentheses indicates the contribution of the second principal component to the sample difference. The colored dots in the graph represent each sample, and the closer the same set of samples is, the better the sample repeatability (a) CK-T1. (b) CK-T2. (c) CK-T3. (d) CK-T4. (e) CK-T5. ESI (+); positive ion mode, ESI (-); negative ion mode. CK; sufficiency (3mmol/L), T1; 0mmol/L, T2; 1mmol/L, T3; 2mmol/L, T4; 6mmol/L, T5; 9mmol/L.

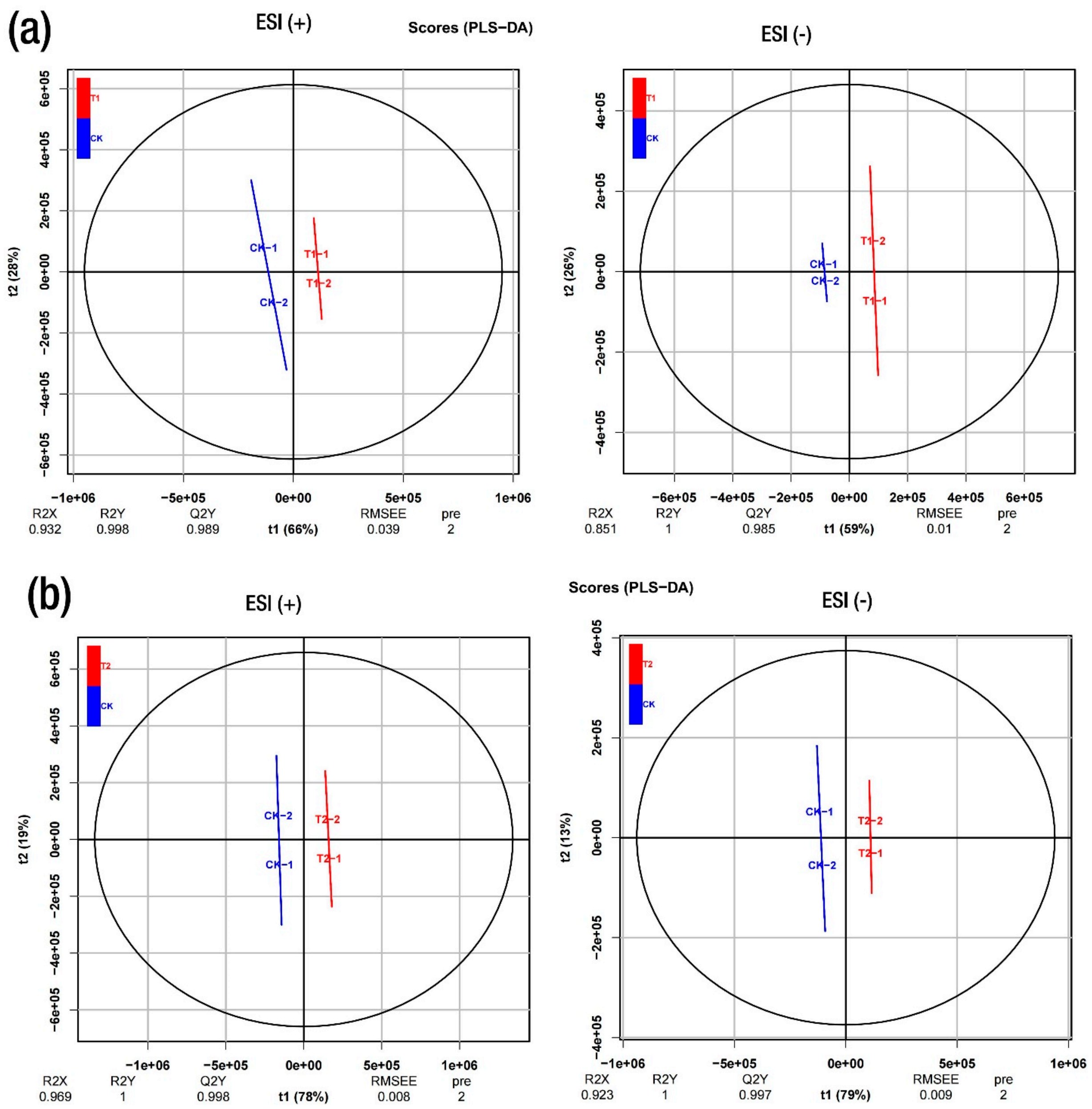

(c)

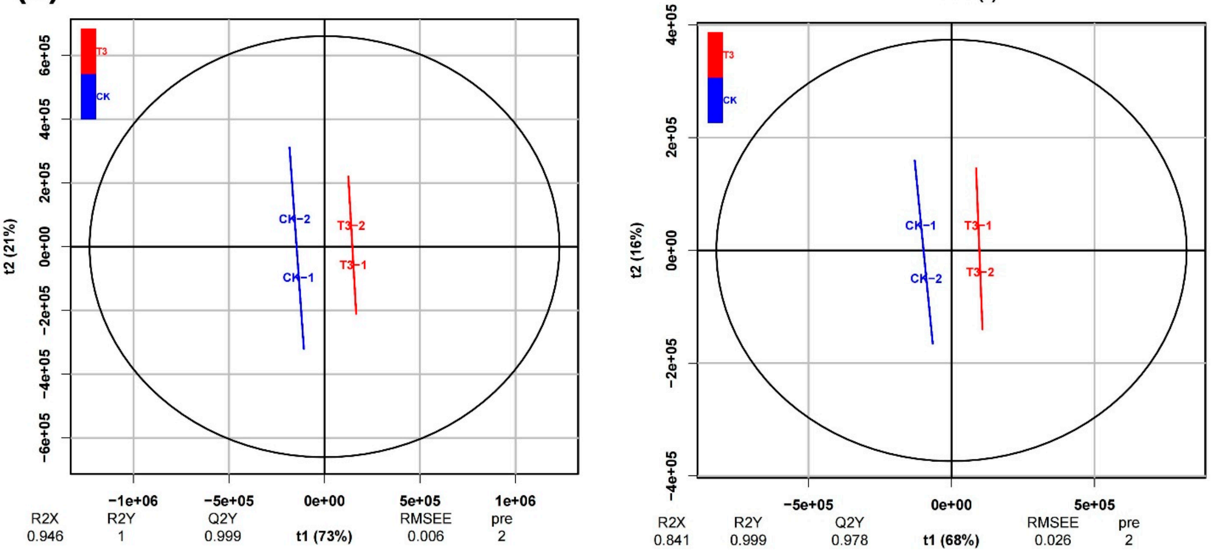

ESI (+)
T3
CK
CK−2
CK−1
T3−2
T3−1
t2 (21%)
t1 (73%)
R2X 0.946
R2Y 1
Q2Y 0.999
RMSEE 0.006
pre 2
ESI (-)
T3
CK
CK−1
CK−2
T3−1
T3−2
t2 (16%)
t1 (68%)
R2X 0.841
R2Y 0.999
Q2Y 0.978
RMSEE 0.026
pre 2

(d)

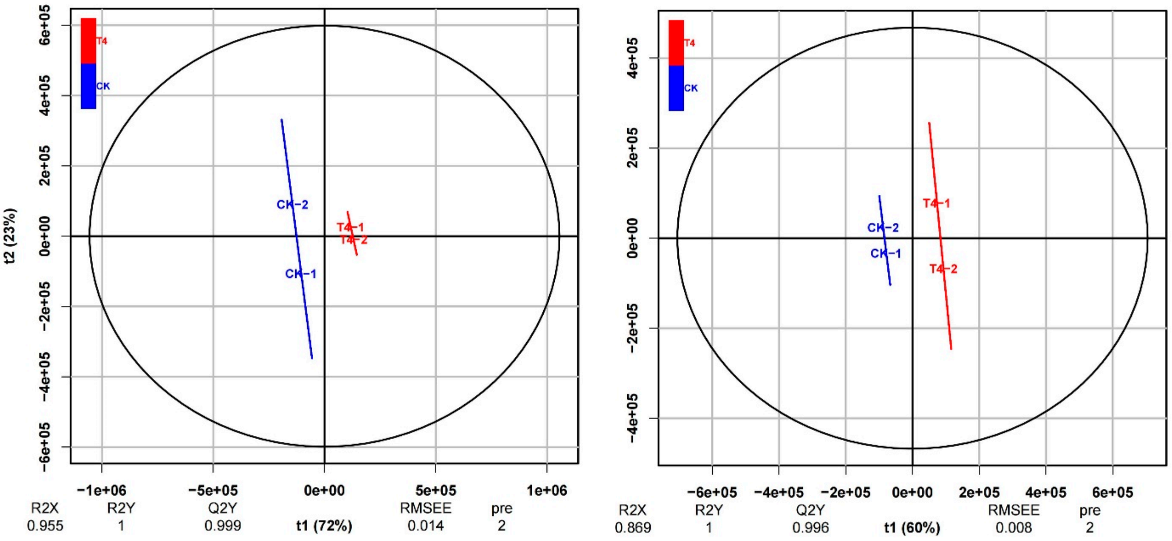

ESI (+)
T4
CK
CK−2
CK−1
T4−1
T4−2
t2 (23%)
t1 (72%)
R2X 0.955
R2Y 1
Q2Y 0.999
RMSEE 0.014
pre 2
ESI (-)
T4
CK
CK−2
CK−1
T4−1
T4−2
t1 (60%)
R2X 0.869
R2Y 1
Q2Y 0.996
RMSEE 0.008
pre 2

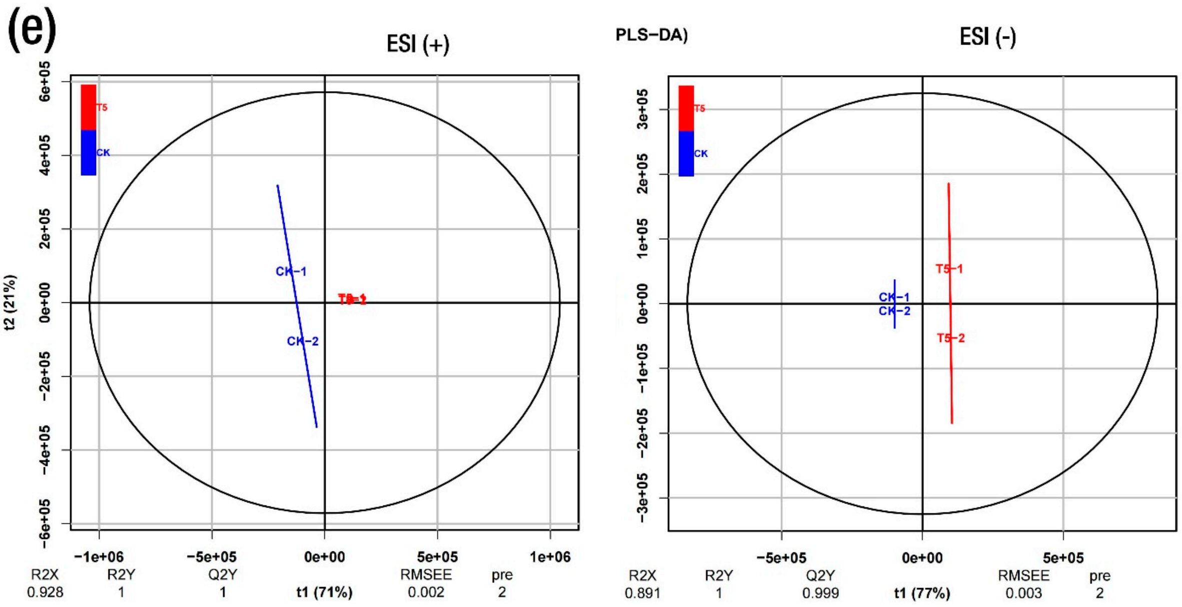

Figure S2. Partial least-squares-discriminant analysis (PLS-DA) scores of the metabolites from various Mg treatment in mulberry. (a) CK-T1 (b) CK-T2 (c) CK-T3 (d) CK-T4 (e) CK-T5. The PLS-DA models are R2X, R2Y and Q2. Where R2X and R2Y represent the interpretation rate of the built model to X and Y matrices, respectively, Q2 represents the prediction ability of the model, and the closer these three indicators are to 1, the more stable and reliable the model. Q2>0.5 indicates that the model has good prediction ability, and Q2 >0.9 indicates that the model is excellent. ESI (+); positive ion mode, ESI (-); negative ion mode.

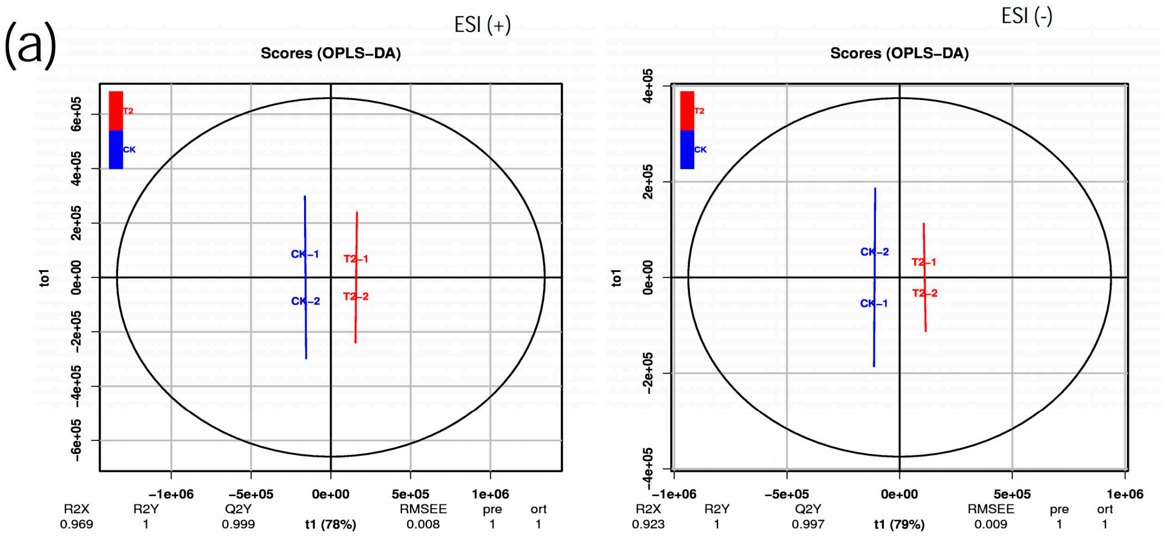

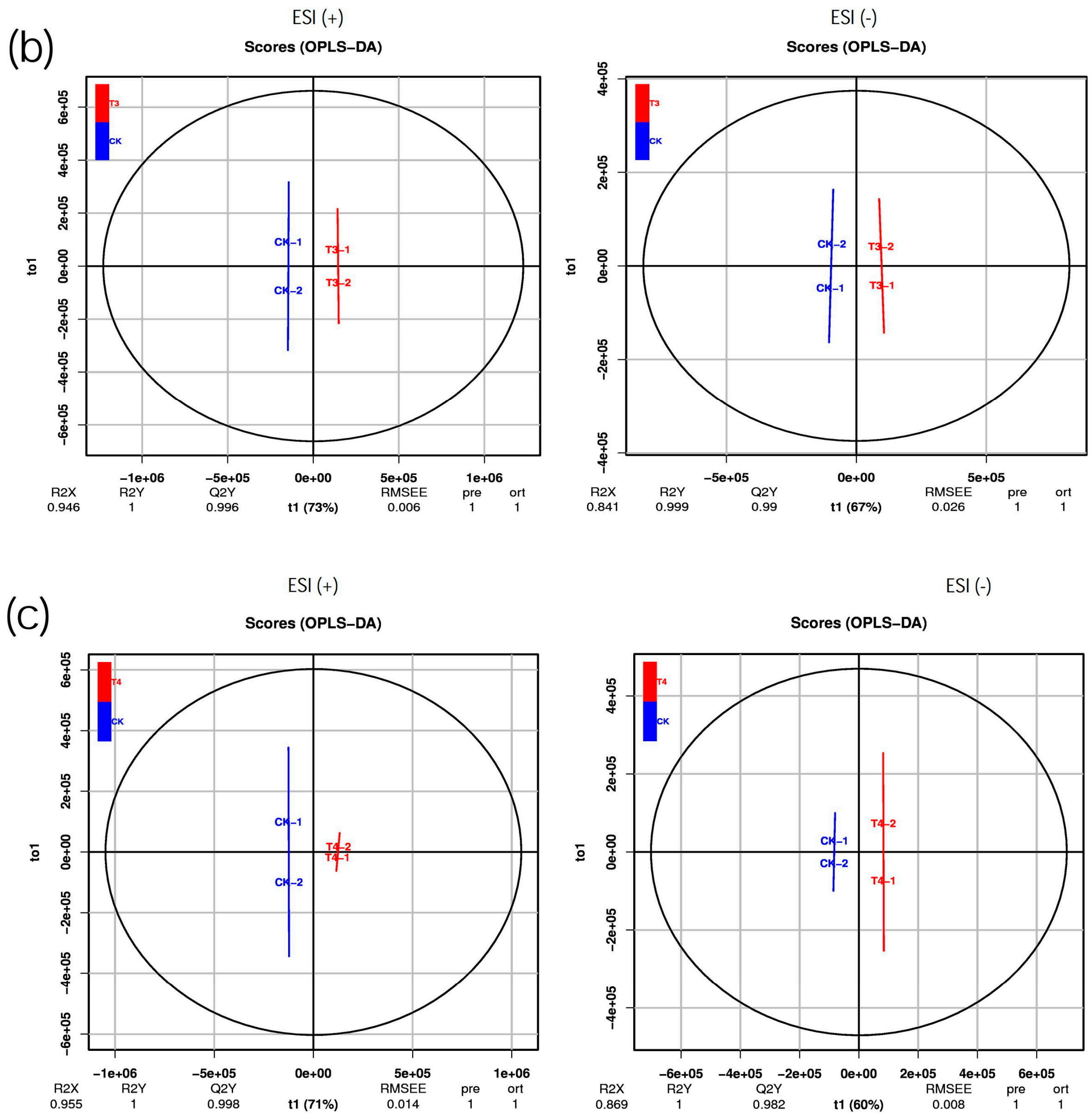

Figure S3. Orthogonal partial least-squares-discriminant analysis (OPLS-DA) scores of the metabolites (a) CK-T2 (b) CK-T3 (c) CK-T4. The OPLS-DA models are R2X, R2Y and Q2. Where R2X and R2Y represent the interpretation rate of the built model to X and Y matrices, respectively, Q2 represents the prediction ability of the model, and the closer these three indicators are to 1, the more stable and reliable the model. Q2>0.5 indicates that the model has good prediction ability, and Q2 >0.9 indicates that the model is excellent. ESI (+); positive ion mode, ESI (-); negative ion mode.

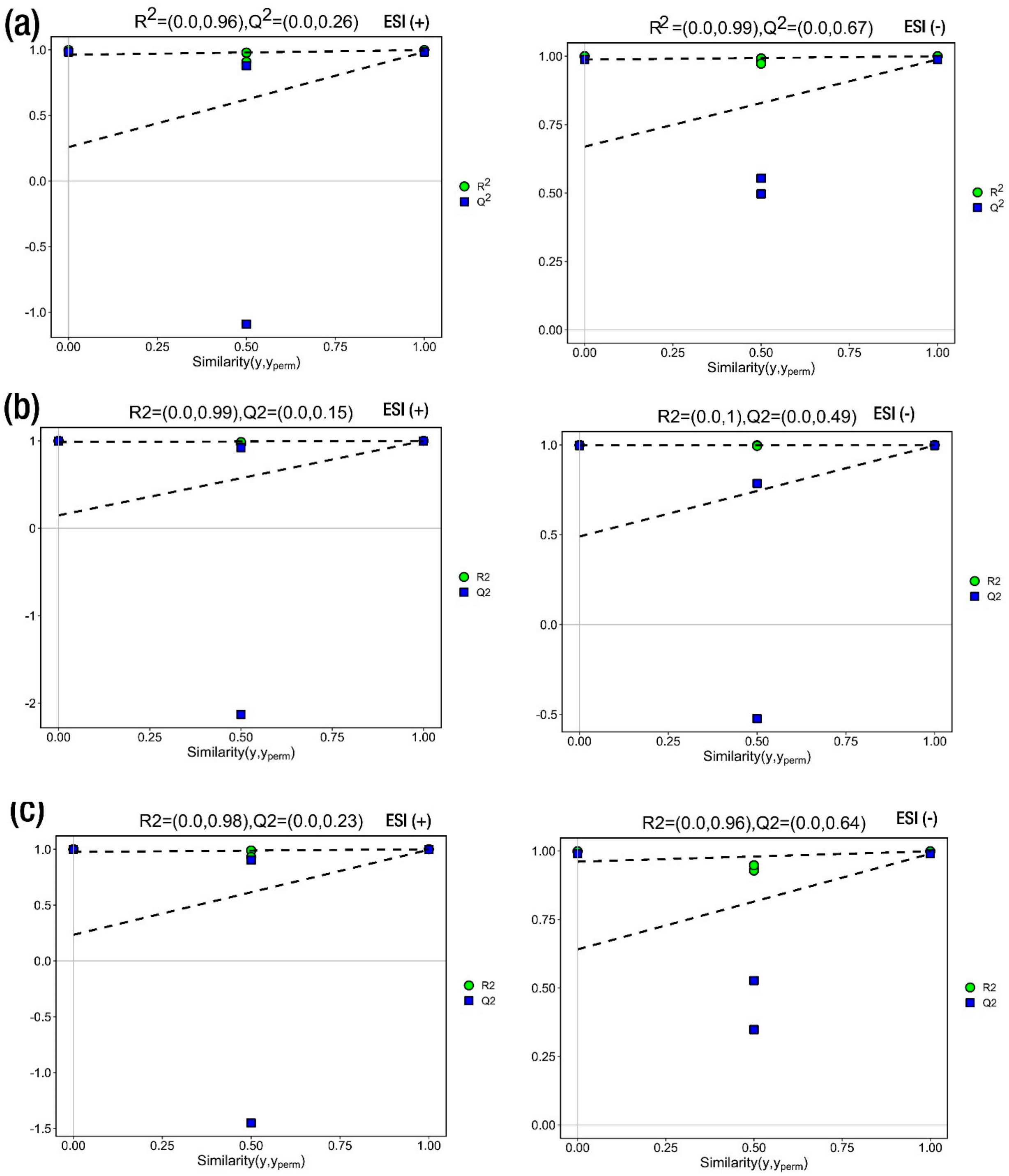

(a)
R2=(0.0,0.96),Q2=(0.0,0.26) ESI (+)
R2 =(0.0,0.99),Q2=(0.0,0.67) ESI (-)
(b)
R2=(0.0,0.99),Q2=(0.0,0.15) ESI (+)
R2=(0.0,1),Q2=(0.0,0.49) ESI (-)
(c)
R2=(0.0,0.98),Q2=(0.0,0.23) ESI (+)
R2=(0.0,0.96),Q2=(0.0,0.64) ESI (-)
Similarity(y,yperm)
R2
Q2

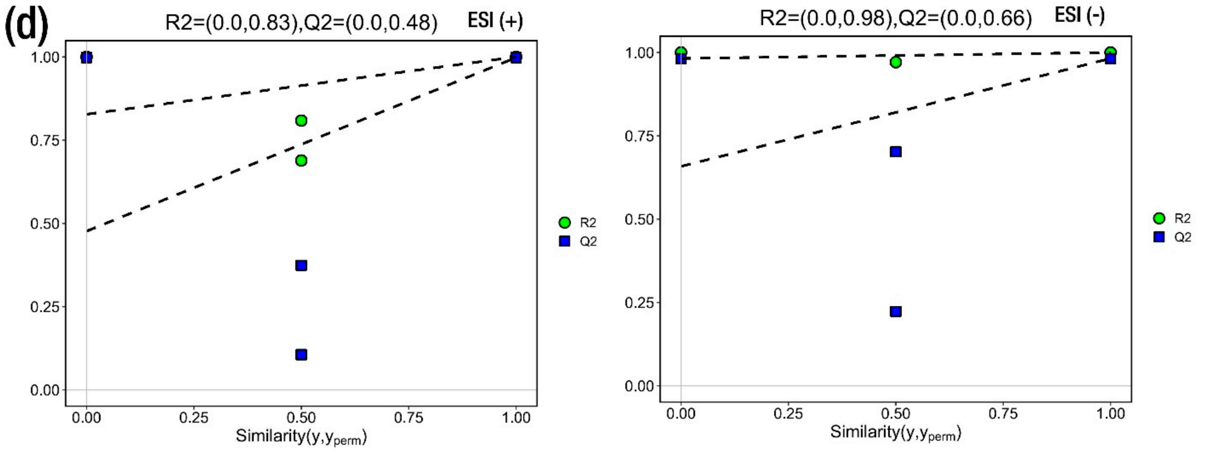

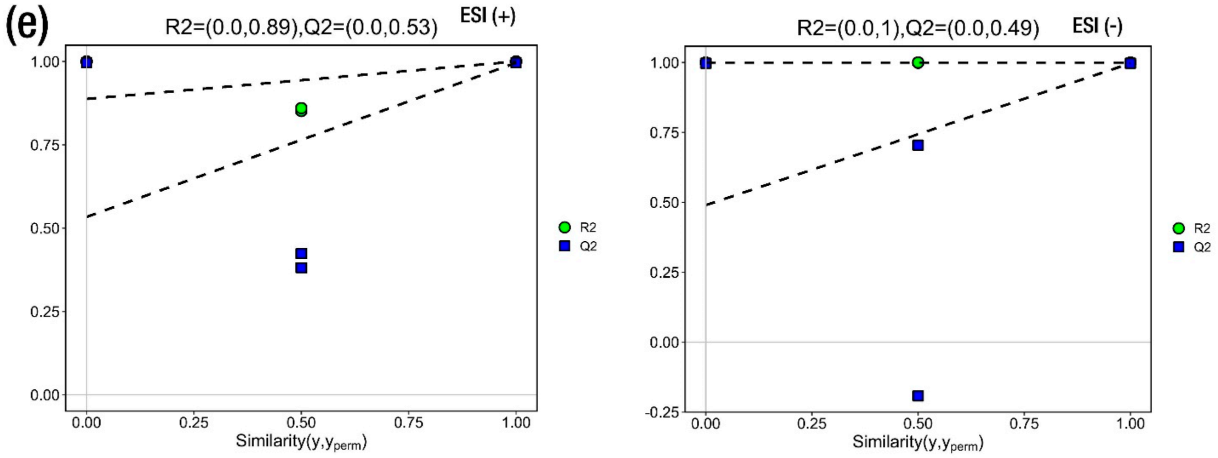

Figure S4. Permutation test A random ranking method used to evaluate the accuracy of (O)PLS models. (a) CK-T1 (b) CK-T2 (c) CK-T3 (d) CK-T4 (e) CK-T5. The two rightmost points (x=1.0) are R2 and Q2 of the original model, and all the points on the left are the R2' and Q2' of the model after Y displacement, if all the blue Q2 points from left to left are lower than the original blue Q2 points on the far right, or the regression line of Q2 points is less than or equal to 0 at the intersection of ordinates, it means that the model prediction results are reliable.

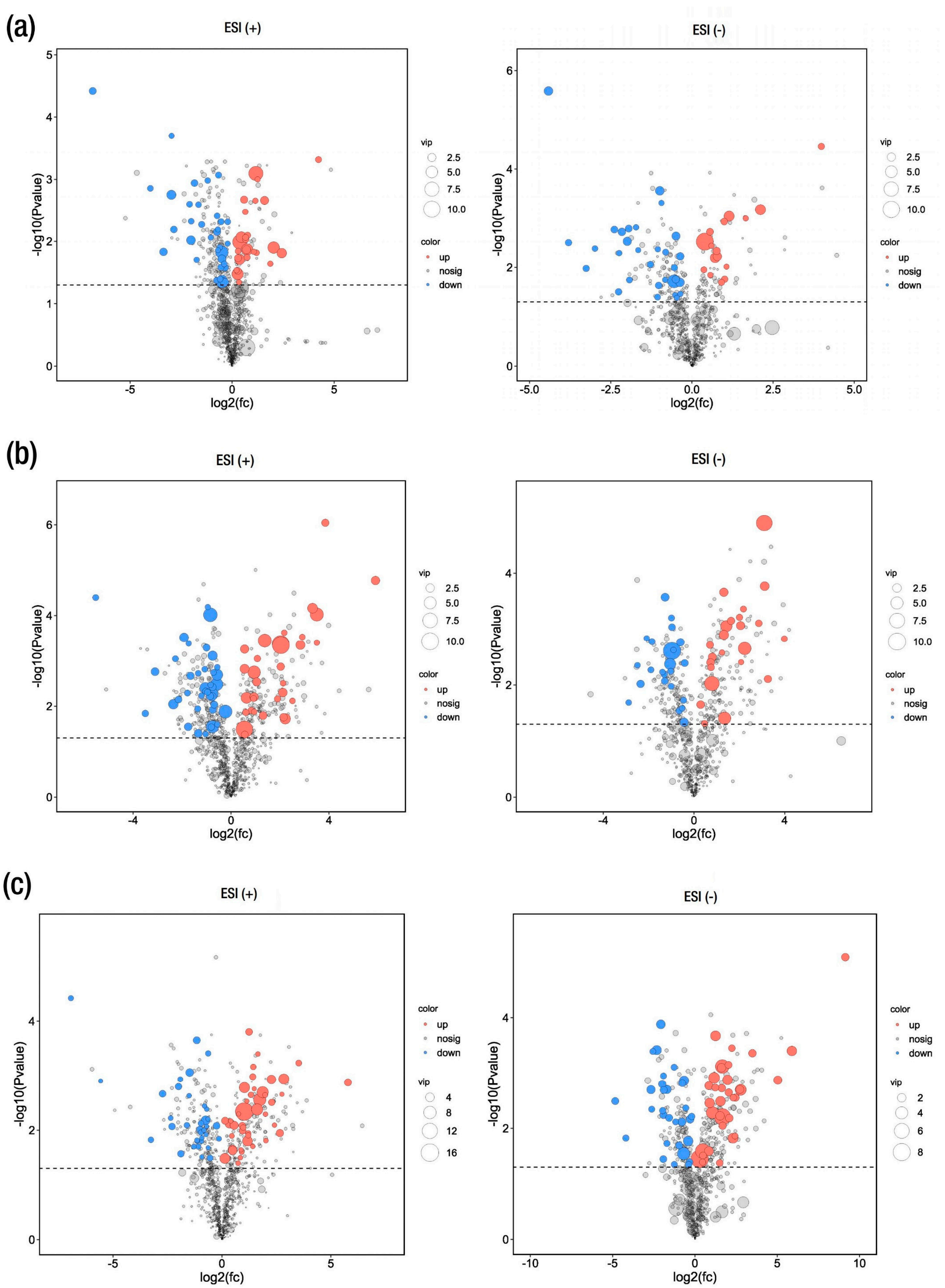

(a)
ESI (+)
ESI (-)
(b)
ESI (+)
ESI (-)
(c)
ESI (+)
ESI (-)
-log10(Pvalue)
log2(fc)
vip
2.5
5.0
7.5
10.0
color
up
nosig
down
vip
4
8
12
16
vip
2
4
6
8

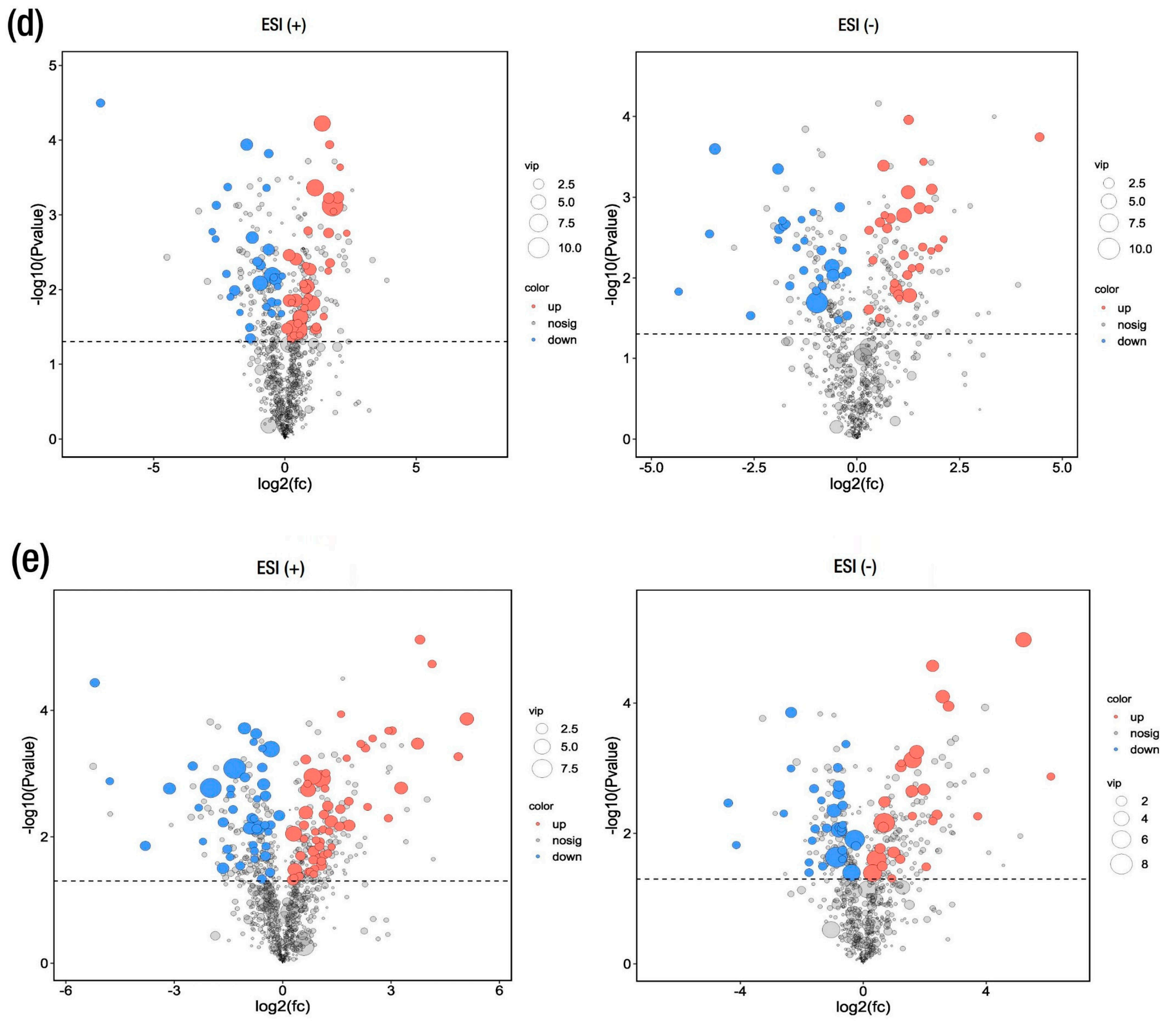

Figure S5. Volcano plot of the differential metabolites from various Mg treatment in mulberry. (a) CK-T1 (b) CK-T2 (c) CK-T3 (d) CK-T4 (e) CK-T5. ESI (+); positive ion mode, ESI (-); negative ion mode. The abscissa is the value after $\log_2$ for the different multiple of the metabolite abundance in each comparison group, the ordinate is the p-value after the t-test, and the dashed line perpendicular to the y-axis is the p-value threshold for differential metabolite screening. The red dots represent VIP≥1 and p<0.05, differential metabolites that are up-regulated (FC>1); The blue dots represent VIP≥1 and p<0.05, differentiating metabolites that are downregulated (FC<-1). The larger the point, the greater the metabolite VIP value.

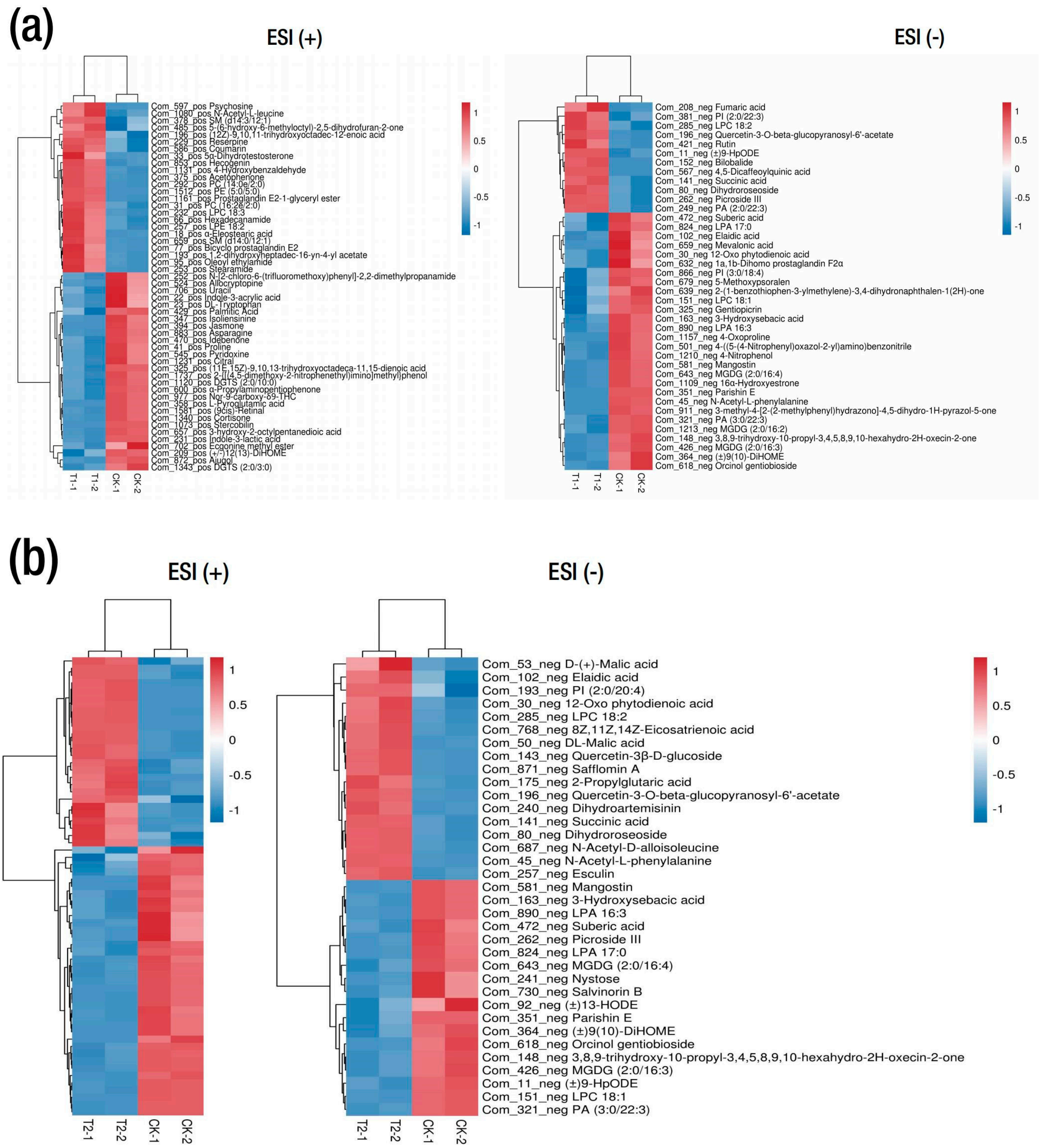

(a)
ESI (+)
Com_597_pos Psychosine
Com_1080_pos N-Acetyl-L-leucine
Com_378_pos SM (d14:3/12:1)
Com_485_pos 5-(6-hydroxy-6-methyloctyl)-2,5-dihydrofuran-2-one
Com_196_pos (12Z)-9,10,11-trihydroxyoctadec-12-enoic acid
Com_229_pos Reserpine
Com_586_pos Coumarin
Com_33_pos 5α-Dihydrotestosterone
Com_853_pos Hecogenin
Com_1131_pos 4-Hydroxybenzaldehyde
Com_375_pos Acetophenone
Com_292_pos PC (14:0e/2:0)
Com_1512_pos PE (5:0/5:0)
Com_1161_pos Prostaglandin E2-1-glyceryl ester
Com_31_pos PC (16:2e/2:0)
Com_232_pos LPC 18:3
Com_66_pos Hexadecanamide
Com_257_pos LPE 18:2
Com_18_pos α-Eleostearic acid
Com_659_pos SM (d14:0/12:1)
Com_77_pos Bicyclo prostaglandin E2
Com_193_pos 1,2-dihydroxyheptadec-16-yn-4-yl acetate
Com_95_pos Oleoyl ethylamide
Com_253_pos Stearamide
Com_252_pos N-[2-chloro-6-(trifluoromethoxy)phenyl]-2,2-dimethylpropanamide
Com_524_pos Allocryptopine
Com_706_pos Uracil
Com_22_pos Indole-3-acrylic acid
Com_23_pos DL-Tryptophan
Com_429_pos Palmitic Acid
Com_347_pos Isoliensinine
Com_394_pos Jasmone
Com_883_pos Asparagine
Com_470_pos Idebenone
Com_41_pos Proline
Com_545_pos Pyridoxine
Com_1231_pos Citral
Com_325_pos (11E,15Z)-9,10,13-trihydroxyoctadeca-11,15-dienoic acid
Com_1737_pos 2-{[(4,5-dimethoxy-2-nitrophenethyl)imino]methyl}phenol
Com_1120_pos DGTS (2:0/10:0)
Com_600_pos α-Propylaminopentiophenone
Com_977_pos Nor-9-carboxy-δ9-THC
Com_358_pos L-Pyroglutamic acid
Com_1581_pos (9cis)-Retinal
Com_1340_pos Cortisone
Com_1073_pos Stercobilin
Com_657_pos 3-hydroxy-2-octylpentanedioic acid
Com_231_pos Indole-3-lactic acid
Com_702_pos Ecgonine methyl ester
Com_209_pos (+/-)12(13)-DiHOME
Com_872_pos Ajugol
Com_1343_pos DGTS (2:0/3:0)
T1-1
T1-2
CK-1
CK-2
1
0.5
0
-0.5
-1
ESI (-)
Com_208_neg Fumaric acid
Com_381_neg PI (2:0/22:3)
Com_285_neg LPC 18:2
Com_196_neg Quercetin-3-O-beta-glucopyranosyl-6'-acetate
Com_421_neg Rutin
Com_11_neg (±)9-HpODE
Com_152_neg Bilobalide
Com_567_neg 4,5-Dicaffeoylquinic acid
Com_141_neg Succinic acid
Com_80_neg Dihydroroseoside
Com_262_neg Picroside III
Com_249_neg PA (2:0/22:3)
Com_472_neg Suberic acid
Com_824_neg LPA 17:0
Com_102_neg Elaidic acid
Com_659_neg Mevalonic acid
Com_30_neg 12-Oxo phytodienoic acid
Com_632_neg 1a,1b-Dihomo prostaglandin F2α
Com_866_neg PI (3:0/18:4)
Com_679_neg 5-Methoxypsoralen
Com_639_neg 2-(1-benzothiophen-3-ylmethylene)-3,4-dihydronaphthalen-1(2H)-one
Com_151_neg LPC 18:1
Com_325_neg Gentiopicrin
Com_163_neg 3-Hydroxysebacic acid
Com_890_neg LPA 16:3
Com_1157_neg 4-Oxoproline
Com_501_neg 4-((5-(4-Nitrophenyl)oxazol-2-yl)amino)benzonitrile
Com_1210_neg 4-Nitrophenol
Com_581_neg Mangostin
Com_643_neg MGDG (2:0/16:4)
Com_1109_neg 16α-Hydroxyestrone
Com_351_neg Parishin E
Com_45_neg N-Acetyl-L-phenylalanine
Com_911_neg 3-methyl-4-[2-(2-methylphenyl)hydrazono]-4,5-dihydro-1H-pyrazol-5-one
Com_321_neg PA (3:0/22:3)
Com_1213_neg MGDG (2:0/16:2)
Com_148_neg 3,8,9-trihydroxy-10-propyl-3,4,5,8,9,10-hexahydro-2H-oxecin-2-one
Com_426_neg MGDG (2:0/16:3)
Com_364_neg (±)9(10)-DiHOME
Com_618_neg Orcinol gentiobioside
T1-1
T1-2
CK-1
CK-2
1
0.5
0
-0.5
-1
(b)
ESI (+)
1
0.5
0
-0.5
-1
T2-1
T2-2
CK-1
CK-2
ESI (-)
Com_53_neg D-(+)-Malic acid
Com_102_neg Elaidic acid
Com_193_neg PI (2:0/20:4)
Com_30_neg 12-Oxo phytodienoic acid
Com_285_neg LPC 18:2
Com_768_neg 8Z,11Z,14Z-Eicosatrienoic acid
Com_50_neg DL-Malic acid
Com_143_neg Quercetin-3β-D-glucoside
Com_871_neg Safflomin A
Com_175_neg 2-Propylglutaric acid
Com_196_neg Quercetin-3-O-beta-glucopyranosyl-6'-acetate
Com_240_neg Dihydroartemisinin
Com_141_neg Succinic acid
Com_80_neg Dihydroroseoside
Com_687_neg N-Acetyl-D-alloisoleucine
Com_45_neg N-Acetyl-L-phenylalanine
Com_257_neg Esculin
Com_581_neg Mangostin
Com_163_neg 3-Hydroxysebacic acid
Com_890_neg LPA 16:3
Com_472_neg Suberic acid
Com_262_neg Picroside III
Com_824_neg LPA 17:0
Com_643_neg MGDG (2:0/16:4)
Com_241_neg Nystose
Com_730_neg Salvinorin B
Com_92_neg (±)13-HODE
Com_351_neg Parishin E
Com_364_neg (±)9(10)-DiHOME
Com_618_neg Orcinol gentiobioside
Com_148_neg 3,8,9-trihydroxy-10-propyl-3,4,5,8,9,10-hexahydro-2H-oxecin-2-one
Com_426_neg MGDG (2:0/16:3)
Com_11_neg (±)9-HpODE
Com_151_neg LPC 18:1
Com_321_neg PA (3:0/22:3)
1
0.5
0
-0.5
-1
T2-1
T2-2
CK-1
CK-2

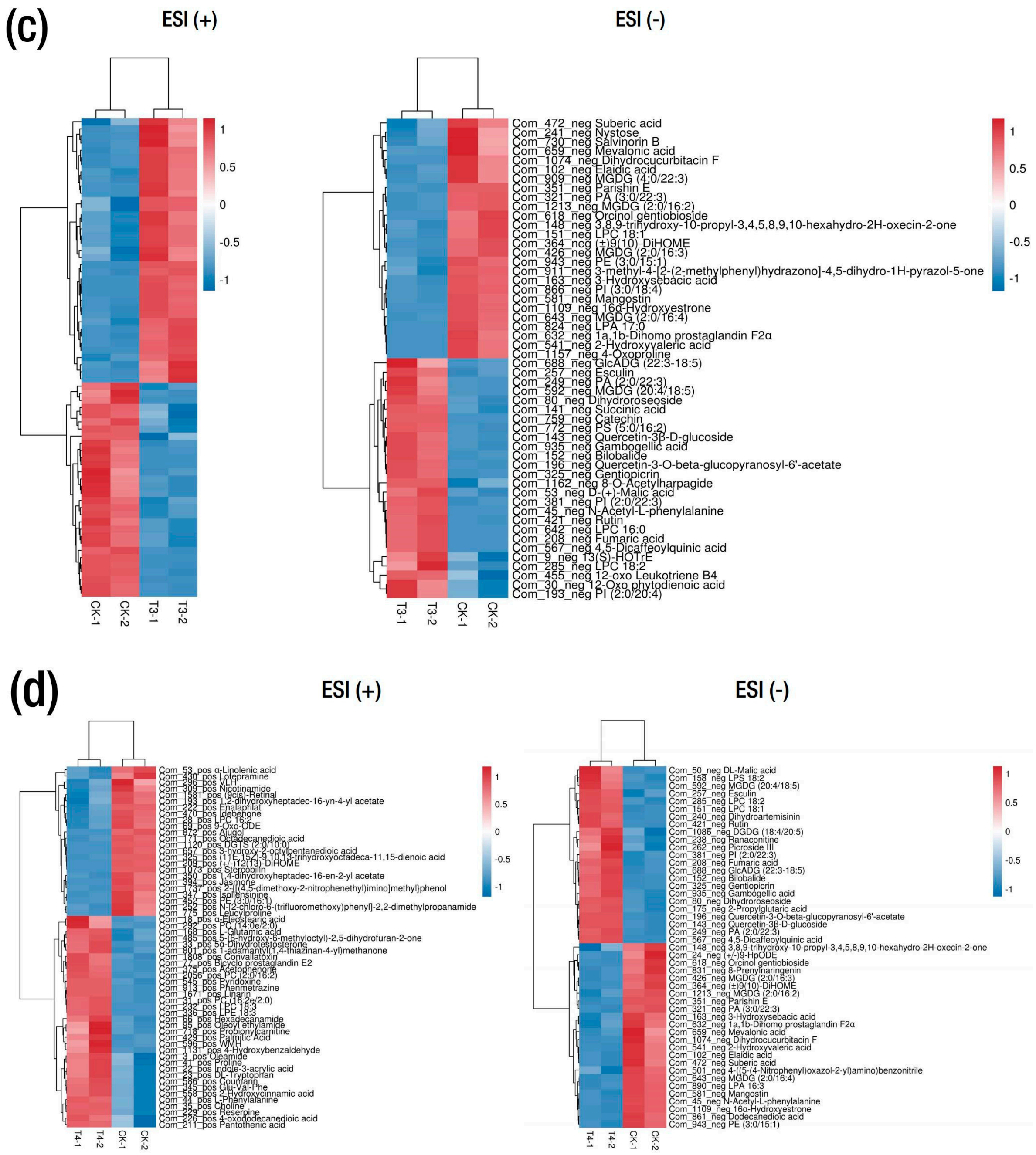
(c)
ESI (+)
1
0.5
0
-0.5
-1
CK-1
CK-2
T3-1
T3-2
ESI (-)
Com_472_neg Suberic acid
Com_241_neg Nystose
Com_730_neg Salvinorin B
Com_659_neg Mevalonic acid
Com_1074_neg Dihydrocucurbitacin F
Com_102_neg Elaidic acid
Com_909_neg MGDG (4:0/22:3)
Com_351_neg Parishin E
Com_321_neg PA (3:0/22:3)
Com_1213_neg MGDG (2:0/16:2)
Com_618_neg Orcinol gentiobioside
Com_148_neg 3,8,9-trihydroxy-10-propyl-3,4,5,8,9,10-hexahydro-2H-oxecin-2-one
Com_151_neg LPC 18:1
Com_364_neg (±)9(10)-DiHOME
Com_426_neg MGDG (2:0/16:3)
Com_943_neg PE (3:0/15:1)
Com_911_neg 3-methyl-4-[2-(2-methylphenyl)hydrazono]-4,5-dihydro-1H-pyrazol-5-one
Com_163_neg 3-Hydroxysebacic acid
Com_866_neg PI (3:0/18:4)
Com_581_neg Mangostin
Com_1109_neg 16α-Hydroxyestrone
Com_643_neg MGDG (2:0/16:4)
Com_824_neg LPA 17:0
Com_632_neg 1a,1b-Dihomo prostaglandin F2α
Com_541_neg 2-Hydroxyvaleric acid
Com_1157_neg 4-Oxoproline
Com_688_neg GlcADG (22:3-18:5)
Com_257_neg Esculin
Com_249_neg PA (2:0/22:3)
Com_592_neg MGDG (20:4/18:5)
Com_80_neg Dihydroroseoside
Com_141_neg Succinic acid
Com_759_neg Catechin
Com_772_neg PS (5:0/16:2)
Com_143_neg Quercetin-3β-D-glucoside
Com_935_neg Gambogellic acid
Com_152_neg Bilobalide
Com_196_neg Quercetin-3-O-beta-glucopyranosyl-6'-acetate
Com_325_neg Gentiopicrin
Com_1162_neg 8-O-Acetylharpagide
Com_53_neg D-(+)-Malic acid
Com_381_neg PI (2:0/22:3)
Com_45_neg N-Acetyl-L-phenylalanine
Com_421_neg Rutin
Com_642_neg LPC 16:0
Com_208_neg Fumaric acid
Com_567_neg 4,5-Dicaffeoylquinic acid
Com_9_neg 13(S)-HOTrE
Com_285_neg LPC 18:2
Com_455_neg 12-oxo Leukotriene B4
Com_30_neg 12-Oxo phytodienoic acid
Com_193_neg PI (2:0/20:4)
1
0.5
0
-0.5
-1
T3-1
T3-2
CK-1
CK-2
(d)
ESI (+)
Com_53_pos α-Linolenic acid
Com_430_pos Lofepramine
Com_296_pos VLH
Com_309_pos Nicotinamide
Com_1581_pos (9cis)-Retinal
Com_193_pos 1,2-dihydroxyheptadec-16-yn-4-yl acetate
Com_222_pos Enalaprilat
Com_470_pos Idebenone
Com_28_pos LPC 16:2
Com_69_pos 9-Oxo-ODE
Com_872_pos Ajugol
Com_171_pos Octadecanedioic acid
Com_1120_pos DGTS (2:0/10:0)
Com_657_pos 3-hydroxy-2-octylpentanedioic acid
Com_325_pos (11E,15Z)-9,10,13-trihydroxyoctadeca-11,15-dienoic acid
Com_209_pos (+/-)12(13)-DiHOME
Com_1073_pos Stercobilin
Com_350_pos 1,4-dihydroxyheptadec-16-en-2-yl acetate
Com_394_pos Jasmone
Com_1737_pos 2-{[(4,5-dimethoxy-2-nitrophenethyl)imino]methyl}phenol
Com_347_pos Isoliensinine
Com_452_pos PE (3:0/16:1)
Com_252_pos N-[2-chloro-6-(trifluoromethoxy)phenyl]-2,2-dimethylpropanamide
Com_775_pos Leucylproline
Com_18_pos α-Eleostearic acid
Com_292_pos PC (14:0e/2:0)
Com_168_pos L-Glutamic acid
Com_485_pos 5-(6-hydroxy-6-methyloctyl)-2,5-dihydrofuran-2-one
Com_33_pos 5α-Dihydrotestosterone
Com_801_pos 1-adamantyl(1,4-thiazinan-4-yl)methanone
Com_1808_pos Convallatoxin
Com_77_pos Bicyclo prostaglandin E2
Com_375_pos Acetophenone
Com_2056_pos PC (2:0/16:2)
Com_545_pos Pyridoxine
Com_913_pos Phenmetrazine
Com_1671_pos Linarin
Com_31_pos PC (16:2e/2:0)
Com_232_pos LPC 18:3
Com_336_pos LPE 18:3
Com_66_pos Hexadecanamide
Com_95_pos Oleoyl ethylamide
Com_718_pos Propionylcarnitine
Com_429_pos Palmitic Acid
Com_596_pos WMH
Com_1131_pos 4-Hydroxybenzaldehyde
Com_3_pos Oleamide
Com_41_pos Proline
Com_22_pos Indole-3-acrylic acid
Com_23_pos DL-Tryptophan
Com_586_pos Coumarin
Com_345_pos Glu-Val-Phe
Com_558_pos 2-Hydroxycinnamic acid
Com_44_pos L-Phenylalanine
Com_35_pos Choline
Com_229_pos Reserpine
Com_226_pos 4-oxododecanedioic acid
Com_211_pos Pantothenic acid
1
0.5
0
-0.5
-1
T4-1
T4-2
CK-1
CK-2
ESI (-)
Com_50_neg DL-Malic acid
Com_158_neg LPS 18:2
Com_592_neg MGDG (20:4/18:5)
Com_257_neg Esculin
Com_285_neg LPC 18:2
Com_151_neg LPC 18:1
Com_240_neg Dihydroartemisinin
Com_421_neg Rutin
Com_1086_neg DGDG (18:4/20:5)
Com_238_neg Ranaconitine
Com_262_neg Picroside III
Com_381_neg PI (2:0/22:3)
Com_208_neg Fumaric acid
Com_688_neg GlcADG (22:3-18:5)
Com_152_neg Bilobalide
Com_325_neg Gentiopicrin
Com_935_neg Gambogellic acid
Com_80_neg Dihydroroseoside
Com_175_neg 2-Propylglutaric acid
Com_196_neg Quercetin-3-O-beta-glucopyranosyl-6'-acetate
Com_143_neg Quercetin-3β-D-glucoside
Com_249_neg PA (2:0/22:3)
Com_567_neg 4,5-Dicaffeoylquinic acid
Com_148_neg 3,8,9-trihydroxy-10-propyl-3,4,5,8,9,10-hexahydro-2H-oxecin-2-one
Com_24_neg (+/-)9-HpODE
Com_618_neg Orcinol gentiobioside
Com_831_neg 8-Prenylnaringenin
Com_426_neg MGDG (2:0/16:3)
Com_364_neg (±)9(10)-DiHOME
Com_1213_neg MGDG (2:0/16:2)
Com_351_neg Parishin E
Com_321_neg PA (3:0/22:3)
Com_163_neg 3-Hydroxysebacic acid
Com_632_neg 1a,1b-Dihomo prostaglandin F2α
Com_659_neg Mevalonic acid
Com_1074_neg Dihydrocucurbitacin F
Com_541_neg 2-Hydroxyvaleric acid
Com_102_neg Elaidic acid
Com_472_neg Suberic acid
Com_501_neg 4-((5-(4-Nitrophenyl)oxazol-2-yl)amino)benzonitrile
Com_643_neg MGDG (2:0/16:4)
Com_890_neg LPA 16:3
Com_581_neg Mangostin
Com_45_neg N-Acetyl-L-phenylalanine
Com_1109_neg 16α-Hydroxyestrone
Com_861_neg Dodecanedioic acid
Com_943_neg PE (3:0/15:1)
1
0.5
0
-0.5
-1
T4-1
T4-2
CK-1
CK-2

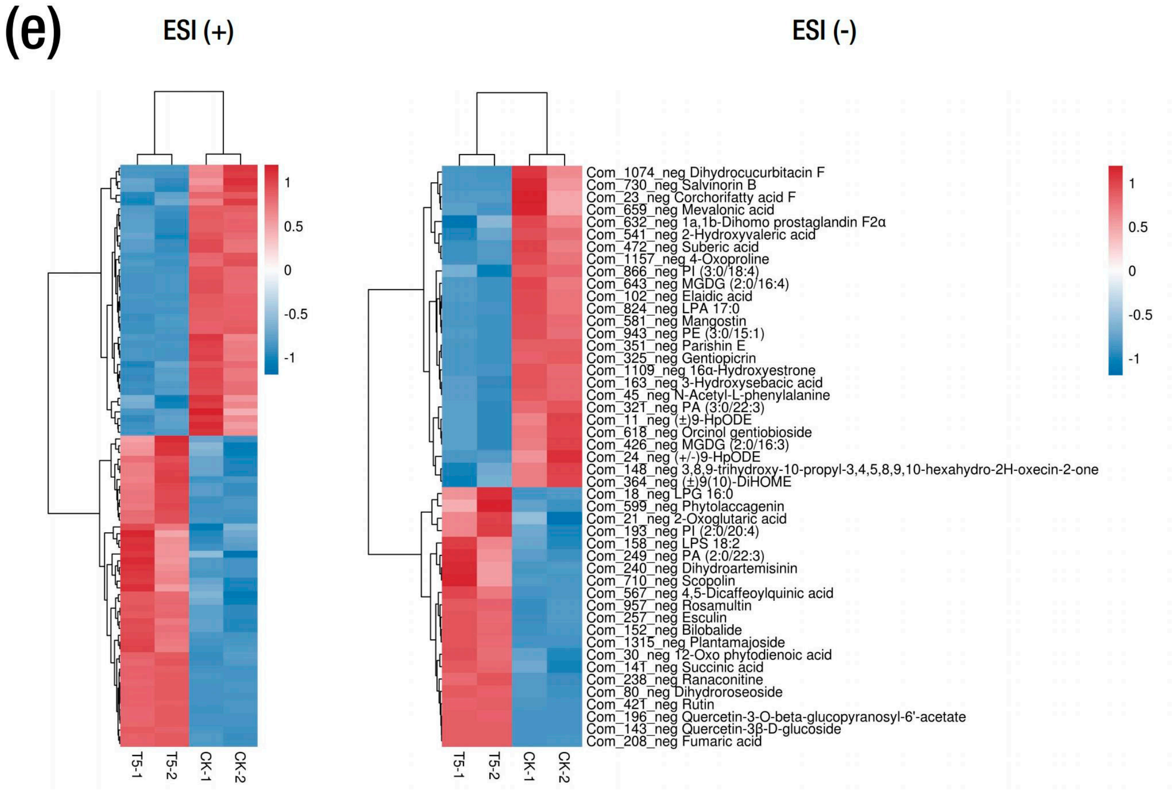

Figure S6. Differential metabolite clustering heatmap from various Mg treatment in mulberry. (a) CK-T1 (b) CK-T2 (c) CK-T3 (d) CK-T4 (e) CK-T5. ESI (+); positive ion mode, ESI (-); negative ion mode. Each row in the figure represents a metabolite, each column represents a sample, the different metabolites in each comparison group are normalized by rows, the red color, represent higher metabolite abundance, and the blue represents lower metabolite abundance. Metabolites and samples clustered separately according to their metabolite abundance, and the expression patterns of samples or metabolites under the same branch represent more similarity.

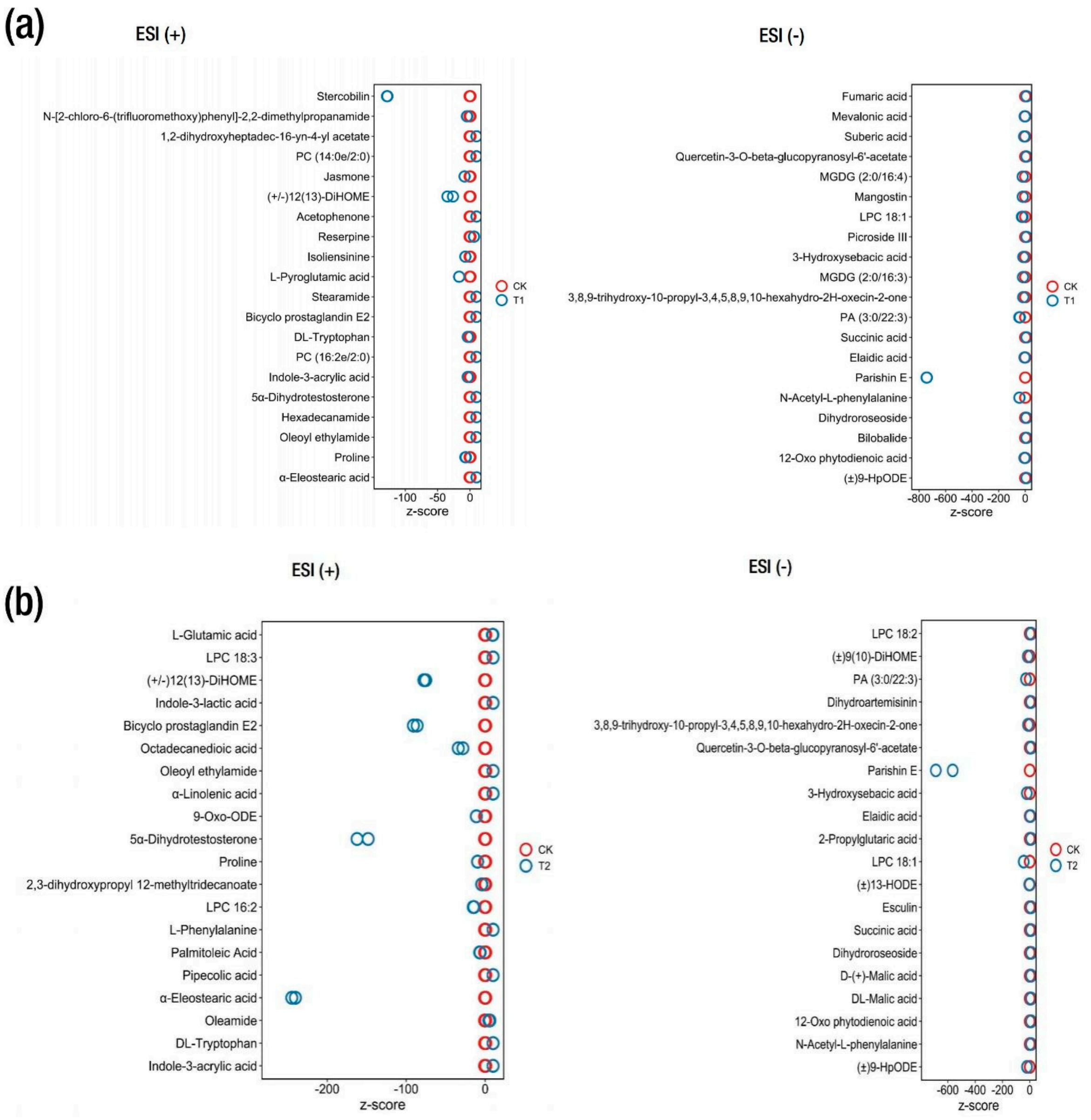
(a)
ESI (+)
Stercobilin
N-[2-chloro-6-(trifluoromethoxy)phenyl]-2,2-dimethylpropanamide
1,2-dihydroxyheptadec-16-yn-4-yl acetate
PC (14:0e/2:0)
Jasmone
(+/-)12(13)-DiHOME
Acetophenone
Reserpine
Isoliensinine
L-Pyroglutamic acid
Stearamide
Bicyclo prostaglandin E2
DL-Tryptophan
PC (16:2e/2:0)
Indole-3-acrylic acid
5α-Dihydrotestosterone
Hexadecanamide
Oleoyl ethylamide
Proline
α-Eleostearic acid
-100 -50 0
z-score
CK
T1
ESI (-)
Fumaric acid
Mevalonic acid
Suberic acid
Quercetin-3-O-beta-glucopyranosyl-6'-acetate
MGDG (2:0/16:4)
Mangostin
LPC 18:1
Picroside III
3-Hydroxysebacic acid
MGDG (2:0/16:3)
3,8,9-trihydroxy-10-propyl-3,4,5,8,9,10-hexahydro-2H-oxecin-2-one
PA (3:0/22:3)
Succinic acid
Elaidic acid
Parishin E
N-Acetyl-L-phenylalanine
Dihydroroseoside
Bilobalide
12-Oxo phytodienoic acid
(±)9-HpODE
-800 -600 -400 -200 0
z-score
CK
T1
(b)
ESI (+)
L-Glutamic acid
LPC 18:3
(+/-)12(13)-DiHOME
Indole-3-lactic acid
Bicyclo prostaglandin E2
Octadecanedioic acid
Oleoyl ethylamide
α-Linolenic acid
9-Oxo-ODE
5α-Dihydrotestosterone
Proline
2,3-dihydroxypropyl 12-methyltridecanoate
LPC 16:2
L-Phenylalanine
Palmitoleic Acid
Pipecolic acid
α-Eleostearic acid
Oleamide
DL-Tryptophan
Indole-3-acrylic acid
-200 -100 0
z-score
CK
T2
ESI (-)
LPC 18:2
(±)9(10)-DiHOME
PA (3:0/22:3)
Dihydroartemisinin
3,8,9-trihydroxy-10-propyl-3,4,5,8,9,10-hexahydro-2H-oxecin-2-one
Quercetin-3-O-beta-glucopyranosyl-6'-acetate
Parishin E
3-Hydroxysebacic acid
Elaidic acid
2-Propylglutaric acid
LPC 18:1
(±)13-HODE
Esculin
Succinic acid
Dihydroroseoside
D-(+)-Malic acid
DL-Malic acid
12-Oxo phytodienoic acid
N-Acetyl-L-phenylalanine
(±)9-HpODE
-600 -400 -200 0
z-score
CK
T2

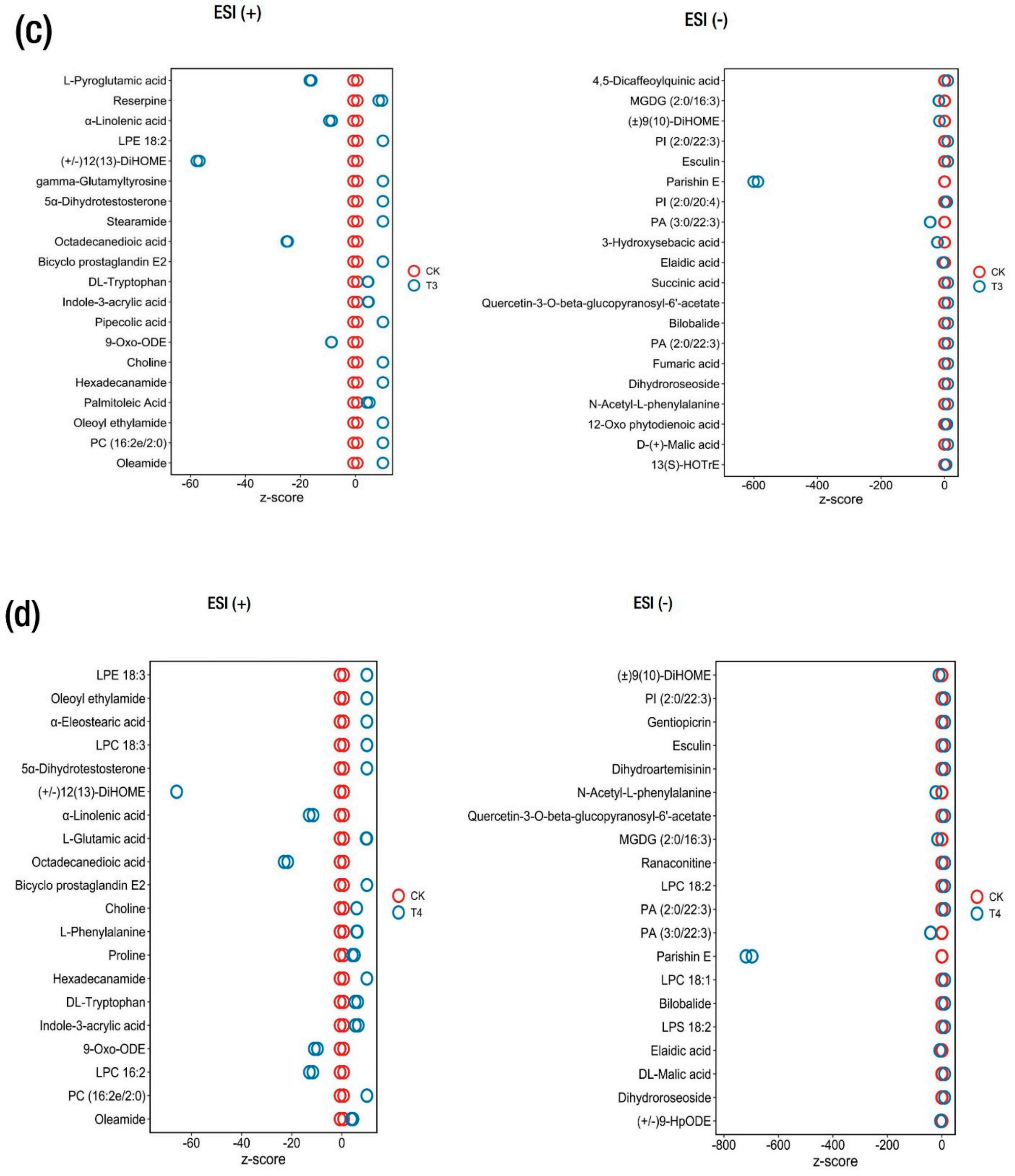

(c)
ESI (+)
L-Pyroglutamic acid
Reserpine
α-Linolenic acid
LPE 18:2
(+/-)12(13)-DiHOME
gamma-Glutamyltyrosine
5α-Dihydrotestosterone
Stearamide
Octadecanedioic acid
Bicyclo prostaglandin E2
DL-Tryptophan
Indole-3-acrylic acid
Pipecolic acid
9-Oxo-ODE
Choline
Hexadecanamide
Palmitoleic Acid
Oleoyl ethylamide
PC (16:2e/2:0)
Oleamide
-60 -40 -20 0
z-score
CK
T3
ESI (-)
4,5-Dicaffeoylquinic acid
MGDG (2:0/16:3)
(±)9(10)-DiHOME
PI (2:0/22:3)
Esculin
Parishin E
PI (2:0/20:4)
PA (3:0/22:3)
3-Hydroxysebacic acid
Elaidic acid
Succinic acid
Quercetin-3-O-beta-glucopyranosyl-6'-acetate
Bilobalide
PA (2:0/22:3)
Fumaric acid
Dihydroroseoside
N-Acetyl-L-phenylalanine
12-Oxo phytodienoic acid
D-(+)-Malic acid
13(S)-HOTrE
-600 -400 -200 0
z-score
CK
T3
(d)
ESI (+)
LPE 18:3
Oleoyl ethylamide
α-Eleostearic acid
LPC 18:3
5α-Dihydrotestosterone
(+/-)12(13)-DiHOME
α-Linolenic acid
L-Glutamic acid
Octadecanedioic acid
Bicyclo prostaglandin E2
Choline
L-Phenylalanine
Proline
Hexadecanamide
DL-Tryptophan
Indole-3-acrylic acid
9-Oxo-ODE
LPC 16:2
PC (16:2e/2:0)
Oleamide
-60 -40 -20 0
z-score
CK
T4
ESI (-)
(±)9(10)-DiHOME
PI (2:0/22:3)
Gentiopicrin
Esculin
Dihydroartemisinin
N-Acetyl-L-phenylalanine
Quercetin-3-O-beta-glucopyranosyl-6'-acetate
MGDG (2:0/16:3)
Ranaconitine
LPC 18:2
PA (2:0/22:3)
PA (3:0/22:3)
Parishin E
LPC 18:1
Bilobalide
LPS 18:2
Elaidic acid
DL-Malic acid
Dihydroroseoside
(+/-)9-HpODE
-800 -600 -400 -200 0
z-score
CK
T4

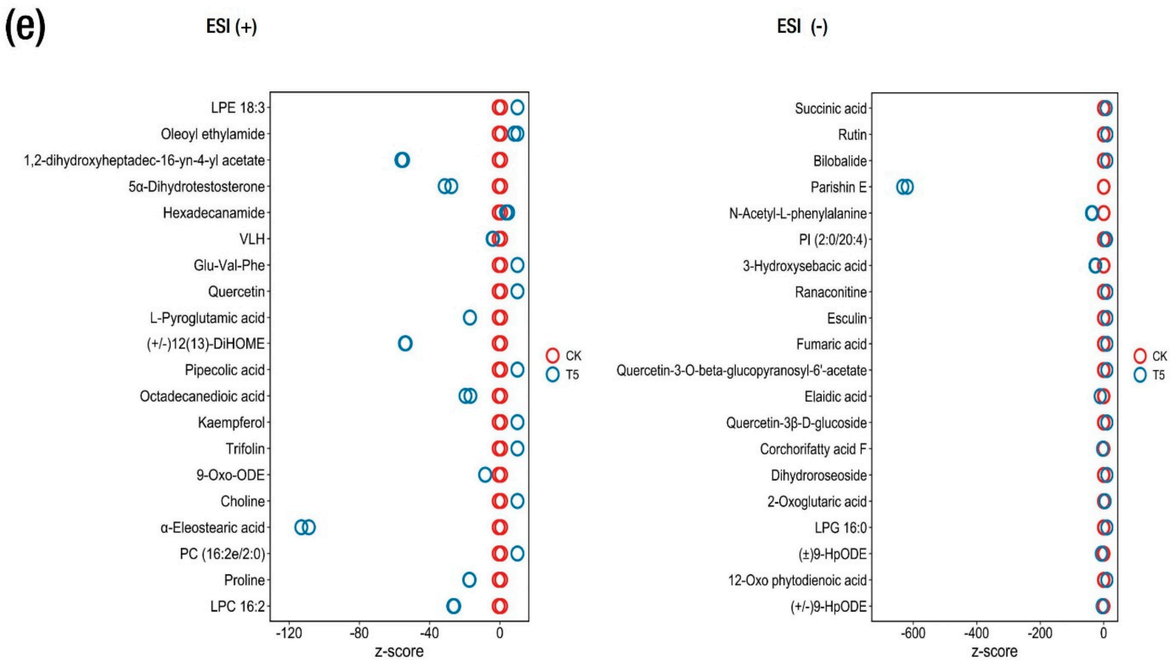

Figure S7. Differential metabolite Z-score plot from various Mg treatment in mulberry. (a) CK-T1 (b) CK-T2 (c) CK-T3 (d) CK-T4 (e) CK-T5. ESI (+); positive ion mode, ESI (-); negative ion mode. The abundance of metabolites after the abscissa is z-score, each ordinate is a metabolite, red represents the control group sample in the comparison group, blue represents the sample of the treatment group, each circle represents a metabolite, each sample z-score<0 represents the downward regulation of metabolites, and z-score>0 represents the upward regulation of metabolite abundance.

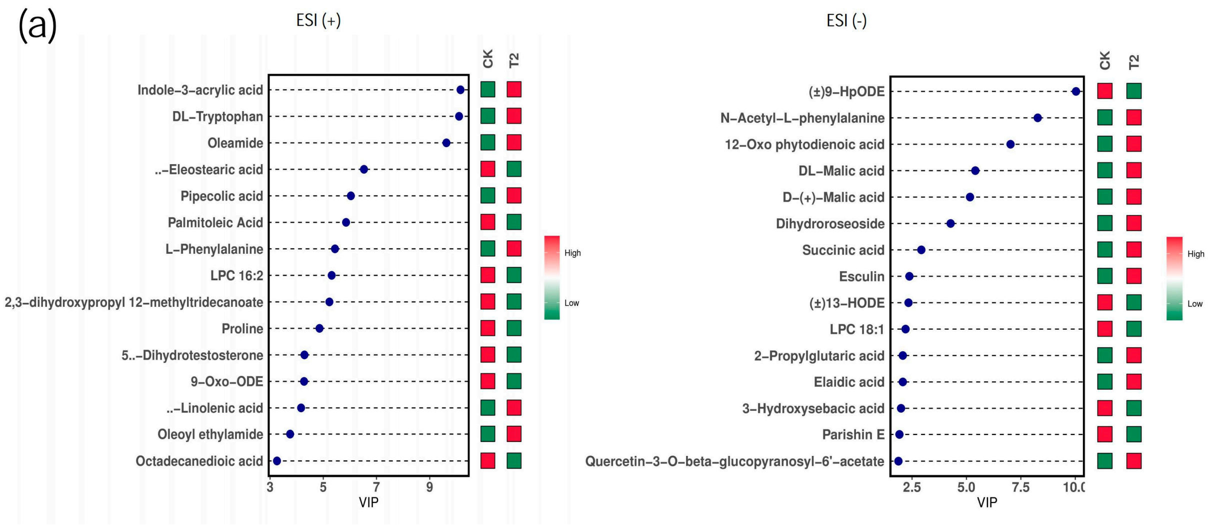

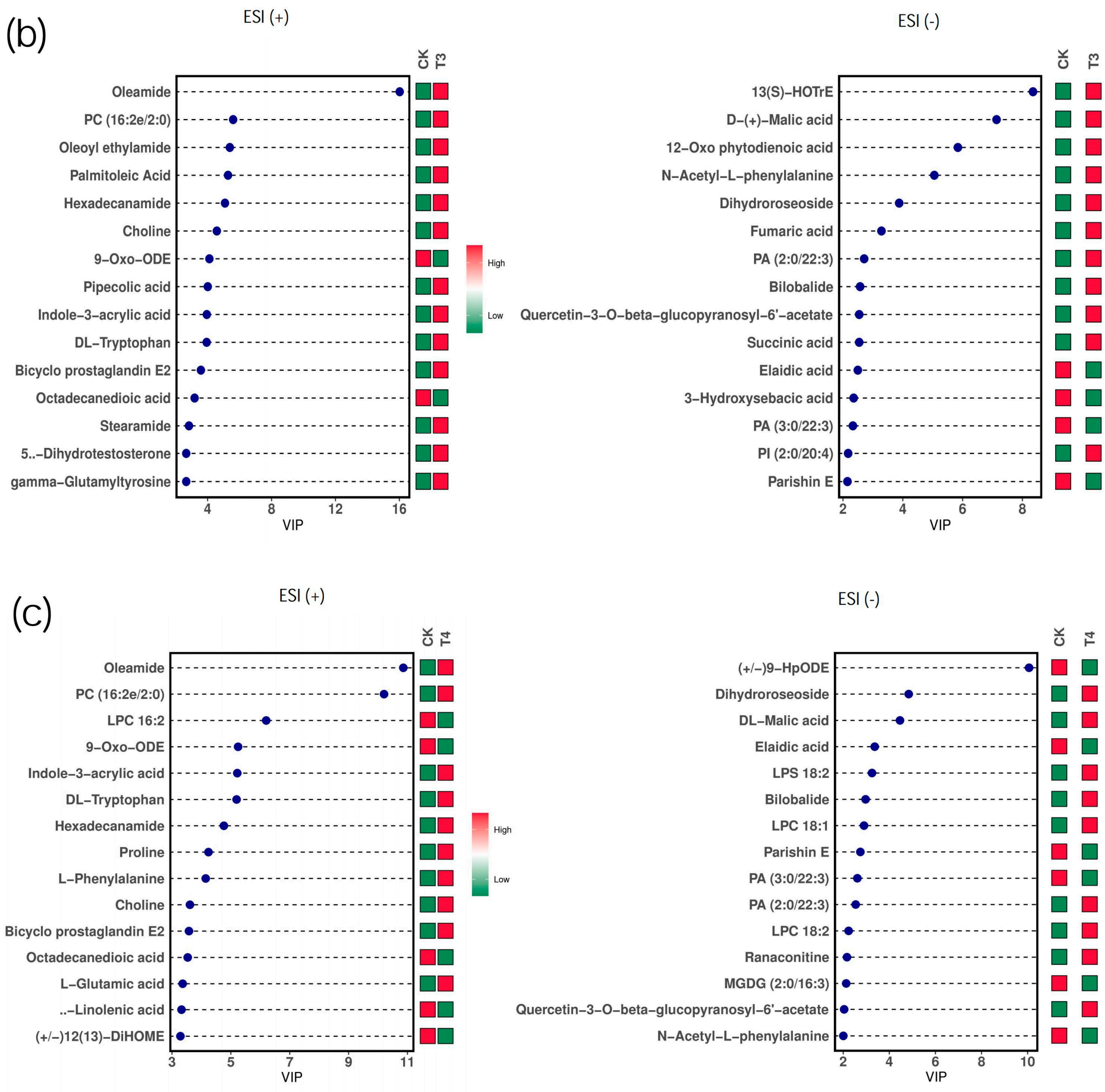

Figure S8. Orthogonal partial least-squares-discriminant analysis (OPLS-DA) scores of the metabolites (a) CK-T2 (b) CK-T3 (c) CK-T4. The OPLS-DA models are R2X, R2Y and Q2. Where R2X and R2Y represent the interpretation rate of the built model to X and Y matrices, respectively, Q2 represents the prediction ability of the model, and the closer these three indicators are to 1, the more stable and reliable the model. Q2>0.5 indicates that the model has good prediction ability, and Q2 >0.9 indicates that the model is excellent.

(a)

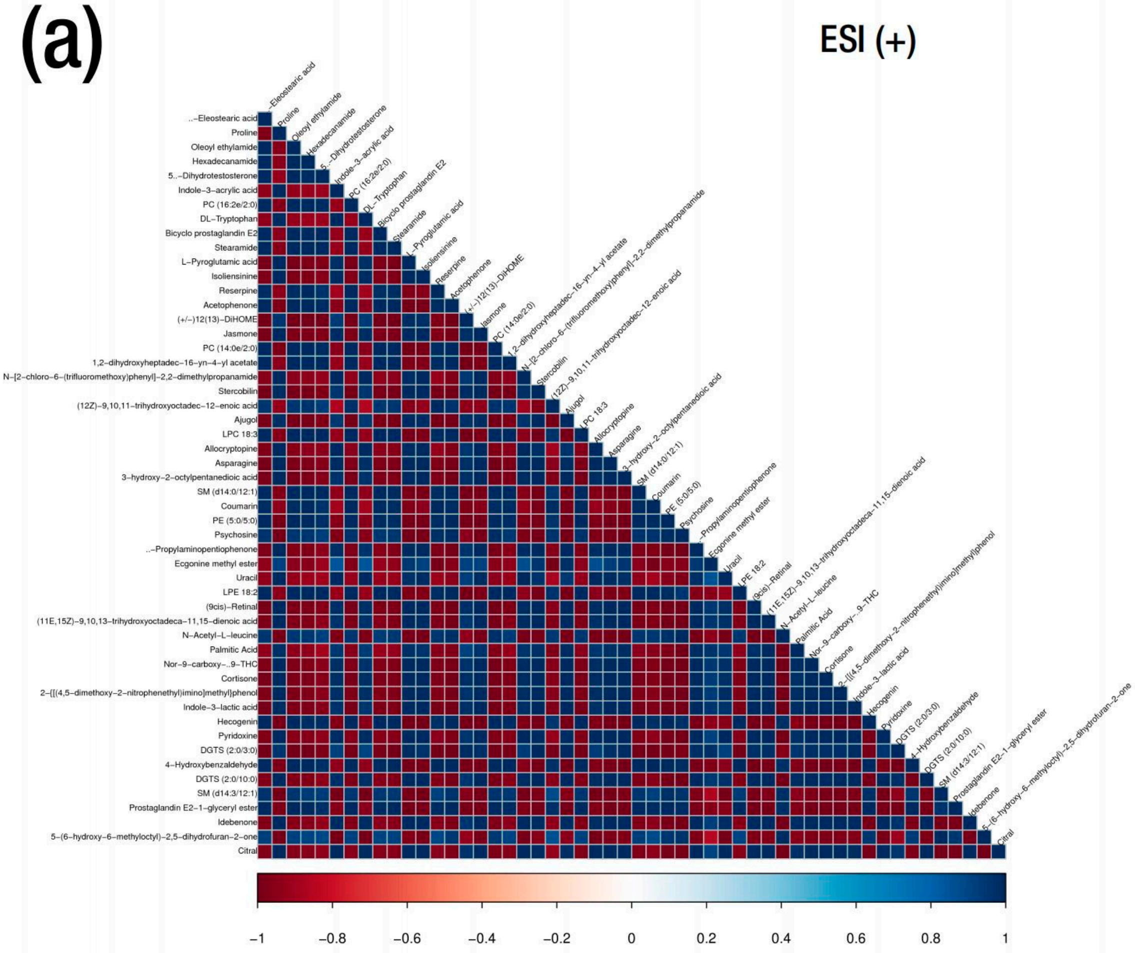
ESI (+)
..–Eleostearic acid
Proline
Oleoyl ethylamide
Hexadecanamide
5..–Dihydrotestosterone
Indole–3–acrylic acid
PC (16:2e/2:0)
DL–Tryptophan
Bicyclo prostaglandin E2
Stearamide
L–Pyroglutamic acid
Isoliensinine
Reserpine
Acetophenone
(+/–)12(13)–DiHOME
Jasmone
PC (14:0e/2:0)
1,2–dihydroxyheptadec–16–yn–4–yl acetate
N–[2–chloro–6–(trifluoromethoxy)phenyl]–2,2–dimethylpropanamide
Stercobilin
(12Z)–9,10,11–trihydroxyoctadec–12–enoic acid
Ajugol
LPC 18:3
Allocryptopine
Asparagine
3–hydroxy–2–octylpentanedioic acid
SM (d14:0/12:1)
Coumarin
PE (5:0/5:0)
Psychosine
..–Propylaminopentiophenone
Ecgonine methyl ester
Uracil
LPE 18:2
(9cis)–Retinal
(11E,15Z)–9,10,13–trihydroxyoctadeca–11,15–dienoic acid
N–Acetyl–L–leucine
Palmitic Acid
Nor–9–carboxy–..9–THC
Cortisone
2–{[(4,5–dimethoxy–2–nitrophenethyl)imino]methyl}phenol
Indole–3–lactic acid
Hecogenin
Pyridoxine
DGTS (2:0/3:0)
4–Hydroxybenzaldehyde
DGTS (2:0/10:0)
SM (d14:3/12:1)
Prostaglandin E2–1–glyceryl ester
Idebenone
5–(6–hydroxy–6–methyloctyl)–2,5–dihydrofuran–2–one
Citral
–1
–0.8
–0.6
–0.4
–0.2
0
0.2
0.4
0.6
0.8
1

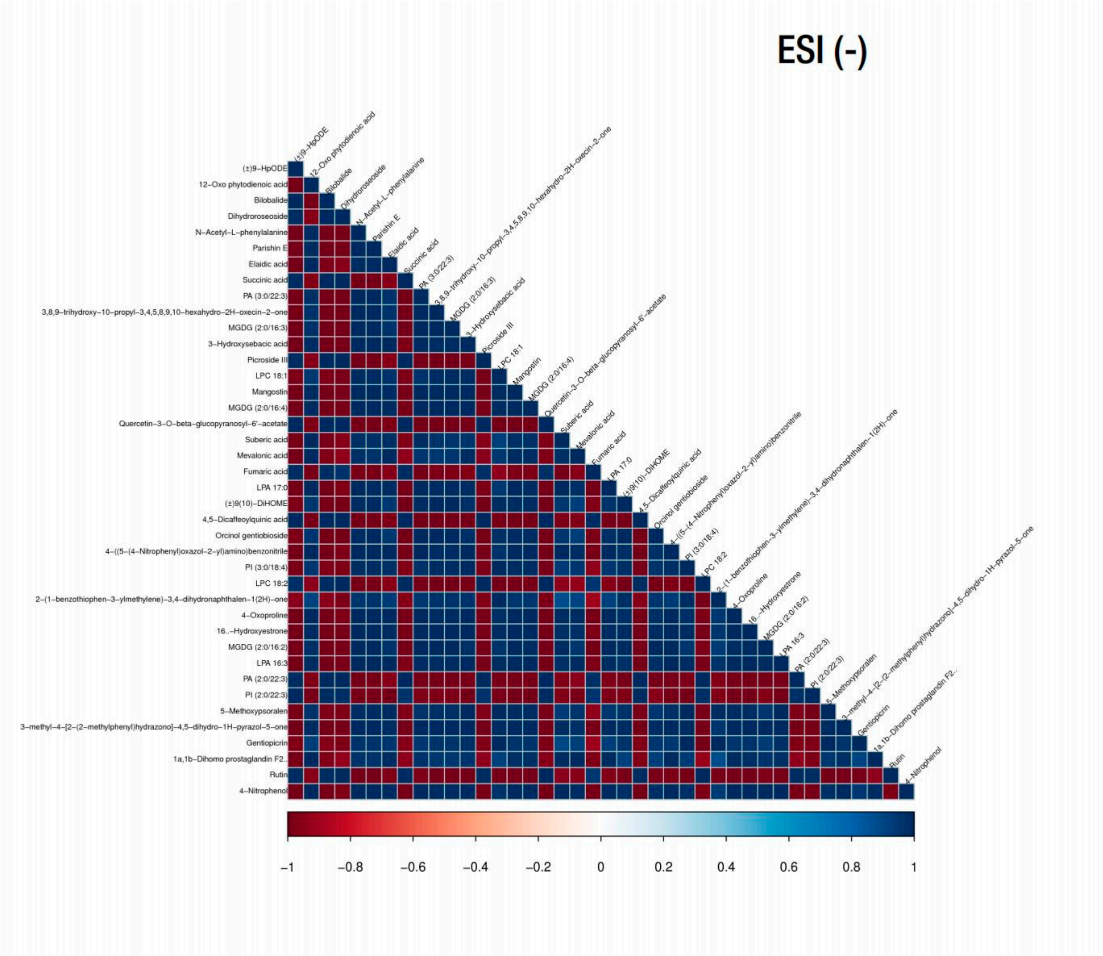

ESI (-)
(±)9–HpODE
12–Oxo phytodienoic acid
Bilobalide
Dihydroroseoside
N–Acetyl–L–phenylalanine
Parishin E
Elaidic acid
Succinic acid
PA (3:0/22:3)
3,8,9–trihydroxy–10–propyl–3,4,5,8,9,10–hexahydro–2H–oxecin–2–one
MGDG (2:0/16:3)
3–Hydroxysebacic acid
Picroside III
LPC 18:1
Mangostin
MGDG (2:0/16:4)
Quercetin–3–O–beta–glucopyranosyl–6'–acetate
Suberic acid
Mevalonic acid
Fumaric acid
LPA 17:0
(±)9(10)–DiHOME
4,5–Dicaffeoylquinic acid
Orcinol gentiobioside
4–((5–(4–Nitrophenyl)oxazol–2–yl)amino)benzonitrile
PI (3:0/18:4)
LPC 18:2
2–(1–benzothiophen–3–ylmethylene)–3,4–dihydronaphthalen–1(2H)–one
4–Oxoproline
16..–Hydroxyestrone
MGDG (2:0/16:2)
LPA 16:3
PA (2:0/22:3)
PI (2:0/22:3)
5–Methoxypsoralen
3–methyl–4–[2–(2–methylphenyl)hydrazono]–4,5–dihydro–1H–pyrazol–5–one
Gentiopicrin
1a,1b–Dihomo prostaglandin F2..
Rutin
4–Nitrophenol
–1
–0.8
–0.6
–0.4
–0.2
0
0.2
0.4
0.6
0.8
1

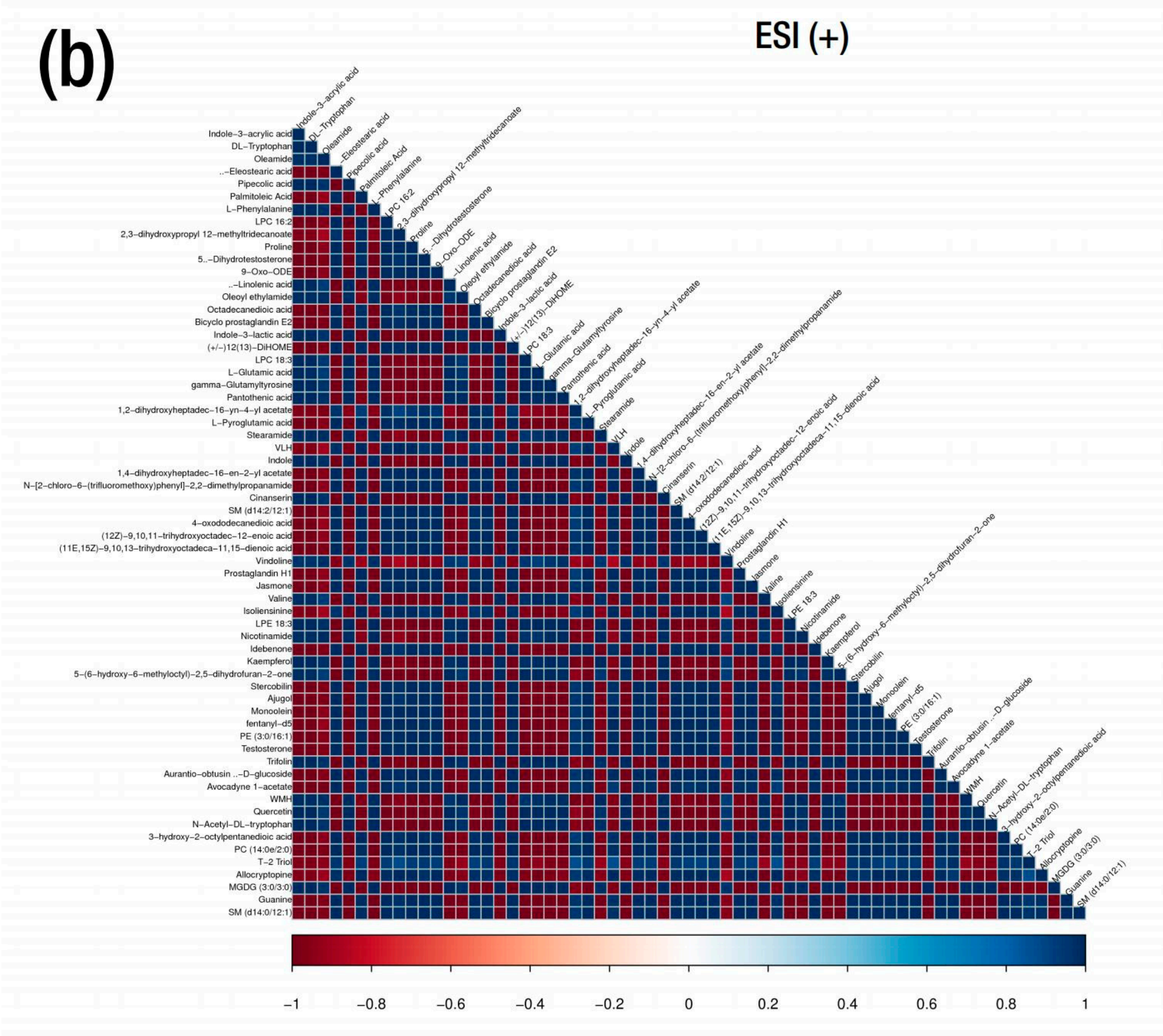
(b)
ESI (+)
Indole−3−acrylic acid
DL−Tryptophan
Oleamide
..−Eleostearic acid
Pipecolic acid
Palmitoleic Acid
L−Phenylalanine
LPC 16:2
2,3−dihydroxypropyl 12−methyltridecanoate
Proline
5..−Dihydrotestosterone
9−Oxo−ODE
..−Linolenic acid
Oleoyl ethylamide
Octadecanedioic acid
Bicyclo prostaglandin E2
Indole−3−lactic acid
(+/−)12(13)−DiHOME
LPC 18:3
L−Glutamic acid
gamma−Glutamyltyrosine
Pantothenic acid
1,2−dihydroxyheptadec−16−yn−4−yl acetate
L−Pyroglutamic acid
Stearamide
VLH
Indole
1,4−dihydroxyheptadec−16−en−2−yl acetate
N−[2−chloro−6−(trifluoromethoxy)phenyl]−2,2−dimethylpropanamide
Cinanserin
SM (d14:2/12:1)
4−oxododecanedioic acid
(12Z)−9,10,11−trihydroxyoctadec−12−enoic acid
(11E,15Z)−9,10,13−trihydroxyoctadeca−11,15−dienoic acid
Vindoline
Prostaglandin H1
Jasmone
Valine
Isoliensinine
LPE 18:3
Nicotinamide
Idebenone
Kaempferol
5−(6−hydroxy−6−methyloctyl)−2,5−dihydrofuran−2−one
Stercobilin
Ajugol
Monoolein
fentanyl−d5
PE (3:0/16:1)
Testosterone
Trifolin
Aurantio−obtusin ..−D−glucoside
Avocadyne 1−acetate
WMH
Quercetin
N−Acetyl−DL−tryptophan
3−hydroxy−2−octylpentanedioic acid
PC (14:0e/2:0)
T−2 Triol
Allocryptopine
MGDG (3:0/3:0)
Guanine
SM (d14:0/12:1)
−1
−0.8
−0.6
−0.4
−0.2
0
0.2
0.4
0.6
0.8
1

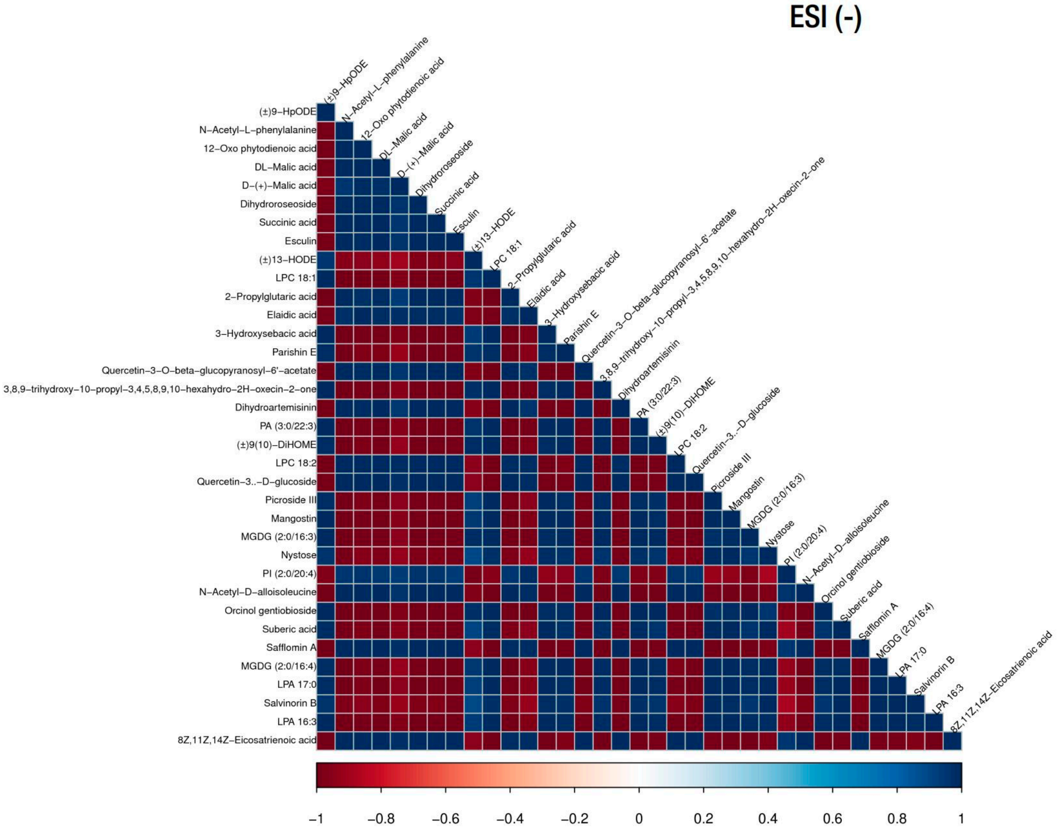
ESI (-)
(±)9−HpODE
N−Acetyl−L−phenylalanine
12−Oxo phytodienoic acid
DL−Malic acid
D−(+)−Malic acid
Dihydroroseoside
Succinic acid
Esculin
(±)13−HODE
LPC 18:1
2−Propylglutaric acid
Elaidic acid
3−Hydroxysebacic acid
Parishin E
Quercetin−3−O−beta−glucopyranosyl−6'−acetate
3,8,9−trihydroxy−10−propyl−3,4,5,8,9,10−hexahydro−2H−oxecin−2−one
Dihydroartemisinin
PA (3:0/22:3)
(±)9(10)−DiHOME
LPC 18:2
Quercetin−3..−D−glucoside
Picroside III
Mangostin
MGDG (2:0/16:3)
Nystose
PI (2:0/20:4)
N−Acetyl−D−alloisoleucine
Orcinol gentiobioside
Suberic acid
Safflomin A
MGDG (2:0/16:4)
LPA 17:0
Salvinorin B
LPA 16:3
8Z,11Z,14Z−Eicosatrienoic acid
−1
−0.8
−0.6
−0.4
−0.2
0
0.2
0.4
0.6
0.8
1

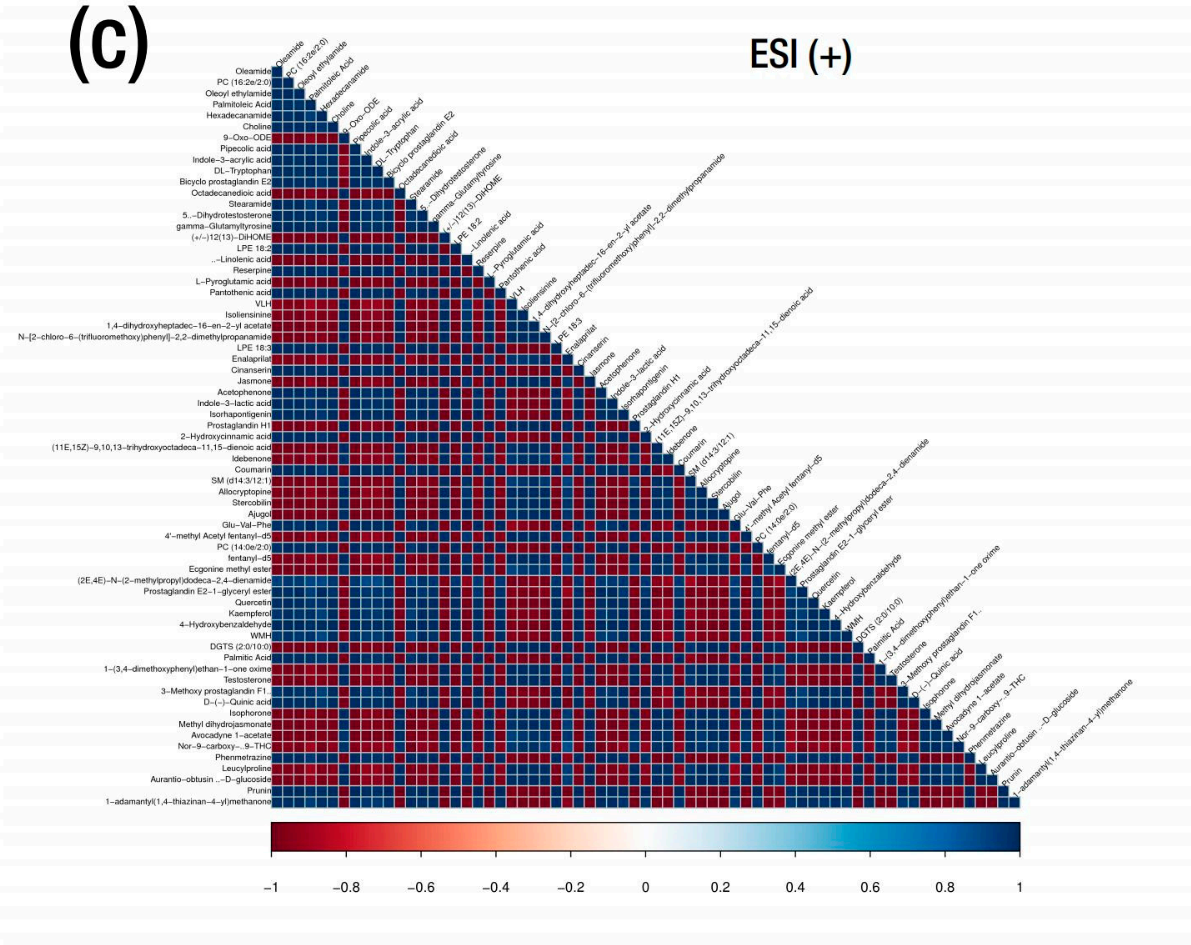
(c)
ESI (+)
Oleamide
PC (16:2e/2:0)
Oleoyl ethylamide
Palmitoleic Acid
Hexadecanamide
Choline
9–Oxo–ODE
Pipecolic acid
Indole–3–acrylic acid
DL–Tryptophan
Bicyclo prostaglandin E2
Octadecanedioic acid
Stearamide
5..–Dihydrotestosterone
gamma–Glutamyltyrosine
(+/–)12(13)–DiHOME
LPE 18:2
..–Linolenic acid
Reserpine
L–Pyroglutamic acid
Pantothenic acid
VLH
Isoliensinine
1,4–dihydroxyheptadec–16–en–2–yl acetate
N–[2–chloro–6–(trifluoromethoxy)phenyl]–2,2–dimethylpropanamide
LPE 18:3
Enalaprilat
Cinanserin
Jasmone
Acetophenone
Indole–3–lactic acid
Isorhapontigenin
Prostaglandin H1
2–Hydroxycinnamic acid
(11E,15Z)–9,10,13–trihydroxyoctadeca–11,15–dienoic acid
Idebenone
Coumarin
SM (d14:3/12:1)
Allocryptopine
Stercobilin
Ajugol
Glu–Val–Phe
4'–methyl Acetyl fentanyl–d5
PC (14:0e/2:0)
fentanyl–d5
Ecgonine methyl ester
(2E,4E)–N–(2–methylpropyl)dodeca–2,4–dienamide
Prostaglandin E2–1–glyceryl ester
Quercetin
Kaempferol
4–Hydroxybenzaldehyde
WMH
DGTS (2:0/10:0)
Palmitic Acid
1–(3,4–dimethoxyphenyl)ethan–1–one oxime
Testosterone
3–Methoxy prostaglandin F1..
D–(–)–Quinic acid
Isophorone
Methyl dihydrojasmonate
Avocadyne 1–acetate
Nor–9–carboxy–..9–THC
Phenmetrazine
Leucylproline
Aurantio–obtusin ...–D–glucoside
Prunin
1–adamantyl(1,4–thiazinan–4–yl)methanone
–1
–0.8
–0.6
–0.4
–0.2
0
0.2
0.4
0.6
0.8
1

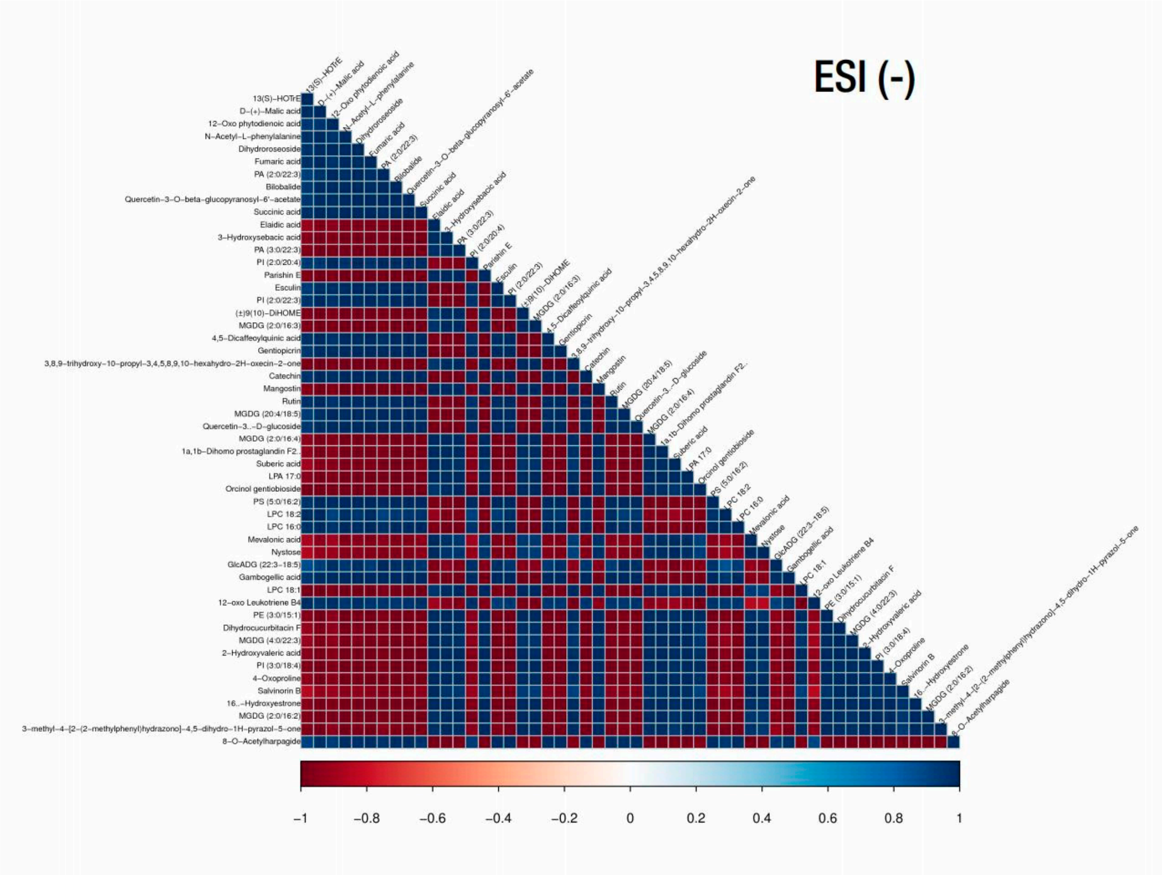

ESI (-)
13(S)-HOTrE
D-(+)-Malic acid
12-Oxo phytodienoic acid
N-Acetyl-L-phenylalanine
Dihydroroseoside
Fumaric acid
PA (2:0/22:3)
Bilobalide
Quercetin-3-O-beta-glucopyranosyl-6'-acetate
Succinic acid
Elaidic acid
3-Hydroxysebacic acid
PA (3:0/22:3)
PI (2:0/20:4)
Parishin E
Esculin
PI (2:0/22:3)
(±)9(10)-DiHOME
MGDG (2:0/16:3)
4,5-Dicaffeoylquinic acid
Gentiopicrin
3,8,9-trihydroxy-10-propyl-3,4,5,8,9,10-hexahydro-2H-oxecin-2-one
Catechin
Mangostin
Rutin
MGDG (20:4/18:5)
Quercetin-3..-D-glucoside
MGDG (2:0/16:4)
1a,1b-Dihomo prostaglandin F2..
Suberic acid
LPA 17:0
Orcinol gentiobioside
PS (5:0/16:2)
LPC 18:2
LPC 16:0
Mevalonic acid
Nystose
GlcADG (22:3-18:5)
Gambogellic acid
LPC 18:1
12-oxo Leukotriene B4
PE (3:0/15:1)
Dihydrocucurbitacin F
MGDG (4:0/22:3)
2-Hydroxyvaleric acid
PI (3:0/18:4)
4-Oxoproline
Salvinorin B
16..-Hydroxyestrone
MGDG (2:0/16:2)
3-methyl-4-[2-(2-methylphenyl)hydrazono]-4,5-dihydro-1H-pyrazol-5-one
8-O-Acetylharpagide
-1
-0.8
-0.6
-0.4
-0.2
0
0.2
0.4
0.6
0.8
1

(d)

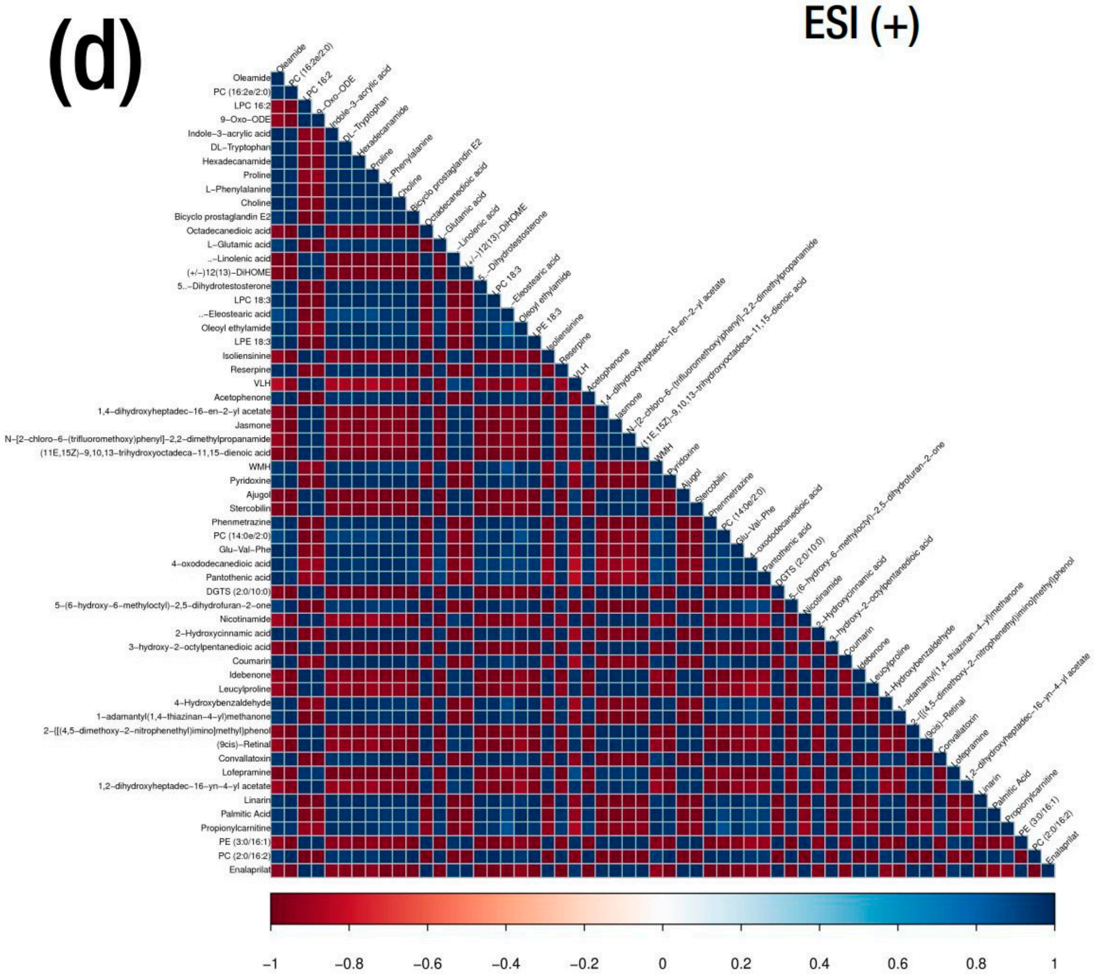

ESI (+)
Oleamide
PC (16:2e/2:0)
LPC 16:2
9–Oxo–ODE
Indole–3–acrylic acid
DL–Tryptophan
Hexadecanamide
Proline
L–Phenylalanine
Choline
Bicyclo prostaglandin E2
Octadecanedioic acid
L–Glutamic acid
..–Linolenic acid
(+/–)12(13)–DiHOME
5..–Dihydrotestosterone
LPC 18:3
..–Eleostearic acid
Oleoyl ethylamide
LPE 18:3
Isoliensinine
Reserpine
VLH
Acetophenone
1,4–dihydroxyheptadec–16–en–2–yl acetate
Jasmone
N–[2–chloro–6–(trifluoromethoxy)phenyl]–2,2–dimethylpropanamide
(11E,15Z)–9,10,13–trihydroxyoctadeca–11,15–dienoic acid
WMH
Pyridoxine
Ajugol
Stercobilin
Phenmetrazine
PC (14:0e/2:0)
Glu–Val–Phe
4–oxododecanedioic acid
Pantothenic acid
DGTS (2:0/10:0)
5–(6–hydroxy–6–methyloctyl)–2,5–dihydrofuran–2–one
Nicotinamide
2–Hydroxycinnamic acid
3–hydroxy–2–octylpentanedioic acid
Coumarin
Idebenone
Leucylproline
4–Hydroxybenzaldehyde
1–adamantyl(1,4–thiazinan–4–yl)methanone
2–{[(4,5–dimethoxy–2–nitrophenethyl)imino]methyl}phenol
(9cis)–Retinal
Convallatoxin
Lofepramine
1,2–dihydroxyheptadec–16–yn–4–yl acetate
Linarin
Palmitic Acid
Propionylcarnitine
PE (3:0/16:1)
PC (2:0/16:2)
Enalaprilat
–1
–0.8
–0.6
–0.4
–0.2
0
0.2
0.4
0.6
0.8
1

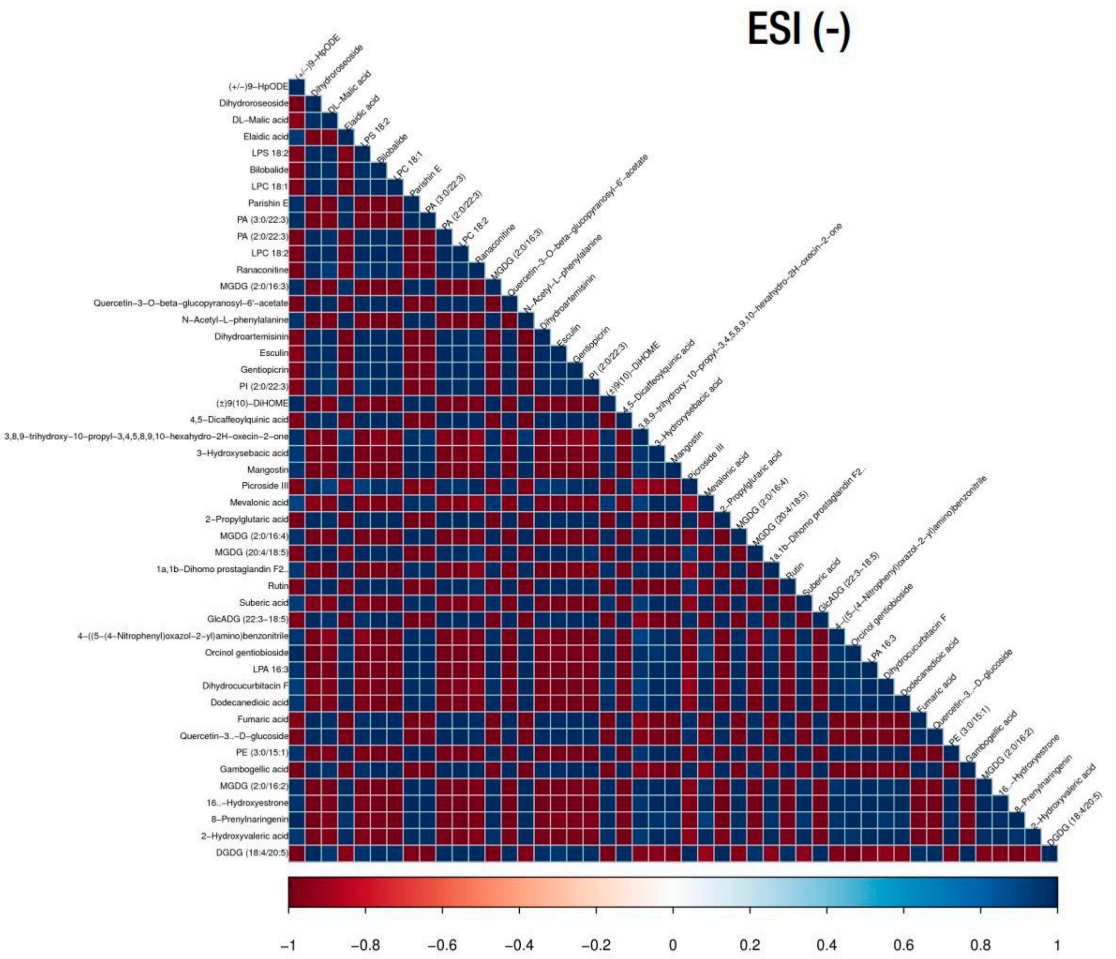
ESI (-)
(+/−)9−HpODE
Dihydroroseoside
DL−Malic acid
Elaidic acid
LPS 18:2
Bilobalide
LPC 18:1
Parishin E
PA (3:0/22:3)
PA (2:0/22:3)
LPC 18:2
Ranaconitine
MGDG (2:0/16:3)
Quercetin−3−O−beta−glucopyranosyl−6'−acetate
N−Acetyl−L−phenylalanine
Dihydroartemisinin
Esculin
Gentiopicrin
PI (2:0/22:3)
(±)9(10)−DiHOME
4,5−Dicaffeoylquinic acid
3,8,9−trihydroxy−10−propyl−3,4,5,8,9,10−hexahydro−2H−oxecin−2−one
3−Hydroxysebacic acid
Mangostin
Picroside III
Mevalonic acid
2−Propylglutaric acid
MGDG (2:0/16:4)
MGDG (20:4/18:5)
1a,1b−Dihomo prostaglandin F2..
Rutin
Suberic acid
GlcADG (22:3−18:5)
4−((5−(4−Nitrophenyl)oxazol−2−yl)amino)benzonitrile
Orcinol gentiobioside
LPA 16:3
Dihydrocucurbitacin F
Dodecanedioic acid
Fumaric acid
Quercetin−3..−D−glucoside
PE (3:0/15:1)
Gambogellic acid
MGDG (2:0/16:2)
16..−Hydroxyestrone
8−Prenylnaringenin
2−Hydroxyvaleric acid
DGDG (18:4/20:5)
−1
−0.8
−0.6
−0.4
−0.2
0
0.2
0.4
0.6
0.8
1

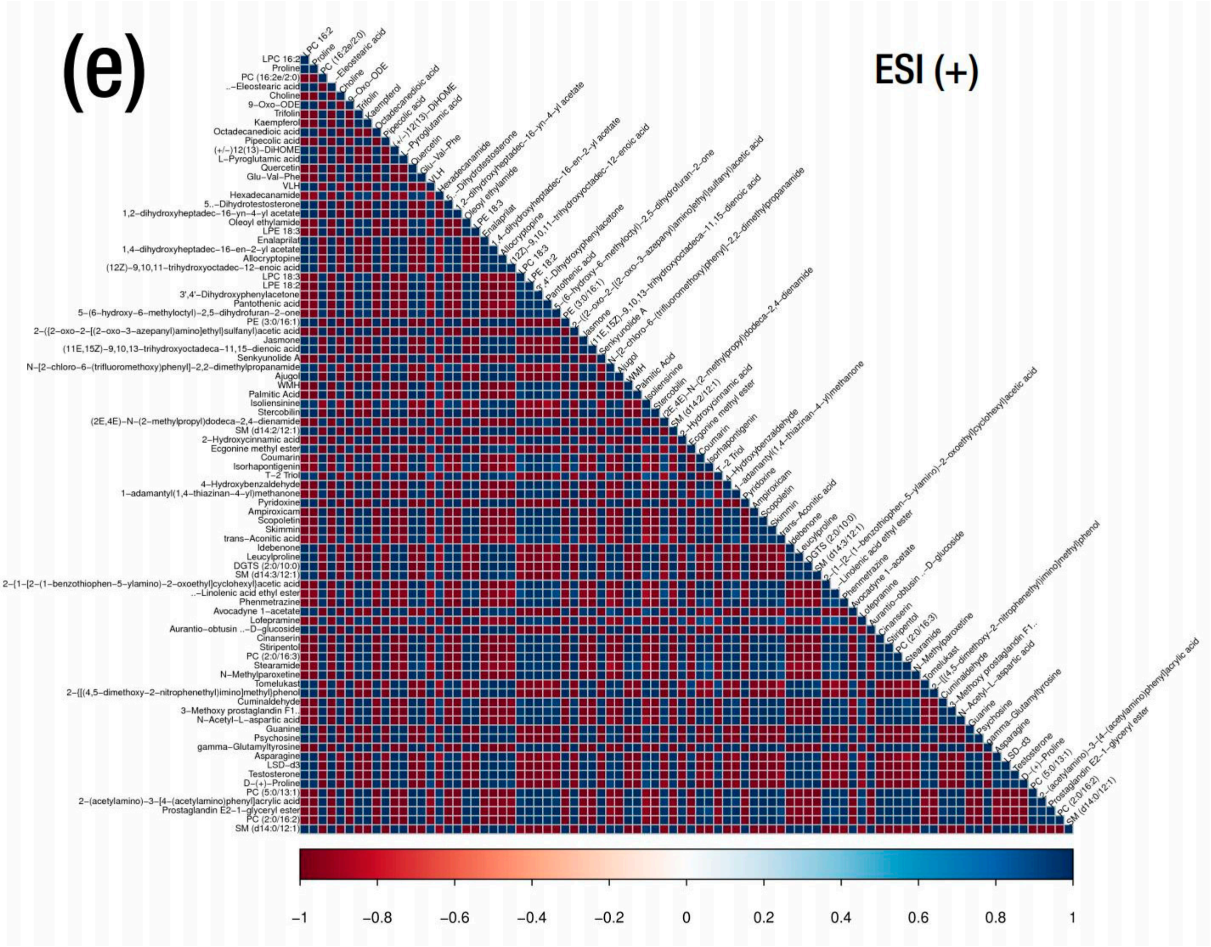

(e)
ESI (+)
LPC 16:2
Proline
PC (16:2e/2:0)
..–Eleostearic acid
Choline
9–Oxo–ODE
Trifolin
Kaempferol
Octadecanedioic acid
Pipecolic acid
(+/–)12(13)–DiHOME
L–Pyroglutamic acid
Quercetin
Glu–Val–Phe
VLH
Hexadecanamide
5..–Dihydrotestosterone
1,2–dihydroxyheptadec–16–yn–4–yl acetate
Oleoyl ethylamide
LPE 18:3
Enalaprilat
1,4–dihydroxyheptadec–16–en–2–yl acetate
Allocryptopine
(12Z)–9,10,11–trihydroxyoctadec–12–enoic acid
LPC 18:3
LPE 18:2
3',4'–Dihydroxyphenylacetone
Pantothenic acid
5–(6–hydroxy–6–methyloctyl)–2,5–dihydrofuran–2–one
PE (3:0/16:1)
2–({2–oxo–2–[(2–oxo–3–azepanyl)amino]ethyl}sulfanyl)acetic acid
Jasmone
(11E,15Z)–9,10,13–trihydroxyoctadeca–11,15–dienoic acid
Senkyunolide A
N–[2–chloro–6–(trifluoromethoxy)phenyl]–2,2–dimethylpropanamide
Ajugol
WMH
Palmitic Acid
Isoliensinine
Stercobilin
(2E,4E)–N–(2–methylpropyl)dodeca–2,4–dienamide
SM (d14:2/12:1)
2–Hydroxycinnamic acid
Ecgonine methyl ester
Coumarin
Isorhapontigenin
T–2 Triol
4–Hydroxybenzaldehyde
1–adamantyl(1,4–thiazinan–4–yl)methanone
Pyridoxine
Ampiroxicam
Scopoletin
Skimmin
trans–Aconitic acid
Idebenone
Leucylproline
DGTS (2:0/10:0)
SM (d14:3/12:1)
2–[1–[2–(1–benzothiophen–5–ylamino)–2–oxoethyl]cyclohexyl]acetic acid
..–Linolenic acid ethyl ester
Phenmetrazine
Avocadyne 1–acetate
Lofepramine
Aurantio–obtusin ..–D–glucoside
Cinanserin
Stiripentol
PC (2:0/16:3)
Stearamide
N–Methylparoxetine
Tomelukast
2–{[(4,5–dimethoxy–2–nitrophenethyl)imino]methyl}phenol
Cuminaldehyde
3–Methoxy prostaglandin F1..
N–Acetyl–L–aspartic acid
Guanine
Psychosine
gamma–Glutamyltyrosine
Asparagine
LSD–d3
Testosterone
D–(+)–Proline
PC (5:0/13:1)
2–(acetylamino)–3–[4–(acetylamino)phenyl]acrylic acid
Prostaglandin E2–1–glyceryl ester
PC (2:0/16:2)
SM (d14:0/12:1)
–1
–0.8
–0.6
–0.4
–0.2
0
0.2
0.4
0.6
0.8
1

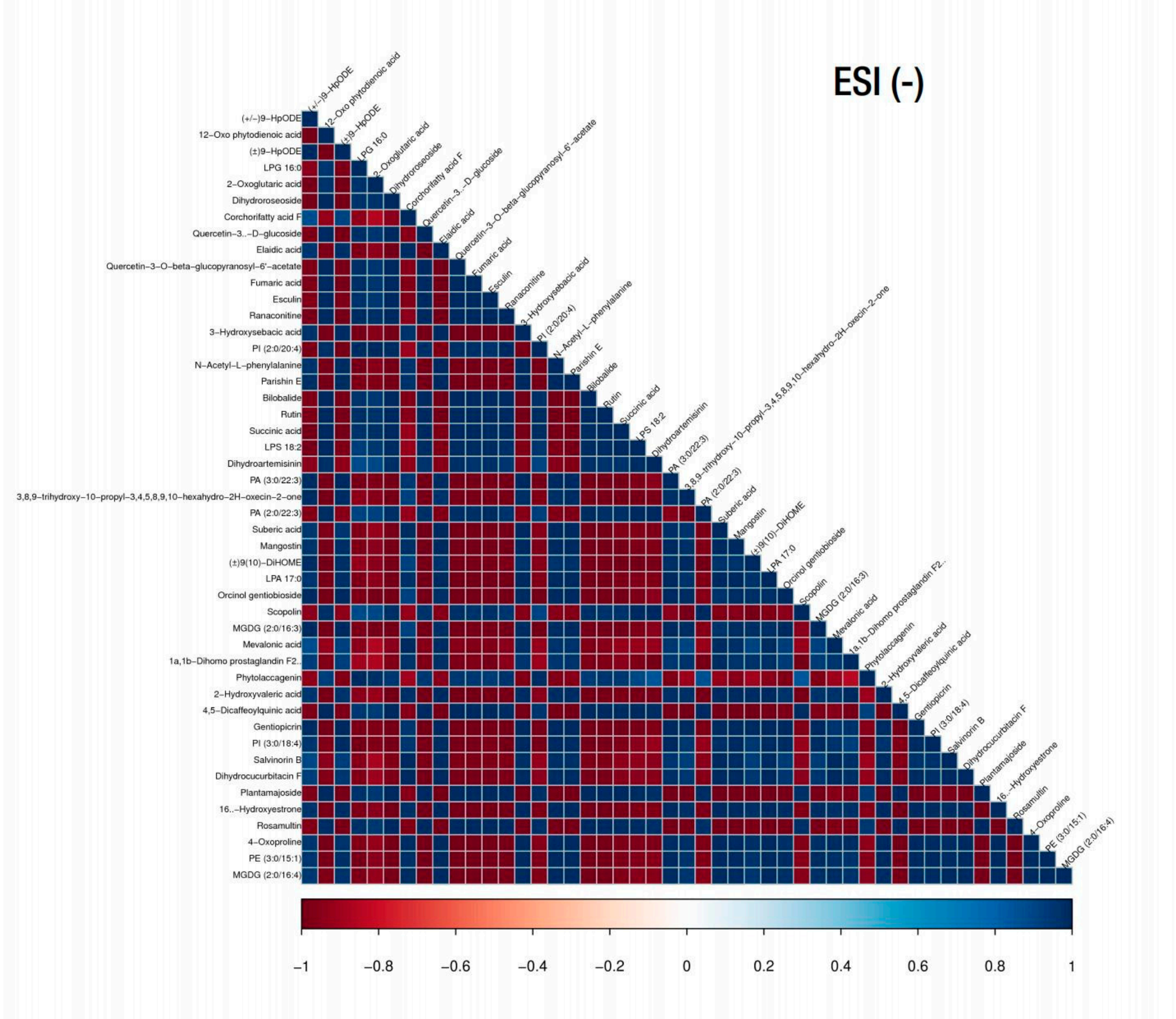

Figure S9 Sample correlation heatmap chart description. (a) CK-T1 (b) CK-T2 (c) CK-T3 (d) CK-T4 (e) CK-T5. Red color represents negative correlation and blue color represent positive correlation Each row and column in the graph represent metabolite, and the value in each cell represents the Pearson correlation coefficient of the corresponding two metabolites, the higher the value, the darker the color, indicating that the two samples are more correlated.

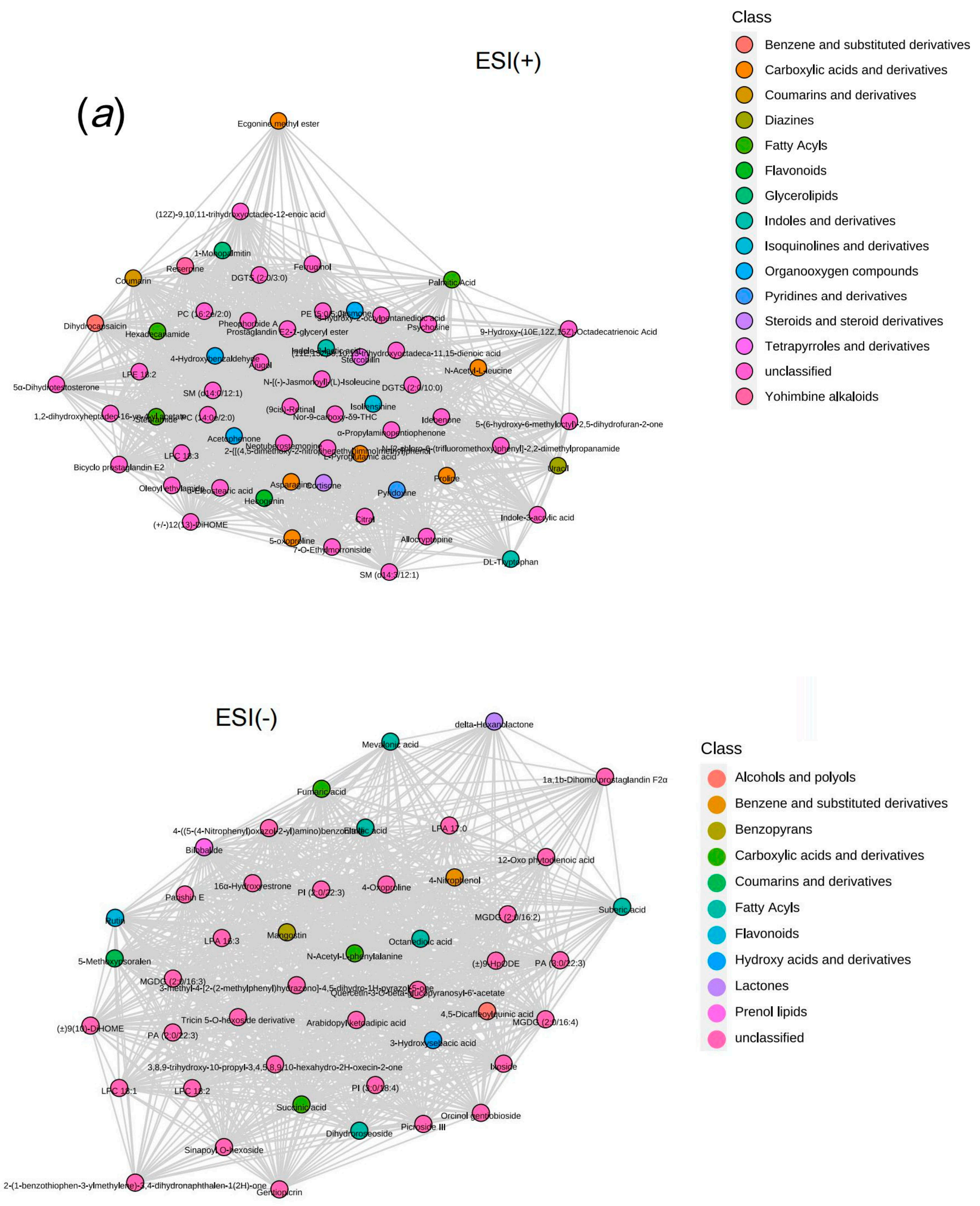

(a)
ESI(+)
Class
Benzene and substituted derivatives
Carboxylic acids and derivatives
Coumarins and derivatives
Diazines
Fatty Acyls
Flavonoids
Glycerolipids
Indoles and derivatives
Isoquinolines and derivatives
Organooxygen compounds
Pyridines and derivatives
Steroids and steroid derivatives
Tetrapyrroles and derivatives
unclassified
Yohimbine alkaloids
ESI(-)
Class
Alcohols and polyols
Benzene and substituted derivatives
Benzopyrans
Carboxylic acids and derivatives
Coumarins and derivatives
Fatty Acyls
Flavonoids
Hydroxy acids and derivatives
Lactones
Prenol lipids
unclassified

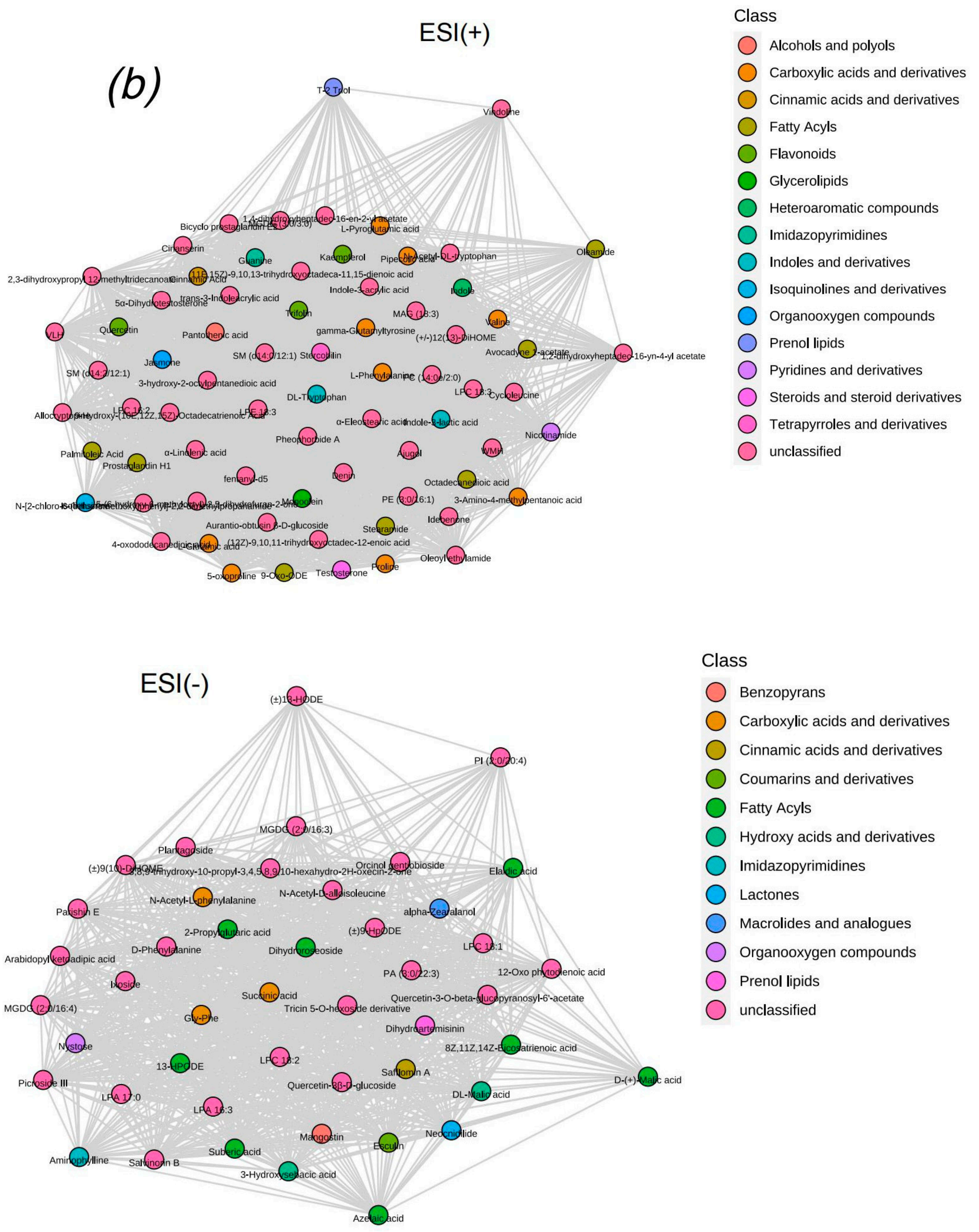
(b)
ESI(+)
Class
Alcohols and polyols
Carboxylic acids and derivatives
Cinnamic acids and derivatives
Fatty Acyls
Flavonoids
Glycerolipids
Heteroaromatic compounds
Imidazopyrimidines
Indoles and derivatives
Isoquinolines and derivatives
Organooxygen compounds
Prenol lipids
Pyridines and derivatives
Steroids and steroid derivatives
Tetrapyrroles and derivatives
unclassified
T-2 Triol
Vindoline
Oleamide
L-Pyroglutamic acid
Cirlanserin
Guanine
Kaempferol
Indole-3-acrylic acid
Indole
Trifolin
MAG (18:3)
Valine
Quercetin
Pantothenic acid
gamma-Glutamyltyrosine
(+/-)12(13)-DiHOME
VLH
Jasmone
SM (d14:2/12:1)
3-hydroxy-2-octylpentanedioic acid
L-Phenylalanine
Cycloleucine
DL-Tryptophan
Nicotinamide
Pheophorbide A
Palmitoleic Acid
Prostaglandin H1
α-Linolenic acid
fenfuryl-d5
Ajugol
WMH
Octadecanedioic acid
3-Amino-4-methylpentanoic acid
Aurantio-obtusin β-D-glucoside
Stearamide
Idebenone
Oleoyl ethylamide
5-oxoproline
9-Oxo-ODE
Testosterone
Proline
ESI(-)
Class
Benzopyrans
Carboxylic acids and derivatives
Cinnamic acids and derivatives
Coumarins and derivatives
Fatty Acyls
Hydroxy acids and derivatives
Imidazopyrimidines
Lactones
Macrolides and analogues
Organooxygen compounds
Prenol lipids
unclassified
(±)13-HODE
PI (20:0/20:4)
MGDG (2:0/16:3)
Plantagoside
Orcinol gentiobioside
Elaidic acid
N-Acetyl-L-phenylalanine
N-Acetyl-D-alloisoleucine
Paulshin E
alpha-Zearalanol
2-Propylglutaric acid
(±)9-HpODE
Arabidopyl ketoadipic acid
D-Phenylalanine
Dihydrorobeoside
LPC 18:1
PA (18:0/22:3)
12-Oxo phytodienoic acid
Succinic acid
Tricin 5-O-hexoside derivative
Quercetin-3-O-beta-glucopyranosyl-6'-acetate
MGDG (2:0/16:4)
Gly-Phe
Dihydroartemisinin
Nystose
8Z,11Z,14Z-Eicosatrienoic acid
13-HpODE
LPC 18:2
Safflomin A
D-(+)-Malic acid
Picroside III
LPA 17:0
Quercetin-3β-D-glucoside
DL-Malic acid
LPA 18:3
Mangostin
Neocnidilide
Esculin
Aminophylline
Salvinorin B
Suberic acid
3-Hydroxysebacic acid
Azelaic acid

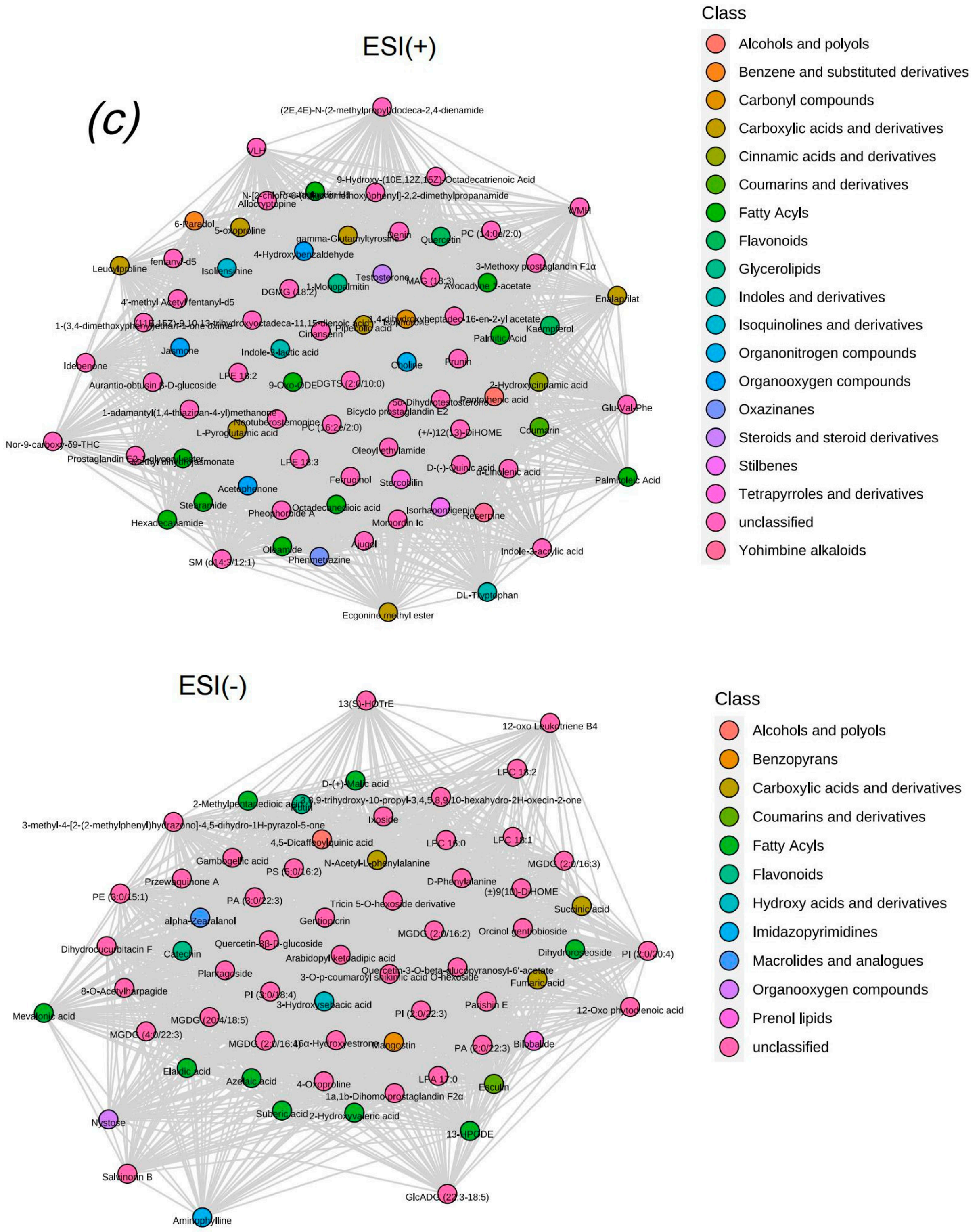

(c)
ESI(+)
(2E,4E)-N-(2-methylpropyl)dodeca-2,4-dienamide
9-Hydroxy-(10E,12Z,15Z)-Octadecatrienoic Acid
6-Paradol
5-oxoproline
gamma-Glutamyltyrosine
Quercetin
PC (14:0e/2:0)
4-Hydroxybenzaldehyde
Leucylproline
fentanyl-d5
Isoliensinine
3-Methoxy prostaglandin F1α
Testosterone
MAG (18:3)
Avocadyne 1-acetate
Enalaprilat
DGMG (18:2)
4'-methyl Acetyl fentanyl-d5
Cinanserin
Palmitic Acid
Kaempferol
Jasmone
Indole-3-lactic acid
Choline
Prunin
Idebenone
LPE 18:2
Aurantio-obtusin β-D-glucoside
2-Hydroxycinnamic acid
Glu-Val-Phe
1-adamantyl(1,4-thiazinan-4-yl)methanone
Bicyclo prostaglandin E2
Neotuberostemonine
L-Pyroglutamic acid
PC (16:2e/2:0)
(+/-)12(13)-DiHOME
Coumarin
Nor-9-carboxy-Δ9-THC
Oleoyl ethylamide
LPE 18:3
D-(-)-Quinic acid
α-Linolenic acid
Ferruginol
Stercobilin
Palmitoleic Acid
Acetophenone
Stearamide
Octadecanedioic acid
Pheophorbide A
Isorhapontigenin
Reserpine
Hexadecanamide
Momordin Ic
Ajugol
Oleamide
Phenmetrazine
Indole-3-acrylic acid
SM (d14:3/12:1)
DL-Tryptophan
Ecgonine methyl ester
Class
Alcohols and polyols
Benzene and substituted derivatives
Carbonyl compounds
Carboxylic acids and derivatives
Cinnamic acids and derivatives
Coumarins and derivatives
Fatty Acyls
Flavonoids
Glycerolipids
Indoles and derivatives
Isoquinolines and derivatives
Organonitrogen compounds
Organooxygen compounds
Oxazinanes
Steroids and steroid derivatives
Stilbenes
Tetrapyrroles and derivatives
unclassified
Yohimbine alkaloids
ESI(-)
13(S)-HOTrE
12-oxo Leukotriene B4
LPC 18:2
D-(+)-Malic acid
2-Methylpentanedioic acid
3,8,9-trihydroxy-10-propyl-3,4,5,8,9,10-hexahydro-2H-oxecin-2-one
3-methyl-4-[2-(2-methylphenyl)hydrazono]-4,5-dihydro-1H-pyrazol-5-one
Ixoside
4,5-Dicaffeoylquinic acid
LPC 16:0
LPC 18:1
Gambogellic acid
PS (6:0/16:2)
N-Acetyl-L-phenylalanine
MGDG (2:0/16:3)
Przewaquinone A
D-Phenylalanine
PE (8:0/15:1)
PA (8:0/22:3)
(±)9(10)-DiHOME
Tricin 5-O-hexoside derivative
Succinic acid
alpha-Zearalanol
Gentiopicrin
MGDG (2:0/16:2)
Orcinol gentiobioside
Dihydrocucurbitacin F
Quercetin-3β-D-glucoside
Catechin
Dihydroroseoside
PI (2:0/20:4)
Arabidopyl ketoadipic acid
Plantagoside
3-O-p-coumaroyl shikimic acid O-hexoside
Fumaric acid
8-O-Acetylharpagide
PI (3:0/18:4)
3-Hydroxysebacic acid
Paeishin E
Mevalonic acid
PI (2:0/22:3)
12-Oxo phytodienoic acid
MGDG (20:4/18:5)
MGDG (4:0/22:3)
MGDG (2:0/16:4)
6α-Hydroxyestrone
Mangostin
PA (2:0/22:3)
Bilobalide
Elaidic acid
Azelaic acid
4-Oxoproline
LPA 17:0
Esculin
1a,1b-Dihomo prostaglandin F2α
Nystose
Suberic acid
2-Hydroxyvaleric acid
13-HPODE
Salvinorin B
GlcADG (22:3-18:5)
Aminophylline
Class
Alcohols and polyols
Benzopyrans
Carboxylic acids and derivatives
Coumarins and derivatives
Fatty Acyls
Flavonoids
Hydroxy acids and derivatives
Imidazopyrimidines
Macrolides and analogues
Organooxygen compounds
Prenol lipids
unclassified

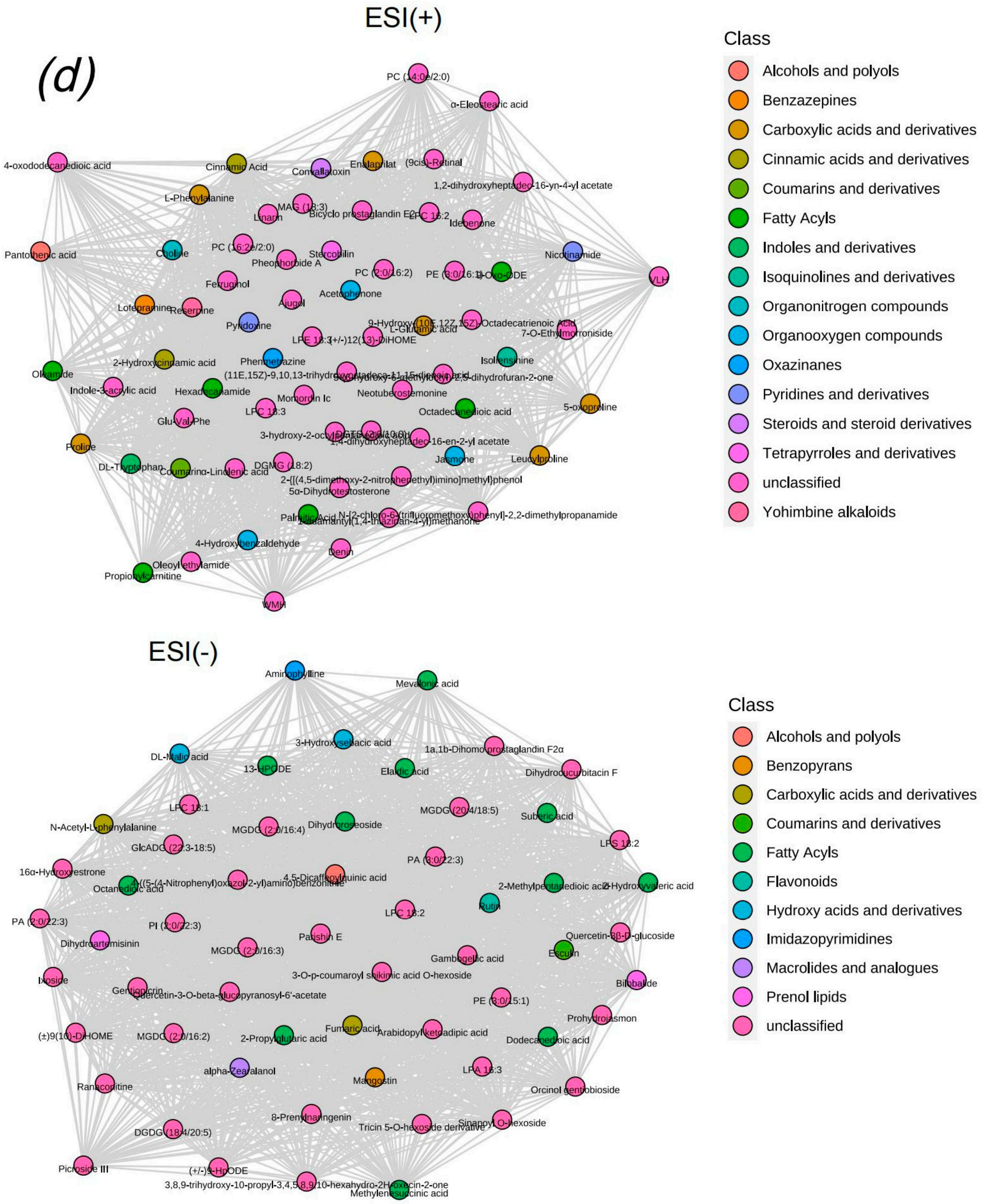
(d)
ESI(+)
Class
Alcohols and polyols
Benzazepines
Carboxylic acids and derivatives
Cinnamic acids and derivatives
Coumarins and derivatives
Fatty Acyls
Indoles and derivatives
Isoquinolines and derivatives
Organonitrogen compounds
Organooxygen compounds
Oxazinanes
Pyridines and derivatives
Steroids and steroid derivatives
Tetrapyrroles and derivatives
unclassified
Yohimbine alkaloids
ESI(-)
Class
Alcohols and polyols
Benzopyrans
Carboxylic acids and derivatives
Coumarins and derivatives
Fatty Acyls
Flavonoids
Hydroxy acids and derivatives
Imidazopyrimidines
Macrolides and analogues
Prenol lipids
unclassified

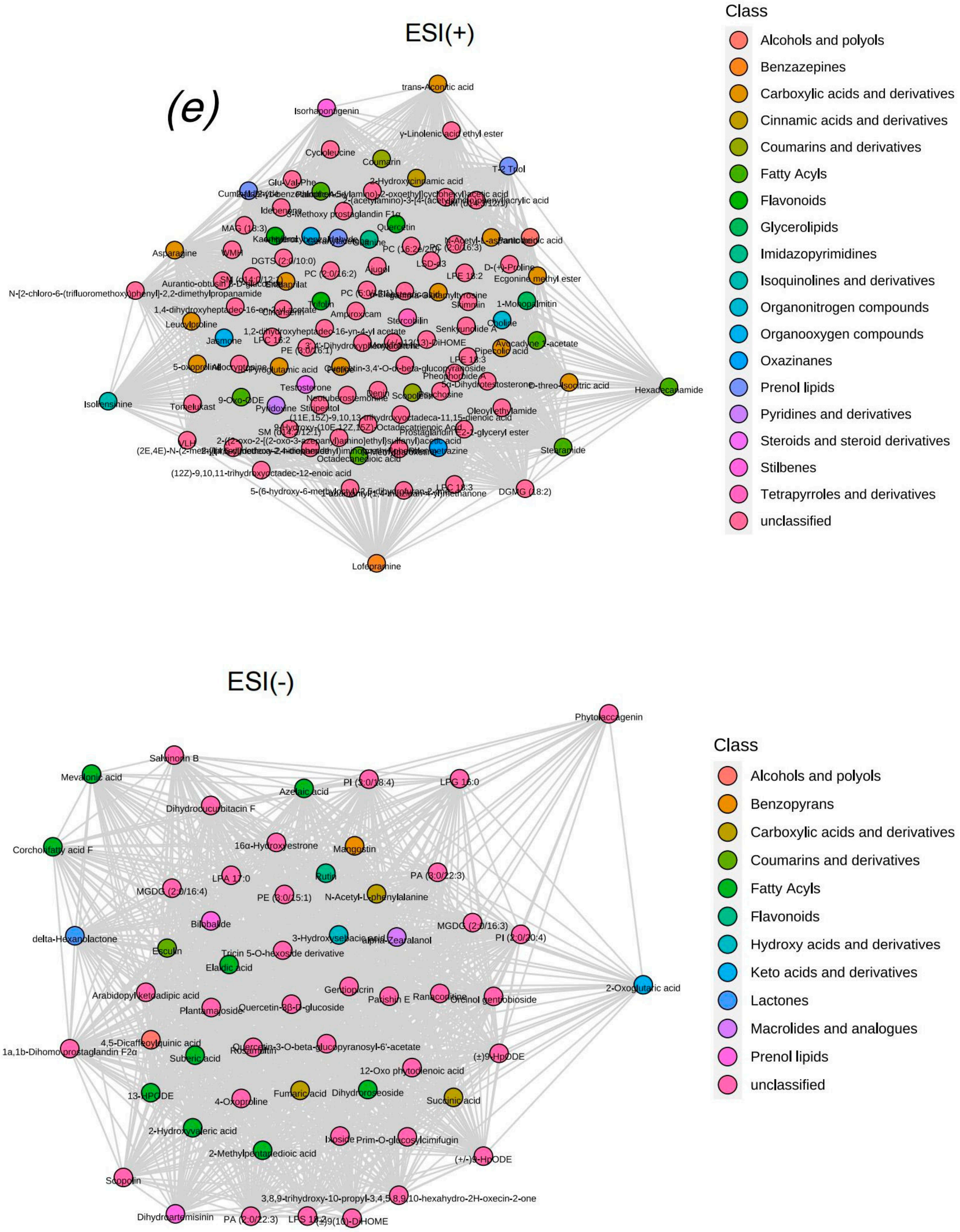
(e)
ESI(+)
Class
Alcohols and polyols
Benzazepines
Carboxylic acids and derivatives
Cinnamic acids and derivatives
Coumarins and derivatives
Fatty Acyls
Flavonoids
Glycerolipids
Imidazopyrimidines
Isoquinolines and derivatives
Organonitrogen compounds
Organooxygen compounds
Oxazinanes
Prenol lipids
Pyridines and derivatives
Steroids and steroid derivatives
Stilbenes
Tetrapyrroles and derivatives
unclassified
trans-Aconitic acid
Isorhapontigenin
γ-Linolenic acid ethyl ester
Cycloleucine
Coumarin
T-2 Triol
Glu-Val-Phe
2-Hydroxycinnamic acid
Idebenone
3-Methoxy prostaglandin F1α
Quercetin
Asparagine
N-[2-chloro-6-(trifluoromethoxy)phenyl]-2,2-dimethylpropanamide
Leucylproline
Jasmone
Isolensinine
Tomelukast
Hexadecanamide
Ecgonine methyl ester
1-Monopalmitin
Choline
Stercobilin
Senkyunolide A
Avocadyne 1-acetate
Pipecolic acid
Testosterone
Pyridoxine
Stilbentol
Oleoylethylamide
Stearamide
(12Z)-9,10,11-trihydroxyoctadec-12-enoic acid
Octadecanedioic acid
DGMG (18:2)
Lofepramine
ESI(-)
Class
Alcohols and polyols
Benzopyrans
Carboxylic acids and derivatives
Coumarins and derivatives
Fatty Acyls
Flavonoids
Hydroxy acids and derivatives
Keto acids and derivatives
Lactones
Macrolides and analogues
Prenol lipids
unclassified
Phytolaccagenin
Salvinorin B
Mevalonic acid
PI (8:0/18:4)
LPG 16:0
Azelaic acid
Dihydrocucurbitacin F
Corchorifatty acid F
16α-Hydroxyestrone
Mangostin
LPA 17:0
Rutin
PA (8:0/22:3)
MGDG (2:0/16:4)
PE (8:0/15:1)
N-Acetyl-L-phenylalanine
Bilobalide
MGDG (2:0/16:3)
delta-Hexanolactone
Esculin
alpha-Zearalanol
PI (2:0/20:4)
Tricin 5-O-hexoside derivative
Elaidic acid
Gentiopicrin
2-Oxoglutaric acid
Arabidopyl ketoadipic acid
Quercetin-3β-D-glucoside
Plantamajoside
Orcinol gentiobioside
4,5-Dicaffeoylquinic acid
1a,1b-Dihomo prostaglandin F2α
Suberic acid
(±)9-HpODE
12-Oxo phytodienoic acid
13-HpODE
4-Oxoproline
Fumaric acid
Dihydroroseoside
Succinic acid
2-Hydroxyvaleric acid
Prim-O-glucosylcimifugin
2-Methylpentanedioic acid
(+/-)9-HpODE
Scopolin
3,8,9-trihydroxy-10-propyl-3,4,5,8,9,10-hexahydro-2H-oxecin-2-one
Dihydroartemisinin
PA (2:0/22:3)
(±)9(10)-DiHOME

Figure S10. Differential metabolites network analysis from various Mg supply in mulberry. (a) CK-T1 (b) CK-T2 (c) CK-T3 (d) CK-T4 (e) CK-T5. The network was built using the differential metabolites classes.

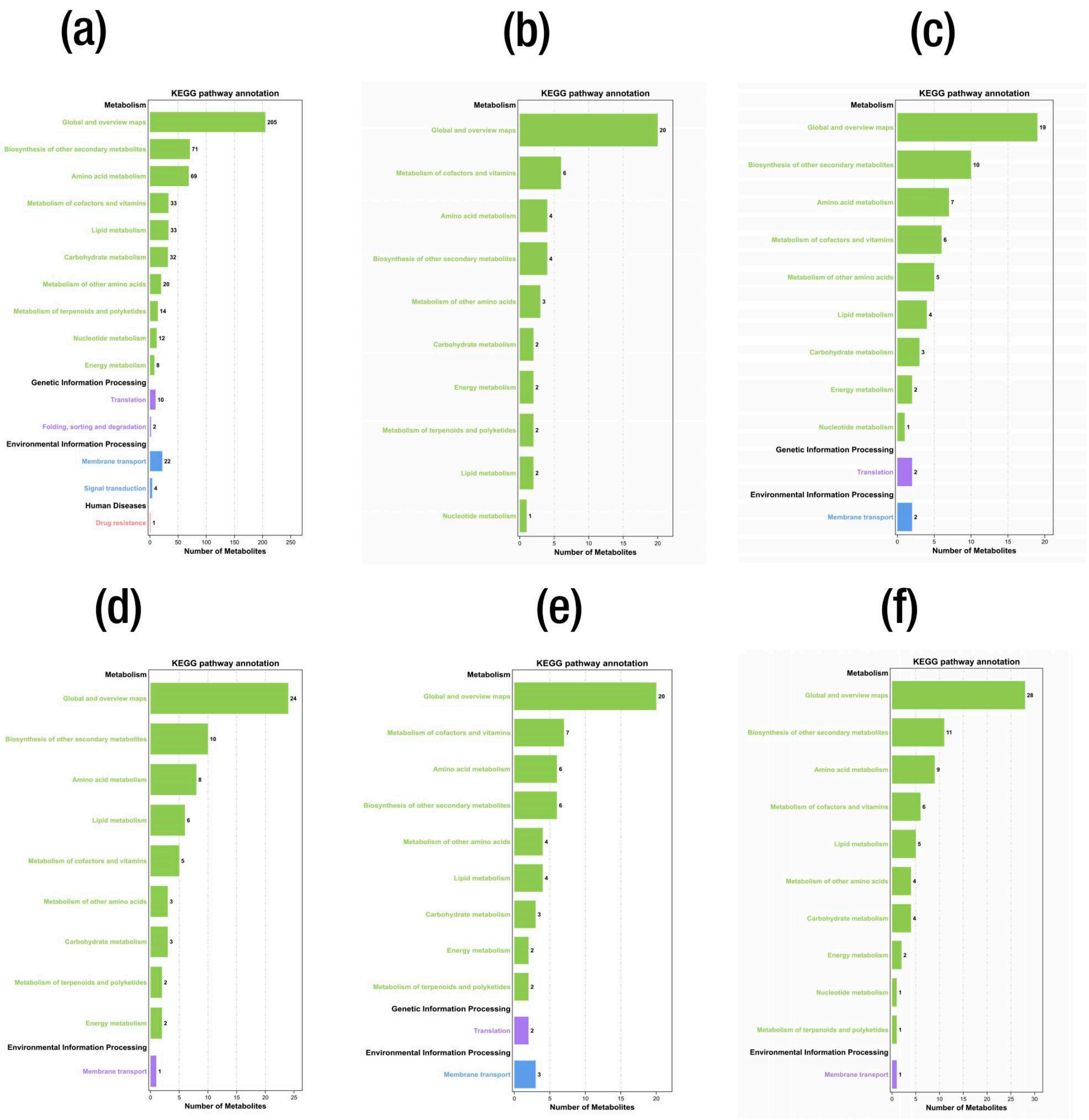

Figure S11. Differential metabolites KEGG annotation from various Mg application in mulberry. (a) general KEGG annotation of all the extracted metabolites (b) differential in CK-T1 (c) differential in CK-T2 (d) differential in CK-T3 (e) differential in CK-T4 (f) differential in CK-T5. KEGG pathways were retrieved from www.kegg.jp/kegg/kegg1.html.

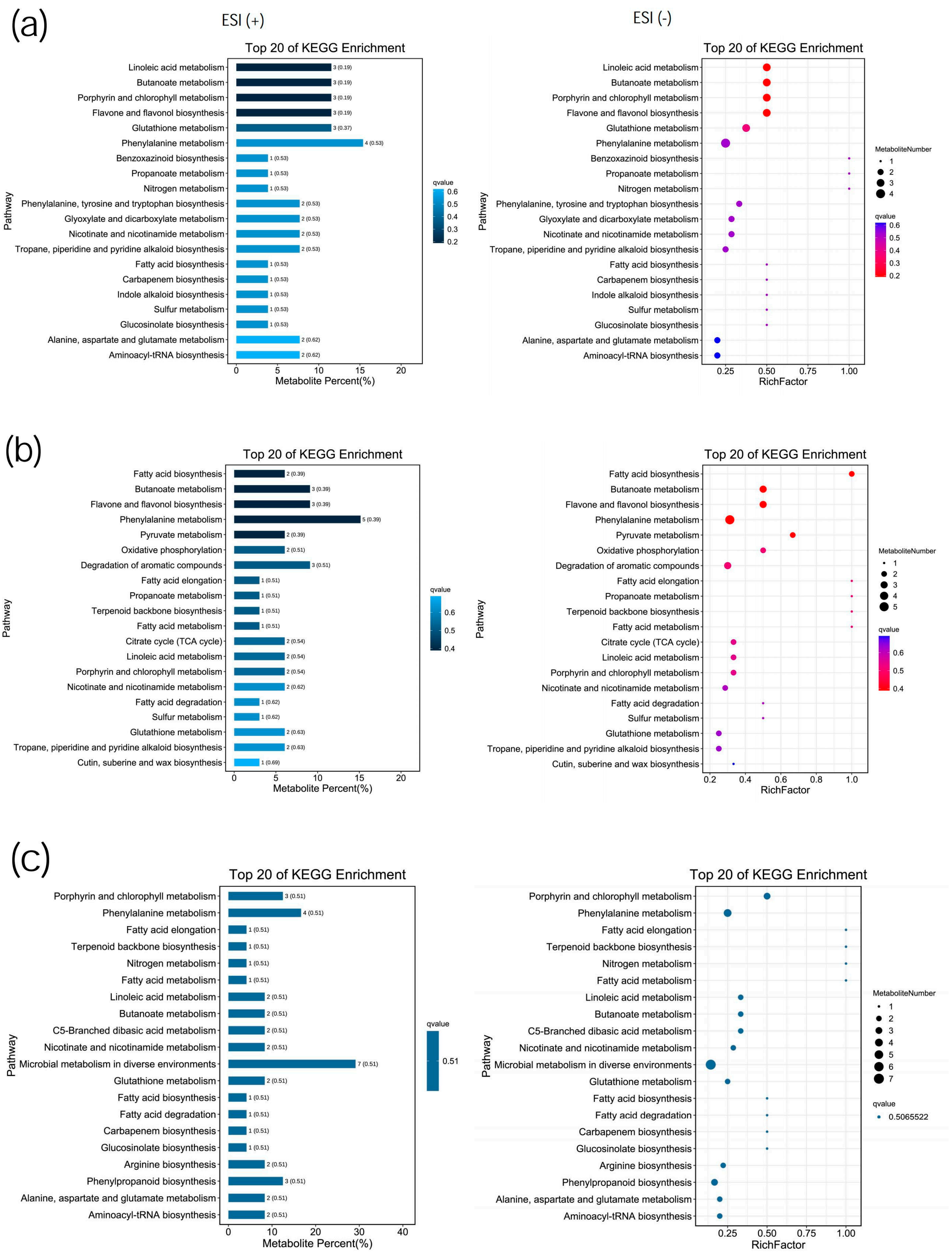

(a)
ESI (+)
ESI (-)
Top 20 of KEGG Enrichment
Linoleic acid metabolism
Butanoate metabolism
Porphyrin and chlorophyll metabolism
Flavone and flavonol biosynthesis
Glutathione metabolism
Phenylalanine metabolism
Benzoxazinoid biosynthesis
Propanoate metabolism
Nitrogen metabolism
Phenylalanine, tyrosine and tryptophan biosynthesis
Glyoxylate and dicarboxylate metabolism
Nicotinate and nicotinamide metabolism
Tropane, piperidine and pyridine alkaloid biosynthesis
Fatty acid biosynthesis
Carbapenem biosynthesis
Indole alkaloid biosynthesis
Sulfur metabolism
Glucosinolate biosynthesis
Alanine, aspartate and glutamate metabolism
Aminoacyl-tRNA biosynthesis
Pathway
Metabolite Percent(%)
RichFactor
qvalue
MetaboliteNumber
(b)
Top 20 of KEGG Enrichment
Fatty acid biosynthesis
Butanoate metabolism
Flavone and flavonol biosynthesis
Phenylalanine metabolism
Pyruvate metabolism
Oxidative phosphorylation
Degradation of aromatic compounds
Fatty acid elongation
Propanoate metabolism
Terpenoid backbone biosynthesis
Fatty acid metabolism
Citrate cycle (TCA cycle)
Linoleic acid metabolism
Porphyrin and chlorophyll metabolism
Nicotinate and nicotinamide metabolism
Fatty acid degradation
Sulfur metabolism
Glutathione metabolism
Tropane, piperidine and pyridine alkaloid biosynthesis
Cutin, suberine and wax biosynthesis
Pathway
Metabolite Percent(%)
RichFactor
qvalue
MetaboliteNumber
(c)
Top 20 of KEGG Enrichment
Porphyrin and chlorophyll metabolism
Phenylalanine metabolism
Fatty acid elongation
Terpenoid backbone biosynthesis
Nitrogen metabolism
Fatty acid metabolism
Linoleic acid metabolism
Butanoate metabolism
C5-Branched dibasic acid metabolism
Nicotinate and nicotinamide metabolism
Microbial metabolism in diverse environments
Glutathione metabolism
Fatty acid biosynthesis
Fatty acid degradation
Carbapenem biosynthesis
Glucosinolate biosynthesis
Arginine biosynthesis
Phenylpropanoid biosynthesis
Alanine, aspartate and glutamate metabolism
Aminoacyl-tRNA biosynthesis
Pathway
Metabolite Percent(%)
RichFactor
qvalue
0.5065522
MetaboliteNumber

Figure S12. Differential metabolites KEGG enrichment analysis of the top 20 pathways from Mg treatments in mulberry. (a) CK-T2 (b) CK-T3, (c) CK-T4. The bar graph is the metabolites percentages in the pathways and the bubble plot on the right side represents enrichment factor of the metabolites in the pathways using the smallest q-value.
